# Artificial Intelligence in Schizophrenia Spectrum Disorders: Current Use and Future Perspectives. A Systematic Mapping Literature Review

**DOI:** 10.31083/AP45104

**Published:** 2026-04-23

**Authors:** Andrea Zucchetti, Viola Bulgari, Cecilia Davini, Marco Ghio, Marisa Dosoli, Michela Gregorelli, Gabriele Nibbio, Stefano Barlati, Antonio Vita

**Affiliations:** ^1^Department of Mental Health and Addiction Services, ASST Spedali Civili of Brescia, 25123 Brescia, Italy; ^2^Department of Clinical and Experimental Sciences, University of Brescia, 25121 Brescia, Italy

**Keywords:** artificial intelligence, deep learning, machine learning, natural language processing, schizophrenia

## Abstract

**Background:**

Artificial intelligence (AI) techniques are increasingly applied in psychiatric research, yet their use in schizophrenia spectrum disorders (SSDs) remains heterogeneous. This systematic mapping literature review aimed to examine the current applications of AI systems in SSDs. AI approaches were categorized into natural language processing (NLP), machine learning (ML), and deep learning (DL). Key study characteristics were systematically analyzed, including publication year, type of AI algorithm, study population, clinical outcomes, and instrumental techniques. Trends in journal impact factor (IF) were also evaluated.

**Methods:**

A systematic mapping approach was employed to identify and categorize relevant studies investigating AI applications in SSDs. Included studies were analyzed according to predefined evaluation criteria to provide an overview of methodological trends and research focus within the field.

**Results:**

A total of 853 studies met the inclusion criteria. Machine Learning emerged as the most frequently utilized AI approach. AI methods were predominantly applied to diagnostic and differential diagnostic tasks in SSDs. The most commonly employed instrumental techniques were magnetic resonance imaging (MRI) and electroencephalography (EEG). Journal Impact Factor varied significantly across study characteristics, with higher IFs observed in studies focusing specifically on schizophrenia, reporting diagnostic outcomes, or employing MRI or EEG-based measures.

**Conclusions:**

This review represents the first systematic mapping literature review to comprehensively examine AI applications in schizophrenia spectrum disorders. The findings may support clinicians and researchers in planning, implementing, and evaluating AI-based methodologies in SSD research and clinical contexts. Nevertheless, further studies are needed to establish the clinical validity, robustness, and translational applicability of these algorithms.

## Main Points

1. The application of AI in Schizophrenia Spectrum Disorders has progressively increased, reflecting growing scientific interest.

2. Machine Learning is the most frequently employed AI approach, primarily supporting diagnostic and differential diagnostic processes.

3. Studies mainly focus on schizophrenia and utilize neuroimaging (particularly MRI) and neurophysiological techniques (particularly EEG) to identify neurobiological correlates.

4. Journal Impact Factors display an upward trend, being higher in studies addressing schizophrenia, reporting diagnostic outcomes, or utilizing MRI/EEG techniques.

5. This review provides a comprehensive overview of AI in SSDs, offering guidance for clinicians and researchers and highlighting the need for further validation studies.

## 1. Introduction

The term “Artificial Intelligence” was coined by John McCarthy in 1956, who defined AI as “the science and engineering of making machines intelligent” [1]. However, the concept of AI can be traced back to 1950, when mathematician Alan Turing proposed the term “Turing test” in his paper “Computing Machinery and Intelligence” published in the journal Mind [2]. The Turing test is a method for determining whether a machine can think, and it is still used today to assess the intelligence of different machines. In the aforementioned article, Turing proposed the hypothesis of the possibility of realising or conceiving a machine that would possess the “thinking characteristics” that define human thought. This is a concept that remains challenging to define in the present day. It is acknowledged that human thought is an abstract concept. On this basis, Turing hypothesised the creation of a machine that would act in such a way as to satisfy the requests made to it.

Several variants were then theorized and implemented over the years. Among these variants there was the Eliza program, developed by Joseph Weizenbaum in the 1960s [3]. The Eliza program functions as a psychotherapist by engaging in dialogue with the interlocutor. It is based on the concept of “something very different” which refers to the search for key words within a sentence. These key words then lead to the formulation of an answer according to pre-established rules. If key words are not identified, the program either reiterates the subject’s statement by modifying it or generates context-free remarks to re-initiate the dialogue. A notable feature of Eliza was its capacity for learning, which enabled it to store the sentences expressed by interlocutors in its memory, thereby expanding the repertoire of potential responses.

In 1985, Frank Rosenblatt constructed the Mark 1 Perceptron, a pioneering computer model inspired by the organization of the nervous system. This model employed a trial-and-error mechanism to facilitate learning, marking a significant advancement in the field of artificial intelligence (AI). Approximately a decade later, Marvin Minsky and Seymour Papert published a seminal book entitled Perceptrons [4], which was instrumental in the subsequent development of neural networks. These networks were used to develop specific AI algorithms that are still in use today, such as ChatGPT [5].

There is currently no single definition of the concept of AI [6], so there are multiple definitions that can be simplified into four categories [7]: systems that think like humans, systems that act like humans, systems that think rationally, and systems that act rationally. If we want to try to condense everything into a single definition, we have to refer to Dimiter Dobrev who said “Artificial Intelligence will be a programme that in an arbitrary world will fare no worse than a human being” [8]. To simplify this definition, we can define AI as an algorithm that moves within programmed and predefined scenarios chosen by the developer (‘arbitrary world’) to which requests are submitted and which will provide a solution based on its own resources and capabilities. It can therefore be said that the possibilities within which a machine can move are always pre-determined from the outset, the results provided will never go beyond specific logical or mathematical laws imposed by the developer, it therefore does not possess the dimension of creativity [9].

Currently, the possible applications of AI algorithms are manifold [10]. Efforts are underway to implement them in the domains of medicine as for example in the prognosis and treatment plans for cancer patients and mental health [11, 12, 13, 14, 15].

The application of AI has been and continues to be the subject of great attention in psychiatric research [16]. The results agree in underlining the usefulness of applying AI methodology to facilitate diagnosis, suggest prognosis and the best treatment course [15]. Other studies highlighted the effectiveness of AI-driven tools, such as chatbots and predictive modeling and in improving patient engagement even if they recognize the need for greater methodological rigor [14].

In light of the extensive use of AI in mental health and psychiatry research, AI has been applied to multiple aspects of schizophrenia, including assessment and prediction of the disorder [17, 18, 19]. The Schizophrenia Spectrum Disorders, as defined in the DSM-5-TR, includes schizophrenia, other psychotic disorders (e.g., delusional disorder, brief psychotic disorder, schizophreniform disorder, schizoaffective disorder, substance-induced psychotic disorder, and psychotic disorder due to another medical condition), and schizotypal personality disorder [20]. These conditions are characterized by abnormalities in one or more domains — delusions, hallucinations, disorganized thinking (formal thought disorder), disorganized or abnormal motor behaviour (including catatonia), and negative symptoms (affective flattening, anhedonia, asociality, avolition, apathy, and alogia) — and are frequently accompanied by cognitive deficits, which have become an increasing focus of research in recent years [21]. In their review, Lai *et al*. (2021) [17] reported that most studies have focused primarily on detecting and diagnosing schizophrenia, while fewer have analyzed AI applications in clinical interventions; this is consistent with findings from a review of social media data for the diagnosis of psychotic disorders [22]. There remains a need for comparative studies integrating composite data and advanced AI techniques [17]. Regarding outcome prediction, AI appears promising, although heterogeneity in treatment-response definitions, populations, and interventions limits comparability across studies [23]. The objective of our study is to identify all published articles including subjects diagnosed with Schizophrenia Spectrum Disorders and employing one or more of three AI subtypes — natural language processing (NLP), machine learning (ML), and deep learning (DL).

### 1.1 Natural Language Processing (NLP)

The field of NLP emerged in the 1950s, a product of the confluence of AI, linguistics, and mathematics. It is an analysis technique that enables the automatic decoding and understanding of language with the support of machines [24]. The field is subdivided into two distinct domains: the first is natural language understanding (NLU), which translates natural language into a machine-understandable language [25]; the second is natural language generation (NLG), which produces a natural language output from structured data (e.g., text to text, text to other and vice versa) [26]. The process used by NLP consists of four parts: pre-processing of the text, representation of the text, training of the model, and evaluation of the model. In the pre-processing stage, the text is simplified and corrected to enhance its accuracy and efficiency in the subsequent steps. Subsequently, the text is converted into numerical values, which are then used to train the algorithm. The efficacy of the algorithm is then evaluated in real-world settings [27].

### 1.2 Machine Learning (ML)

The algorithms encompassed within the domain of ML are based on the principle of “learning”, understood as the process of acquiring information through observation and experience, which is subsequently used to infer generalizable patterns or rules and apply them to new data.

Machine learning can be categorized into three distinct models [9]: Supervised Learning, where the machine has access to all the data and is tasked with the construction of a function that elucidates the underlying phenomenon; Reinforcement Learning, where the machine is provided with data sequentially; and Unsupervised Learning, where the machine is endowed with all the input and output data, yet it is not subdivided. In addition to these three categories, there are also systems known as Semi-Supervised Learning (a combination of Supervised and Unsupervised) [28] and Deep Learning.

### 1.3 Deep Learning (DL)

Deep Learning is a sub-type of Machine Learning that utilizes a system of operation based on the structure of neural networks (neural networks), wherein learning occurs through continuous “training”. The term “deep neural networks” (DNN) refers to the architecture of these networks, which is based on simple units called “artificial neurons” (ANN). These artificial neurons are combined in interconnected layers (typically three or more, although recent algorithms can have hundreds of layers). The term “deep” in “deep neural networks” refers to the depth of this network architecture. This phenomenon mirrors the process of learning in the human nervous system, wherein the strength of connections between neurons, or “weights”, is adjusted, allowing for the establishment of more functional connections that are prioritized [29]. Artificial neural networks process input data in a non-linear manner, exhibiting characteristics like those of brain neurons, such as activation only when a threshold potential is reached, which is determined by the weighted sum of the inputs, and subsequent output generation.

It is imperative to underscore the fundamental distinction between the operational principles of deep learning and those of non-deep learning machine learning algorithms. The former is predicated on the processing of unlabeled (“raw”) data, whereas the latter is designed to extrapolate and select data based on the inputs provided by the programmer. A critical aspect of deep learning is the inherent processing and extrapolation of data by the algorithm itself, which is subsequently utilized to formulate responses to queries. In contrast, non-deep learning machine learning algorithms are constrained to the utilization of structured data with a substantial sample size, such as electroencephalography (EEG) or resting-state functional MRI data.

### 1.4 Aims

The primary aim of this review was to provide a comprehensive and systematic examination of how AI algorithms have been applied over the years in studies involving individuals diagnosed with schizophrenia spectrum disorders (SSDs). In particular, the review sought to map key characteristics of these studies, including the year of publication, the type of AI algorithm employed, the populations under investigation, the reported outcomes, and the instrumental techniques utilized. Focusing on outcomes and methodological approaches was intended to create a structured framework that could guide and inform future research in the field. As a secondary objective, the review also aimed to explore whether any of these study characteristics were associated with journal Impact Factor (IF), thereby providing insight into the topics currently attracting the greatest scientific attention.

## 2. Materials and Methods

This systematic literature review was conducted in accordance with the 2020 PRISMA Guidelines [30] (See **supplementary material**).

### 2.1 Search Strategy

Peer-reviewed articles were identified through searches of three electronic databases: PubMed, Scopus, and PsycINFO. Searches were conducted on titles, abstracts, and keywords using a strategy adapted for each database to optimize retrieval, as follows:

• Pubmed: (machine learning OR artificial intelligence OR deep learning OR natural language processing OR neural network OR unsupervised learning OR supervised learning OR data mining) AND (schizophrenia OR psychosis OR psychotic).

• Scopus: ((machine AND learning) OR (artificial AND intelligence*) OR (deep AND learning) OR (natural AND language AND processing) OR (neural AND network*) OR (unsupervised AND learning) OR (supervised AND learning) OR (data AND mining)) AND ((schizophrenia) OR (psychosis) OR (psychotic)).

• PsycINFO: ((machine AND learning) OR (artificial AND intelligence*) OR (deep AND learning) OR (natural AND language AND processing) OR (neural AND network*) OR (unsupervised AND learning) OR (supervised AND learning) OR (data AND mining)) AND ((schizophrenia) OR (psychosis) OR (psychotic)).

The present systematic review included studies published in peer-reviewed journals up to December 2, 2024. Only publications written in English were eligible for inclusion.

### 2.2 Inclusion Criteria

According to PICOS Reporting System [31] we defined the following:

- Population: studies with subjects diagnosed with a SSD were included. Papers on early or first-episode psychosis (FEP) were included, reflecting the growing research focus on this population. Those focusing on brief psychotic disorder, substance/medication-induced psychosis, or psychotic disorders secondary to other medical conditions were excluded due to their heterogeneous and typically transient nature, which may limit comparability with other SSDs.

- Intervention: studies employing AI algorithms belonging to the three categories described above (NLP, ML, and DL) were retained.

- Comparison: no pre-specified comparison groups were defined for this review. The only analytical comparison performed involved examining the distribution of journal IF across the study variables, in order to explore potential associations between IF and study characteristics.

- Outcomes: any results obtained, divided into the categories described later.

- Study: was not limited to a specific design; controlled and uncontrolled clinical studies, cohort studies, prospective case-control studies, and cross-sectional studies were considered for inclusion.

### 2.3 Outcome Measures 

In line with the objectives of this review, a quantitative analysis was conducted on specific study parameters, including publication year, type of AI employed, study population, primary outcomes, and the instrumental techniques utilized.

A timeline analysis was conducted to describe the temporal evolution of the included publications. This analysis was performed systematically using the extracted data, aggregating studies by year of publication and reporting variations in the volume of scientific output over the examined period.

AI approaches were categorized as ML, DL, or NLP. When studies integrated multiple AI paradigms, the classification was based on the most specific algorithmic category; for instance, studies combining ML and DL methods were assigned to the DL category, as DL represents a specialized subset of ML techniques.

Study populations were divided into five categories: FEP, schizophrenia, SSDs including schizophrenia, SSDs excluding schizophrenia, and schizotypal personality disorder.

The outcomes of individual studies were organized into four overarching domains:

∙ Neurobiological correlates, encompassing the identification of brain regions and circuits potentially pathognomonic for the condition, estimation of brain age, and characterization of nervous system aging trajectories;

∙ Clinical characterization, including correlations of clinical, biological, behavioral, and functional parameters, such as psychopathological features, physical comorbidities, self- and hetero-injurious behaviors, objective functioning (e.g., service utilization), and subjective well-being;

∙ Diagnosis, covering both differentiation from healthy controls and the identification of differential diagnoses relative to other pathologies;

∙ Prognosis, addressing disease evolution, treatment outcomes (pharmacological and psychotherapeutic), treatment adherence, and prediction of disease progression or risk of transition.

Instrumental techniques employed for data collection were grouped as follows:

∙ Neurobiological measurements, including structural and functional neuroimaging (MRI, fMRI, PET, magnetoencephalography) and neurophysiological assessments (EEG);

∙ Audio/video recordings, captured during interviews or standardized tests;

∙ Biological samples, such as blood or saliva, analyzed for genetic, inflammatory, renal, hepatic, or metabolic markers;

∙ Other, comprising somatic measurements (e.g., height, weight, BMI) and movement tracking (e.g., eyes, head, limbs) using mobile applications equipped with gyroscopes and GPS sensors;

∙ No instrumental methods, for studies that relied exclusively on socio-demographic or psychometric data obtained through standardized tests and scales.

Studies employing multimodal datasets (e.g., combining MRI, EEG, or genetic data) were included by disaggregating the modalities and coding each instrumental technique separately.

Finally, journal IF was analyzed in relation to publication year. IF values were obtained from Journal Citation Reports (Clarivate Analytics). When the IF for the year of publication was unavailable, the value from the closest preceding year was used. This allowed us to assess potential associations between study characteristics and journal impact over time.

### 2.4 Data Extraction

Following the removal of duplicates, the screening process—based on titles and abstracts—was independently conducted by two reviewers, working in pairs randomly assigned from a group of five (VB, CD, MD, MGre, MGhi). Any discrepancies or uncertainties were resolved by a sixth researcher (AZ), who served as adjudicator. Data extraction was subsequently carried out independently by four reviewers (VB, CD, MGre, MGhi), with the supervising author (AZ) performing a final verification to ensure consistency and accuracy across all extracted data. In addition, the reference lists of all included studies were manually reviewed to identify further relevant articles not captured by the electronic search. This multi-step, independent review process was designed to strengthen methodological rigor and minimize potential bias in study selection and data extraction.

### 2.5 Statistical Analysis

All statistical analyses were performed using R (A language and environment for statistical computing. Version 4.4.2 (2024-10-31 ucrt). R Foundation for Statistical Computing, Vienna, Austria. https://www.r-project.org/), RStudio (Integrated Development Environment for R. Version 2025.05.0. Posit Software, PBC, Boston, MA, USA. https://posit.co/). A *p*-value of 0.05 or less was considered statistically significant. The primary objective was to analyze the entire database by describing the absolute and relative frequencies of each variable. As a secondary objective, a bibliometric analysis was conducted to assess the distribution of the IF across selected variables. For each study, the IF of the journal in which it was published during the year of publication was assigned in order to objectively assess the scientific impact of each paper [32, 33, 34]. Because the IF distribution was non-normal (as indicated by the Kolmogorov–Smirnov and Shapiro–Wilk tests, both significant at *p* < 0.001), non-parametric tests were applied. Spearman’s rank correlation coefficient (R) was used to assess correlations involving IF over the years. For the remaining variables, the Kruskal–Wallis test was employed, followed, when significant, by pairwise comparisons using the Mann–Whitney (MW) test with Bonferroni correction. Studies reporting multiple outcomes or instrumental techniques were analyzed by treating each outcome or technique as a separate observation to provide a more detailed examination of methodological patterns and research trends. Although this approach may introduce some degree of non-independence among data points, the proportion of such cases was low, and its impact on the overall validity of the findings was considered minimal.

## 3. Results

**Fig. 1.**
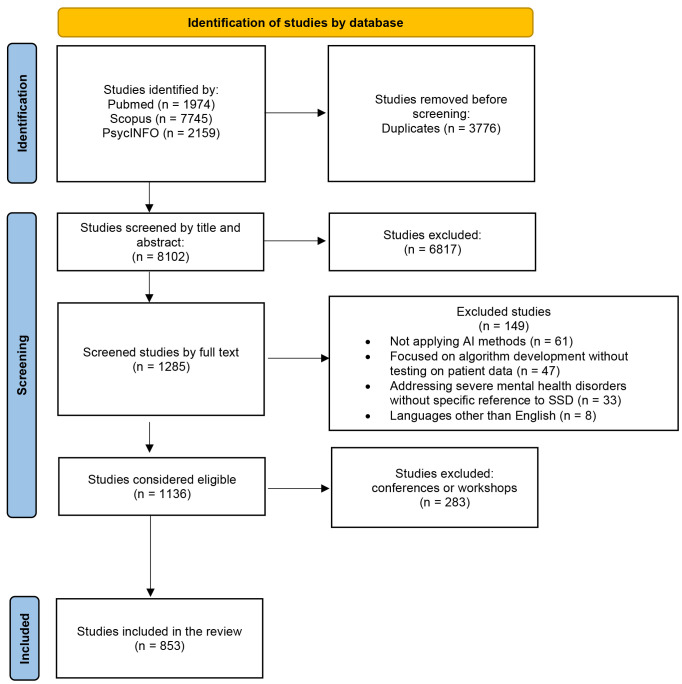
**PRISMA 2020 flow diagram for new systematic reviews that included searches of databases and registers only**. PRISMA, preferred reporting items for systematic reviews and meta-analyses.

The results of the systematic search are reported in Fig. 1, following the PRISMA Flow Diagram 2020 indications [30].

A total of 11,878 original records were identified. Of these, 3776 were duplicates and were automatically deleted. The remaining 8102 articles were initially screened by title and abstract. This resulted in the exclusion of 6817 articles. Of the remaining 1285, full-text analysis and data extraction were performed. At the end of the screening procedure, 1136 articles were eligible for this systematic review. Following the thorough review of the articles, it was determined that 283 of them (24.91%) were studies that had been presented at conferences or workshops; these studies were not subsequently published in journals. Consequently, they were excluded from further consideration, and the final number of analysed studies was 853 articles (see **Supplementary Material A** for a complete list of included studies, categorised by year of publication).

### 3.1 Years of Publication

**Fig. 2.**
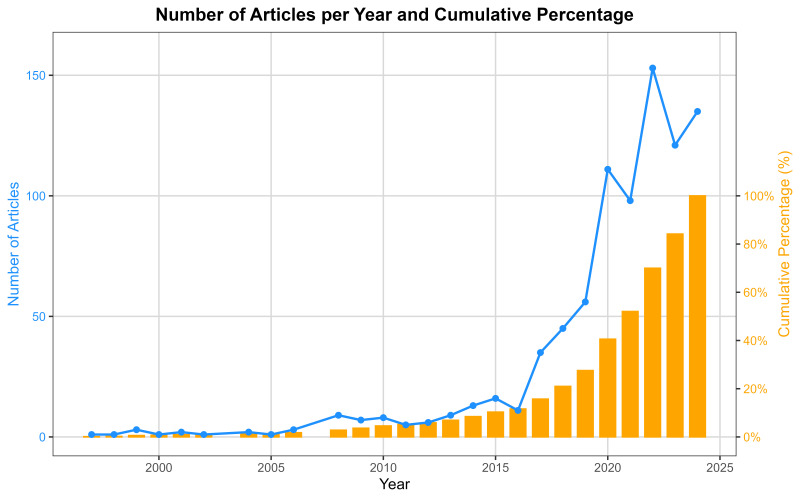
**Number of articles per year and cumulative percentage**.

The temporal distribution of publications reveals a markedly accelerated growth of the field. As shown in Fig. 2, the earliest studies appeared in 1997 [35], followed by over a decade of sparse production, with annual outputs generally below ten articles. A substantive increase became evident after 2014, with a progressive escalation culminating in a sharp expansion from 2017 onwards. Annual publications rose from 35 in 2017 to 111 in 2020, ultimately reaching their peak in 2024 with 135 articles (15.83% of the overall sample). As detailed in Table 1, the cumulative distribution indicates that approximately half of all included studies had been published by the end of 2021 (52.05%), while the remaining 48% were produced in the subsequent three-year period, underscoring the rapid recent growth of research activity in this domain.

**Table 1.** **Analysis of publication years**.

Year	Number of articles	Percentage (%)	Cumulative percentage (%)
1997	1	0.12	0.12
1998	1	0.12	0.23
1999	3	0.35	0.59
2000	1	0.12	0.70
2001	2	0.23	0.94
2002	1	0.12	1.06
2004	2	0.23	1.29
2005	1	0.12	1.41
2006	3	0.35	1.76
2008	9	1.06	2.81
2009	7	0.82	3.63
2010	8	0.94	4.57
2011	5	0.59	5.16
2012	6	0.70	5.86
2013	9	1.06	6.92
2014	13	1.52	8.44
2015	16	1.88	10.32
2016	11	1.29	11.61
2017	35	4.10	15.71
2018	45	5.28	20.98
2019	56	6.57	27.55
2020	111	13.01	40.56
2021	98	11.49	52.05
2022	153	17.94	69.99
2023	121	14.19	84.17
2024	135	15.83	100.00

### 3.2 Type of Artificial Intelligence Model

As demonstrated in Table 2, the analysis focused predominantly on ML algorithms (592; 69.4%), with a subsequent emphasis on DL (215; 25.2%) and NLP (46; 5.4%).

**Table 2.** **Analysis of the type of Artificial Intelligence used**.

Type of Artificial Intelligence	Frequency	Percentage (%)
Machine Learnings	592	69.4
Deep Learning	215	25.2
Natural Language Processing	46	5.4
Total	853	100.0

### 3.3 Population

Most of the studies analysed focused on subjects with Schizophrenia (616; 72.2%). Patients with SSDs including Schizophrenia, accounted for 144 of the studies (16.9%), while FEP accounted for 87 (10.2%). However, schizotypal personality disorder and SSDs excluding schizophrenia are poorly studied in AI (0.5% and 0.2%, respectively). The data are shown in Table 3.

**Table 3.** **Analysis of the recruited patients**.

Population	Frequency	Percentage (%)
First-Episode Psychosis	87	10.2
Schizophrenia	616	72.2
SSDs (including schizophrenia)	144	16.9
SSDs (excluding schizophrenia)	2	0.2
Schizotypal Personality Disorder	4	0.5
Total	853	100.0

SSDs, schizophrenia spectrum disorders.

### 3.4 Outcome of Individual Studies

In our systematic review, 101 studies (11.84%) evaluated two objectives, resulting in a total of 954 outcomes. The number of objectives increases to 960 if we analyze them more specifically, as described in the preceding paragraph (107 articles evaluated two objectives (12.54%)). The most common objective is the diagnosis of pathology in relation to healthy controls and other disorders (401; 42%), followed by the identification of neurobiological correlates (229; 24%), clinical characterisation (200; 21%) and prognosis (122; 12.8%). A rigorous analysis of the specific aims indicates that the two most common objectives, following diagnosis and differential diagnosis, are the identification of pathognomonic brain areas and circuits (216; 22.5%) and psychopathological aspects (146; 15.3%). For a more detailed analysis, please refer to Table 4a,4b.

**Table 4a.** **Analysis of study outcomes macro-categories with specifications**.

Outcome	Frequency	Percentage of the total (%)
Neurobiological correlates	229	24.0
Clinical characterization	200	21.0
Diagnosis	401	42.0
Prognosis	122	12.8
Other	2	0.2
Total	954	100.0

**Table 4b.** **Analysis of study outcomes with specifications**.

Outcome		Frequency	Percentage of the total (%)
Neurobiological correlates			
	Identification of brain areas and circuits	216	22.5
	Brain age	15	1.6
Clinical characterization			
	Psychopathological aspects	146	15.2
	Physical comorbidities	20	2.1
	Self-harming dimension	8	0.8
	Heteroaggressive dimension	15	1.6
	Use of Services	7	0.7
	Perception of quality of life	7	0.7
Diagnosis		401	41.8
Prognosis			
	Prognosis in patients	59	6.1
	Response and adherence to therapy	53	5.5
	Transition prediction	11	1.1
Other		2	0.2
Total		960	100.0

### 3.5 Instrumental Techniques Employed in the Study

Out of the 853 studies analysed, 143 (16.76%) did not use instrumental techniques and only collected socio-demographic variables and/or psychopathological aspects. Only one study (0.12%) used three instrumental techniques, 35 studies (4.10%) used two techniques, and the remaining 674 studies (79.01%) used only one technique. The categories were then divided into more specific techniques. The results showed that only 3 studies (0.35%) used three techniques, 49 studies (5.74%) used two techniques, and 658 studies (77.14%) used only one technique.

Neurobiological measurements are the most used instrumental techniques (515; 57.9%), primarily through neuroimaging (383; 42.3%) and to a lesser extent EEG (141; 15.6%). The second most used technique involves biological samples (120; 13.5%), with genetic component evaluation being the most prevalent (84; 9.3%). Audio and video recordings account for 7.6% of the total techniques used. Finally, “other” techniques make up 4.8% of the total in our sample.

For a detailed exposition of the instrumental techniques employed, please refer to Table 5a,5b.

**Table 5a.** **Analysis of instrumental techniques used in studies**.

Instrumental techniques	Frequency	Percentage of the total (%)
Neurobiological measurements	515	57.9
Audio/video recordings	68	7.6
Biological samples	120	13.5
Other	43	4.8
No instrumental techniques	143	16.1
Total	889	100.0

**Table 5b.** **Analysis of instrumental techniques used in studies with specific**.

Instrumental techniques		Frequency	Percentage of the total (%)
Neurobiological measurements			
	Neuroimaging	383	41.9
	EEG	141	15.4
Audio/video recordings		68	7.4
Biological samples			
	Genetic analysis	84	9.2
	Evaluation of other aspects	40	4.4
Other			
	Somatic and physiological measurements	11	1.2
	Motion sensors	21	2.3
	APP on mobile devices	9	1.0
	Special methods	5	0.6
No instrumental methods		152	16.6
Total		914	100.0

### 3.6 Relationship With the Impact Factor

The analysis revealed a very weak positive, yet statistically non-significant correlation between publication year and journal IF (Spearman’s ρ = 0.038, 95% CI [–0.026, 0.108], *p* = 0.263), suggesting no meaningful temporal trend in the IF of published studies.

The non-parametric Kruskal-Wallis test was utilised to evaluate the relationship between the IF and the variables. In instances of statistical significance, subsequent comparisons between the variables were conducted employing the Mann-Whitney (MW) test, which was corrected for Bonferroni.

The variable “instrumental techniques” did not yield statistically significant results (*p* = 0.193), and thus no post-hoc comparisons were performed.

The overall Kruskal-Wallis test for “type of AI” reached statistical significance (*p* < 0.05; η^2^ = 0.006, 95% CI [–0.002, 0.019]), although the effect size was negligible. No pairwise comparison remained significant after Bonferroni correction (α = 0.016). The nominal ML–NLP difference (ML: median 4.3, IQR 3.3–5.3; NLP: median 3.9, IQR 2.9–4.5; uncorrected *p* = 0.019) did not withstand multiple-comparison adjustment and therefore should not be interpreted as substantively meaningful (detailed results are provided in **Supplementary Table 1**, included in the **Supplementary Material B**).

The statistical analysis performed using the Kruskal-Wallis test demonstrated a significant relationship between study outcomes and IF (*p* < 0.001; η^2^ = 0.02, 95% CI [0.003, 0.038]). Pairwise comparisons using the Mann-Whitney test revealed statistical significance for the comparison between the “diagnosis” (IF: median 4.0, IQR 3.1–5.1) and “neurobiological correlates” (IF: median 4.5, IQR 3.6–5.9) categories (*p* < 0.001 after Bonferroni correction). Complete results are provided in **Supplementary Table 2** and **Supplementary Table 3** in the **Supplementary Material B**.

The Kruskal-Wallis test indicated a statistically significant association between recruited population and IF (*p* < 0.05; η^2^ = 0.002, 95% CI [–0.004, 0.01]), although the effect size was negligible. Subsequent pairwise comparisons using the Mann-Whitney test revealed no statistically significant differences. For further details, see **Supplementary Table 4** and **Supplementary Table 5** in the **Supplementary Material B**.

## 4. Discussion

This systematic review has demonstrated the growth in interest in AI over time. The number of articles has increased (with almost half of the studies having been published in the last three years), and the related IF has also increased, although not to a statistically significant level. Consequently, certain journals have taken the initiative to establish dedicated sections addressing this topic, as exemplified by The New England Journal of Medicine through the launch of its “Artificial Intelligence in Medicine” series [36]. The integration of AI in the medical and mental healthcare fields gave rise to a number of concerns and uncertainties, leading to the identification of potential challenges and scenarios that could emerge as a result of this innovation [37, 38]. The observed increase in journal IF should be interpreted cautiously, as it may primarily reflect the overall expansion of AI research within medicine and mental health rather than a genuine improvement in the quality of studies specifically focused on SSDs. Therefore, while IF can provide useful context regarding publication trends, it should not be regarded as a direct indicator of the relevance or scientific rigor of individual SSD-related studies.

The primary consideration is the degree of ‘maturity’ of this novel technology, i.e., the extent to which it can be regarded as ‘ready and safe’. The assessment of this parameter is conducted through the utilisation of a scale devised by NASA in the 1970s, designated as the TRL (Technology Readiness Level) scale. This scale employs a 9-level rating system to evaluate technological readiness [39]; up to the present, no methods derived from AI have been shown to successfully pass all levels. Consequently, they are considered suitable for experimental and academic fields, but not for use in clinical practice. In the contemporary era, the realisation of this objective continues to be a formidable challenge, primarily due to the inherent deficiencies in the quality of the data utilised to develop the various algorithms and their subsequent implementation in the actual world. The accuracy of an AI algorithm is contingent on the data utilised during its development. The utilisation of databases and/or EHRs that have not been created for this purpose can result in errors, shortcomings or simplifications, thereby diminishing the reliability of the AI itself. Given the considerable volume of data necessitated by such analyses, the utilisation of existing datasets is frequently adopted as a strategy. Errors may occur during the process of dataset shift, particularly in relation to the type of population that is taken into consideration: for instance, the majority of the studies analysed included subjects diagnosed with schizophrenia; however, some studies analysed SSDs without distinguishing between various disorders. This can compromise the reliability of the algorithm. It is imperative that the data is both numerous and heterogeneous, and must also be specific. A further consideration relevant to the data utilised pertains to privacy issues. It should be noted that certain studies employ sensitive data and/or monitor subjects’ daily movements and activities. Given their nature as digital data, these records are potentially vulnerable to cyber theft.

In order to collect data that is considered adequate, it is usually necessary to use instrumental methods that provide a large amount of information even with small recruitment samples. Conversely, if these instrumental methods are not available, it is necessary to use large samples of subjects. In the systematic review, instrumental methods were employed in 83.30% of the studies, thus confirming their usefulness. Neuroimaging is the most frequently used instrumental method because a single scan contains a vast quantity of pixels that have the potential to serve as individual pieces of input data. However, other methods are also equally valid, such as EEG and genetic analyses.

This review indicates a shift in focus towards the identification of novel diagnostic methods and the correlation of neurobiological alterations with psychopathological dimensions. Currently, in the absence of pathognomonic biological markers for the various psychiatric disorders, diagnosis is made according to arbitrary criteria that inevitably lend themselves to inherent subjectivity. Consequently, there has been an emergence of classification systems that seek to enhance objectivity by basing diagnoses on biological correlates. A notable example of this is the RDoC (Research Domain Criteria) system, which was developed in 2009 by the National Institute of Mental Health (NIMH) [40]. It is therefore evident that there is a necessity for a more objective diagnosis, as is evidenced by the development of new classification systems and the search for technological aids to support this. These technological aids are often not well known to clinicians and therefore sometimes generate an attitude of mistrust [37]. This attitude is also rooted in a paucity of adequate legislation; indeed, one of the major dilemmas yet to be resolved is the attribution of responsibility in the event of an AI error: that of the authorising psychiatrist, the accepting patient, the developers of the algorithm, the health system that implemented the service, or no one? Consequently, there is an imperative for the integration of AI knowledge and expertise within the clinical domain of mental health, whilst concomitantly fostering progress across associated disciplines, particularly those pertaining to ethics and legislation.

The distribution of IF was found to be constant only for the “instrumental techniques” variable. For the remaining variables, including study outcomes and recruited populations, statistically significant but small variations in IF distribution were observed. In contrast, although the Kruskal-Wallis test for “type of AI” reached statistical significance, the effect size was negligible and no pairwise comparison survived correction, indicating the absence of meaningful differences across AI categories. Post-hoc analyses for study outcomes confirmed that the “neurobiological correlates” category was associated with a significantly higher IF compared with the “diagnosis” category after Bonferroni correction. This finding suggests that research interest extends beyond the identification of a pathology toward a deeper examination of its underlying mechanisms. Subsequent post-hoc analyses on the recruited population yielded no significant correlations, thereby suggesting that SSD-related pathologies are now regarded as a continuum spectrum with similar levels of interest.

Furthermore, an emerging area in the application of AI to brain disorders is generative artificial intelligence (GAI), encompassing models that generate new data, such as synthetic neuroimaging or simulated clinical text, based on learned patterns from existing datasets. While most GAI approaches are built on machine learning or deep learning frameworks, they hold promise for augmenting training datasets, modeling rare conditions, and exploring hypothetical scenarios. The field is rapidly expanding, with increasing publications demonstrating innovative applications in neuroscience research [41], and future work is expected to further integrate these approaches to enhance predictive modeling, data augmentation, and translational applicability.

### Limitations

This review has several important limitations that should be acknowledged. First, restricting the included studies to those published in English may introduce a degree of publication bias; however, prior evidence suggests that this effect is generally minor [42, 43].

The heterogeneity of reported outcomes across studies further limited standardized comparisons and emphasized the descriptive nature of the review. Metrics such as sensitivity, specificity, and clinical utility were not consistently reported, precluding the adoption of a uniform framework for comparative analyses. Consequently, a descriptive rather than analytical approach was employed, which provides a broad overview of methodological trends but limits the ability to directly compare AI model performance and reduces the granularity of conclusions regarding clinical applicability.

Another limitation lies in the rigid categorization of AI algorithms into ML, DL, and NLP. While this classification enhances clarity and consistency, it may oversimplify hybrid or emerging approaches that span multiple categories, potentially underrepresenting more complex or multimodal AI methodologies.

The largely descriptive and bibliometric approach of the review also constrains inferential conclusions. Although some statistical tests were applied to compare variables, they do not support robust inferential claims, and readers should interpret these findings with caution. Additionally, some studies reported multiple outcomes or instrumental techniques, which were treated as separate observations. This may introduce a degree of non-independence among data points, potentially violating assumptions of non-parametric analyses; however, given the relatively low proportion of such cases, the overall impact is likely minimal. The use of journal IF as a proxy for study relevance represents a further limitation.

While IF is a widely recognized bibliometric indicator and provides a standardized measure of journal visibility and influence, it does not fully capture the quality, scientific rigor, or specific impact of individual studies. Therefore, IF should be interpreted cautiously, and complementary metrics may offer a more comprehensive assessment of research significance [44, 45].

Finally, most included studies lacked external validation using independent datasets, and prospective evaluations were generally absent. This limits the real-world generalizability and clinical applicability of the AI models [46, 47]. Moreover, the majority of studies focused exclusively on schizophrenia, leaving the applicability to other SSD subtypes uncertain. Given that algorithmic performance depends heavily on the characteristics of the training dataset, transferability across different SSD populations cannot be assumed.

It is also notable that the subcategory “ARMS” was not originally included, as it is not formally classified within SSDs. Terms such as “ARMS”, “CHR”, and “UHR” were absent from the search strategy, highlighting the need for future reviews to incorporate these groups to achieve a more comprehensive assessment.

## 5. Conclusions

The potential of AI to support clinicians in developing novel, biologically informed classification systems for SSDs is considerable. Realizing this potential, however, requires the implementation of several critical measures. Future research should prioritize the expansion of well-designed NLP studies to fully leverage rich clinical text and voice data, complementing the prevailing focus on machine learning approaches. The development of high-quality, multi-site, multi-ethnic, and high-resolution neuroimaging and clinical datasets is essential, ensuring variability across key demographic and clinical variables (e.g., ethnicity, gender, age) while maintaining data specificity and robust privacy protections, including de-identification and federated-learning strategies. Equally important is the deployment of increasingly efficient, safe, and cost-effective algorithms that require minimal clinician oversight. Rigorous external validation using independent cohorts is necessary to advance AI models along the translational research pipeline and enhance their clinical applicability. Concurrently, clinicians should be trained to accurately interpret AI-derived outputs, and public engagement initiatives are needed to communicate both the benefits and limitations of AI, emphasizing the continued central role of clinicians in diagnostic and therapeutic decision-making. Finally, the establishment of ethical and legislative frameworks is imperative to ensure trust, safety, and equitable access, thereby promoting the responsible integration of AI into clinical practice.

## Availability of Data and Materials

All titles of the studies included in the present review are reported in the **Supplementary Materials**.

## Supplementary Material

Supplementary material associated with this article can be found, in the online version, at https://doi.org/10.31083/AP45104.
