# Supplementary material for: Artificial Intelligence in Schizophrenia Spectrum Disorders: Current Use and Future Perspectives. A Systematic Mapping Literature Review
Source: Alpha Psychiatry. 2026 Apr 23;27(2):45104. doi: 10.31083/AP45104 (PMC13156059; doi:10.31083/AP45104)
Supplement: Supplementary file 1 [file 2757-8038-27-2-45104-s1.zip › Supplementary Material A and B.docx]

**Supplementary Material A – LIST OF INCLUDED STUDIES ORGANIZED BY YEAR (1997-2024)**

**1997 (1 reference)**

1. Wang J, Begleiter H, Porjesz B. Parallel principal component neural networks for classification of event-related potential waveforms. IEEE Trans Biomed Eng. 1997;44(7):561-75.

**1998 (1 reference)**

2. Cohen JD, Servan-Schreiber D, McClelland JL. An artificial neural network stimulating performance of normal subjects and schizophrenics on the Wisconsin card sorting test. Proc Natl Acad Sci U S A. 1998;95(9):4803.

**1999 (3 references)**

3. Campana A, Duci A, Gambini O, Scarone S. An artificial neural network that uses eye-tracking performance to identify patients with schizophrenia. Schizophr Bull. 1999;25(4):789-99.

4. Corson PW, Nopoulos P, Miller DD, Arndt S, Andreasen NC. Caudate size in first-episode neuroleptic-naive schizophrenic patients measured using an artificial neural network. Am J Psychiatry. 1999;156(6):922-4.

5. Braver TS, Barch DM, Cohen JD. Cognition and control in schizophrenia: A computational model of dopamine and prefrontal function. Biol Psychiatry. 1999;46(3):312-28.

**2000 (1 reference)**

6. Frank MJ, Loughry B, O\'Reilly RC. A computational model of information processing in the frontal cortex and basal ganglia. Cogn Affect Behav Neurosci. 2000;1(2):137-60.

**2001 (2 references)**

7. Micheloyannis S, Pacheco E, Pachou E, Stam CJ, Vourkas M. Neural network analysis of the pattern of functional connectivity between cerebral areas in schizophrenia. Schizophr Res. 2001;49(1-2):141-2.

8. Hagg S, Spigset O, Bate R, Soderstrom E. Myocarditis related to clozapine treatment. J Clin Psychopharmacol. 2001;21(4):382-8.

**2002 (1 reference)**

9. Pierson R, Andreasen NC, O\'Leary DS, et al. Manual and semiautomated measurement of cerebellar subregions on MR images. Neuroimage. 2002;17(2):61-76.

**2004 (2 references)**

10. Pompili M, Mancinelli I, Girardi P, Ruberto A, Tatarelli R. Suicide risk factors and suicide vulnerability in various major psychiatric disorders. Expert Rev Neurother. 2004;4(4):627-38.

11. Gueorguieva R, Krystal JH. Methods for predictor analysis of repeated measurements: Application to psychiatric data. Stat Methods Med Res. 2004;13(6):481-93.

**2005 (1 reference)**

12. Hoffman RE, McGlashan TH. Elman topology with sigma-pi units: an application to the modeling of verbal hallucinations in schizophrenia. Pharmacopsychiatry. 2005;39 Suppl 1:S54-64.

**2006 (3 references)**

13. Calhoun VD, Adali T, Giuliani NR, Pekar JJ, Kiehl KA, Pearlson GD. A method for multitask fMRI data fusion applied to schizophrenia. Hum Brain Mapp. 2006;27(7):598-610.

14. Georgopoulos AP, Karageorgiou E, Leuthold AC, et al. Classification of functional brain images with a spatio-temporal dissimilarity map. Proc Natl Acad Sci U S A. 2006;104(35):14167-9.

15. Sabeti M, Katebi SD, Boostani R. Functional classification of schizophrenia using feed forward neural networks. Conf Proc IEEE Eng Med Biol Soc. 2006;1:6197-200.

**2008 (9 references)**

16. Rimol LM, Nesvåg R, Hagler DJ Jr, et al. Investigating possible subtypes of schizophrenia patients and controls based on brain cortical thickness. Schizophr Res. 2008;102(1-3):269.

17. Roke Y, van Harten PN, Boot AM, Buitelaar JK. Easy and low-cost identification of metabolic syndrome in patients treated with second-generation antipsychotics: artificial neural network and logistic regression models. J Clin Psychiatry. 2008;69(9):1442-50.

18. Modarresi A, Ghasem-Aghaee N, Tabesh H. Performance of a neuro-fuzzy model in predicting weight changes of chronic schizophrenic patients exposed to antipsychotics. Neuro Endocrinol Lett. 2008;29(6):955-62.

19. Demirci O, Clark VP, Magnotta VA, et al. Hybrid ICA-Bayesian network approach reveals distinct effective connectivity differences in schizophrenia. Neuroimage. 2008;46(4):1078-87.

20. Le-Niculescu H, Kurian SM, Yehyawi N, et al. Combining gene expression, demographic and clinical data in modeling disease: A case study of bipolar disorder and schizophrenia. Am J Med Genet B Neuropsychiatr Genet. 2008;150B(1):155-81.

21. Castro E, Martínez-Ramón M, Pearlson G, Sui J, Calhoun VD. Automatic relevance determination for identifying thalamic regions implicated in schizophrenia. J Neurosci Methods. 2008;174(1):103-9.

22. Burdick KE, Goldberg JF, Harrow M, et al. Analysis of cognitive performance in schizophrenia patients and healthy individuals with unsupervised clustering models. Neuropsychology. 2008;20(1):110.

23. Ince NF, Pellizzer G, Tewfik AH, Stephane M. Selection of spectro-temporal patterns in multichannel MEG with support vector machines for schizophrenia classification. Conf Proc IEEE Eng Med Biol Soc. 2008;2008:5421-4.

24. Lin E, Chen PS, Chang HP, et al. Artificial neural network prediction of clozapine response with combined pharmacogenetic and clinical data. J Clin Psychopharmacol. 2008;28(6):674-8.

**2009 (7 references)**

25. Xu L, Groth KM, Pearlson G, Schretlen DJ, Calhoun VD. Joint source based morphometry identifies linked gray and white matter group differences. Neuroimage. 2009;44(3):820-8.

26. Shen H, Wang L, Liu Y, Hu D. Discriminative analysis of resting-state functional connectivity patterns of schizophrenia using low dimensional embedding of fMRI. Neuroimage. 2009;49(4):3110-21.

27. Jafri MJ, Pearlson GD, Stevens M, Calhoun VD. Investigation of relationships between fMRI brain networks in the spectral domain using ICA and Granger causality reveals distinct differences between schizophrenia patients and healthy controls. Neuroimage. 2009;39(4):1666-81.

28. Cohen AS, Elvevåg B. Understanding anhedonia in schizophrenia through lexical analysis of natural speech. Schizophr Res. 2009;110(1):155.

29. Takahashi S, Cui Y, Robb MA, et al. Diagnostic classification of schizophrenia by neural network analysis of blood-based gene expression signatures. Schizophr Res. 2009;119(1-3):1-9.

30. Sun J, Zhao Z. Gene- and evidence-based candidate gene selection for schizophrenia and gene feature analysis. Bioinformatics. 2009;25(24):3218-21.

31. Kawasaki Y, Suzuki M, Kherif F, et al. Elucidating a magnetic resonance imaging-based neuroanatomic biomarker for psychosis: classification analysis using probabilistic brain atlas and machine learning algorithms. Biol Psychiatry. 2009;66(11):1090-2.

**2010 (8 references)**

32. Jagannathan V, Calhoun VD, Gelernter J, et al. A pilot multivariate parallel ICA study to investigate differential linkage between neural networks and genetic profiles in schizophrenia. Neuroimage. 2010;53(3):1070-8.

33. Takahashi S, Cui Y, Robb MA, et al. Diagnostic classification of schizophrenia by neural network analysis of blood-based gene expression signatures. Schizophr Res. 2010;119(1-3):1-9.

34. Anderson A, Cohen MS. Automatic bayesian classification of healthy controls, bipolar disorder, and schizophrenia using intrinsic connectivity maps from fMRI data. IEEE Trans Biomed Eng. 2010;57:1523.

35. Escartí MJ, de la Iglesia-Vayá M, Martí-Bonmatí L, et al. Increased amygdala and parahippocampal gyrus activation in schizophrenic patients with auditory hallucinations: An fMRI study using independent component analysis. Schizophr Res. 2010;117(1):31-41.

36. Khodayari-Rostamabad A, Reilly JP, Hasey GM, de Bruin H, Maccrimmon DJ. A pilot study to determine whether machine learning methodologies using pre-treatment electroencephalography can predict the symptomatic response to clozapine therapy. Clin Neurophysiol. 2010;121(12):1998-2006.

37. Yang H, Liu J, Sui J, Pearlson G, Calhoun VD. A Hybrid Machine Learning Method for Fusing fMRI and Genetic Data: Combining both Improves Classification of Schizophrenia. Front Hum Neurosci. 2010;4:192.

38. Ardekani BA, Nierenberg J, Hoptman MJ, Javitt DC, Lim KO. Neural classifiers for schizophrenia diagnostic support on diffusion imaging data. J Psychiatr Res. 2010;29(2):225-30.

39. Kaneda Y. Predictive factors of social functioning in patients with schizophrenia: Exploration for the best combination of variables using data mining. Psychiatry Clin Neurosci. 2010;64(5):560-3.

**2011 (5 references)**

40. Neuhaus AH, Popescu FC, Grozea C, et al. Single-subject classification of schizophrenia by event-related potentials during selective attention. Neuroimage. 2011;55(2):514-21.

41. Costafreda SG, Fu CH, Picchioni M, et al. Pattern of neural responses to verbal fluency shows diagnostic specificity for schizophrenia and bipolar disorder. BMC Psychiatry. 2011;11:18.

42. Hoffman RE, McGlashan TH. Using computational patients to evaluate Illness mechanisms in schizophrenia. Pharmacopsychiatry. 2011;44(S01):S54-64.

43. Castro E, Gómez-Verdejo MJ, Martínez-Ramón M, Kiehl KA, Calhoun VD. Characterization of groups using composite kernels and multi-source fMRI analysis data: Application to schizophrenia. Neuroimage. 2011;58(2):526-36.

44. Miyazato H, Hokama H, Touyama M. A neural network model of normal and abnormal auditory information processing. Psychiatry Clin Neurosci. 2011;65(3):289-91.

**2012 (6 references)**

45. Das P, Calhoun V, Malhi GS. Mentalizing in male schizophrenia patients is compromised by virtue of dysfunctional connectivity between task-positive and task-negative networks. Schizophr Res. 2012;140(1-3):51-8.

46. Plis SM, Weisend MP, Damaraju E, et al. Statistical epistasis and progressive brain change in schizophrenia: An approach for examining the relationships between multiple genes. Neuroimage. 2012;60(1):65-8.

47. Çetin MS, Christensen F, Abbott CC, et al. Modulations of functional connectivity in the healthy and schizophrenia groups during task and rest. Neuroimage. 2012;61:S1.

48. Neuhaus AH, Popescu FC, Rentzsch J, et al. Critical evaluation of auditory event-related potential deficits in schizophrenia: evidence from large-scale single-subject pattern classification. Schizophr Bull. 2012;40(5):1062-71.

49. Suh JY, Ehrlich S, Eyler L, et al. Multimodal analyses identify linked functional and white matter abnormalities within the working memory network in schizophrenia. Schizophr Res. 2012;136:S18.

50. Liu Y, Wang M, Wei X, et al. Prediction of serotonin transporter promoter polymorphism genotypes from single nucleotide polymorphism arrays using machine learning methods. Psychiatr Genet. 2012;22(6):269-74.

**2013 (9 references)**

51. Orliac F, Naveau M, Joliot M, Delcroix N, Mazoyer B, Etard O, et al. Links among resting-state default-mode network, salience network, and symptomatology in schizophrenia. Schizophr Res. 2013;148(1-3):74-80.

52. Holt DJ, Coombs G, Zeidan MA, Goff DC, Kaplowitz M, Cassotti G, et al. Normal brain activation in schizophrenia patients during associative emotional learning. Schizophr Res. 2013;145(1-3):40-7.

53. Xu M, Sun X, Zhang J, Li B, Xu J, Meng S, et al. Multidimensional analysis of the abnormal neural oscillations associated with lexical processing in schizophrenia. Schizophr Res. 2013;150(2-3):481-8.

54. Chang J, Jui-Heng C, Shyan-Ming Y. Applied computational techniques on schizophrenia using genetic mutations. Gene. 2013;518(1):151-8.

55. Nieuwenhuis M, van Haren NE, Hulshoff Pol HE, Cahn W, Kahn RS, Schnack HG. Brain morphometry of MR images for automated classification of first-episode schizophrenia. Neuroimage. 2013;64:441-51.

56. Koutsouleris N, Borgwardt S, Meisenzahl EM, Kabisch EM, Schmidt A, Maurice D, et al. Using genetic, cognitive and multi-modal neuroimaging data to identify ultra-high-risk and first-episode psychosis at the individual level. Schizophr Bull. 2013;39(1):14-7.

57. Nejad AB, Rotarska-Jagiela A, van de Ven V, Formisano E, Galderisi S, Dell\'Acqua F, et al. Neural markers of negative symptom outcomes in distributed working memory brain activity of antipsychotic-naive schizophrenia patients. Brain Imaging Behav. 2013;7(3):328-36.

58. Koutsouleris N, Meisenzahl EM, Borgwardt S, Riecher-Rössler A, Gaser C. Distinguishing prodromal from first-episode psychosis using neuroanatomical single-subject pattern recognition. Schizophr Bull. 2013;39(1):165-75.

59. Zhao G, Zhang J, Qin W, Deng Z, Qu M, Jiang T. Automatic statistical shape analysis of cerebral asymmetry in 3D T1-weighted magnetic resonance images at vertex-level: Application to neuroleptic-naïve schizophrenia. Med Image Anal. 2013;17(3):305-22.

**2014 (13 references)**

60. Dwyer DB, Harrison BJ, Yücel M, Pantelis C, Zalesky A, Fornito A. Multivariate neuroanatomical classification of cognitive subtypes in schizophrenia: a support vector machine learning approach. Neuroimage. 2014;102:139-48.

61. Sun D, van Erp TG, Thompson PM, Bearden CE, Cannon TD. Neurocognitive pattern analysis reveals classificatory hierarchy of attention deficits in Schizophrenia. Schizophr Bull. 2014;40(1):175-85.

62. Castro E, Anderson JM, Chen J, Calhoun VD. A multiple kernel learning approach to perform classification of groups from complex-valued fMRI data analysis: Application to schizophrenia. Proc IEEE Int Symp Biomed Imaging. 2014;2014:1003-6.

63. Schnack HG, Nieuwenhuis M, van Haren NE, Abramovic L, Scheewe TW, Brouwer RM, et al. Can structural MRI aid in clinical classification? A machine learning study in two independent samples of patients with schizophrenia, bipolar disorder and healthy subjects. Neuroimage. 2014;84:299-306.

64. Ma S, Miller RL, Du Y, Calhoun VD. Dynamic changes of spatial functional network connectivity in healthy individuals and schizophrenia patients using independent vector analysis. IEEE Trans Biomed Eng. 2014;61(12):2850-61.

65. Ebisch SJ, Mantini D, Northoff G, Salone A, De Berardis D, Ferri F, et al. Key functional circuitry altered in schizophrenia involves parietal regions associated with sense of self. Schizophr Bull. 2014;40(3):631-40.

66. Le M, Besteher B, Gaser C, Nenadic I. Latent semantic variables are associated with formal thought disorder and adaptive behavior in older inpatients with schizophrenia. Psychiatry Res. 2014;221(1):50-6.

67. Du W, Calhoun VD, Li H, Ma S, Eichele T, Kiehl KA, et al. Machine learning fMRI classifier delineates subgroups of schizophrenia patients. Phys Med Biol. 2014;57(9):2409-24.

68. Gireesan K, Sengottuvel S, Janawadkar MP, Venkatesh L, Arumugam K, Parasuraman R, et al. A machine learning approach using auditory odd-ball responses to investigate the effect of Clozapine therapy. Annu Int Conf IEEE Eng Med Biol Soc. 2014;2014:4811-4.

69. Neuhaus AH, Popescu FC, Rentzsch J, Gallinat J. Single-subject classification of schizophrenia patients based on a combination of oddball and mismatch evoked potential paradigms. Schizophr Res. 2014;153(1-3):141-6.

70. Pettersson-Yeo W, Benetti S, Marquand AF, Dell\'Acqua F, Williams SC, Allen P, et al. An empirical comparison of different approaches for combining multimodal neuroimaging data with support vector machine. Front Psychol. 2014;5:189.

71. Cabral C, Kherif F, Ritpiromchai S, Westman E, Simmons A, Meisenzahl E, et al. Classification of first-episode psychosis: a multi-modal multi-feature approach integrating structural and diffusion imaging. Neuroimage Clin. 2014;6:117-25.

72. Castellani U, Rossato E, Murino V, Bellani M, Rambaldelli G, Perlini C, et al. Wavelet features for recognition of first episode of schizophrenia from MRI brain images. Proc Int Conf Image Anal Process. 2014;1:215-22.

**2015 (16 references)**

73. Suwa A, Matsuo K, Hiramoto T, Kato A, Hashizume C, Sato S, et al. Impaired target detection in schizophrenia and the ventral attentional network: Findings from a joint event-related potential-functional MRI analysis: Target stimulus ERP/fMRI analysis in schizophrenia. Psychiatry Clin Neurosci. 2015;69(1):16-26.

74. Chahine G, Richter A, Wolter S, Goya-Maldonado R, Gruber O. Discrimination of schizophrenia auditory hallucinators by machine learning of resting-state functional MRI. Front Psychiatry. 2015;6:154.

75. Jiang L, Tian L, Calhoun VD. Task-based functional connectivity as an indicator of genetic liability to schizophrenia. Schizophr Bull. 2015;41(1):218-28.

76. Broome MR, Saunders KE, Harrison PJ, Marwaha S. Mood instability is a common feature of mental health disorders and is associated with poor clinical outcomes. BMJ Case Rep. 2015;2015:bcr2015211516.

77. Vigod SN, Seeman MV, Ray JG, Anderson GM, Dennis CL, Grigoriadis S, et al. The characteristics and health needs of pregnant women with schizophrenia compared with bipolar disorder and affective psychoses. BMC Med. 2015;13:205.

78. He H, Sui J, Yu Q, Chen J, Qi S, Du Y, et al. The fault lies on the other side: Altered brain functional connectivity in psychiatric disorders is mainly caused by counterpart regions in the opposite hemisphere. Cereb Cortex. 2015;25(9):2875-86.

79. Wang X, Cheng H, Qin W, Li J, Zhao L, Yu C, et al. Edge-Centered DTI Connectivity Analysis: Application to Schizophrenia. PLoS One. 2015;10(6):e0130654.

80. Liu F, Zhuo C, Yu C. Nodal centrality of functional network in the differentiation of schizophrenia. Sci Rep. 2015;5:16533.

81. Kaufmann T, Skåtun KC, Alnæs D, Doan NT, Duff EP, Tønnesen S, et al. Disintegration of Sensorimotor Brain Networks in Schizophrenia. Schizophr Bull. 2015;41(6):1326-35.

82. Qureshi MN, Oh J, Lee B. Computer aided diagnosis of schizophrenia on resting state fMRI data by ensembles of ELM. Front Neurosci. 2015;11:413.

83. Wu P, Moon J, Jeng-Hsiu H, He J. Extracting antipsychotic polypharmacy data from electronic health records: Developing and evaluating a novel process. BMC Med Inform Decis Mak. 2015;15:104.

84. Al-Kadi ID, Al-Tuwaijari B, Soliman NF, Al-Rawi M, El-Samie FEA. Handling diagnosis of schizophrenia by a hybrid method. J Med Syst. 2015;39(3):32.

85. Mucci A, Galderisi S, Gibertoni D, Rossi A, Rocca P, Bertolino A, et al. Negative symptoms in schizophrenia: A study in a large clinical sample of patients using a novel automated method. Eur Psychiatry. 2015;30(1):65-72.

86. Qureshi MN, Oh J, Cho D, Knickmeyer RC, Davis S, Lee B. Local activity features for computer aided diagnosis of schizophrenia on resting-state fMRI. J Med Syst. 2015;39(12):189.

87. Patel R, Wilson R, Jackson R, Ball M, Shetty H, Broadbent M, et al. Cannabis use and treatment resistance in first episode psychosis: a natural language processing study. Lancet Psychiatry. 2015;2(12):1075-83.

88. Pisharady PK, Sastry PS, Kumaraswamy S, Ganapathy S, Sahu A, Kumar P, et al. The use of dynamic susceptibility contrast (DSC) MRI to automatically classify patients with first episode psychosis. Brain Informatics. 2015;2(3):141-52.

**2016 (11 references)**

89. Brookes MJ, Tewarie PK, Hunt BA, Robson SE, Gascoyne LE, Liddle EB, et al. A multi-layer network approach to MEG connectivity analysis. Neuroimage. 2016;132:425-38.

90. Du Y, Pearlson GD, Lin D, Sui J, Chen J, Salman M, et al. Interaction among subsystems within default mode network diminished in schizophrenia patients: A dynamic connectivity approach. Schizophr Res. 2016;170(1):55-65.

91. Anticevic A, Repovs G, Corlett PR, Barch DM. Reduced load-dependent default mode network deactivation across executive tasks in schizophrenia spectrum disorders. J Abnorm Psychol. 2016;125(2):224-37.

92. Curcic-Blake B, van de Ven V, Knegtering H, Eniola L, Liemburg EJ, Bruggeman R, et al. Dynamic Causal Modeling and machine learning for effective connectivity in Auditory Hallucination. Neuroimage Clin. 2016;12:12-21.

93. Strauss GP, Waltz JA, Gold JM. Probabilistic Reinforcement Learning in Patients With Schizophrenia: Relationships to Anhedonia and Avolition. Biol Psychiatry Cogn Neurosci Neuroimaging. 2016;1(1):61-9.

94. Kim J, Calhoun VD, Shim E, Lee JH. Deep neural network with weight sparsity control and pre-training extracts hierarchical features and enhances classification performance: Evidence from whole-brain resting-state functional connectivity patterns of schizophrenia. Neuroimage. 2016;124(Pt A):127-46.

95. Plis SM, Amin MF, Haque A, Damaraju E, Miller R, Abbott PD, et al. Using deep belief network modelling to characterize differences in brain morphometry in schizophrenia. Sci Rep. 2016;6:19538.

96. Hjelm RD, Calhoun VD, Salakhutdinov R, Plis SM. Deep Independence Network Analysis of Structural Brain Imaging: Application to Schizophrenia. IEEE Trans Med Imaging. 2016;35(7):1742-52.

97. Shim M, Hwang HJ, Kim DW, Lee SH, Im CH. Machine-learning-based diagnosis of schizophrenia using combined sensor-level and source-level EEG features. Schizophr Res. 2016;176(2-3):314-9.

98. Mikolas P, Melicher T, Skoch A, Jajcay L, Nemec H, Bakstein E, et al. Connectivity of the anterior insula differentiates participants with first-episode schizophrenia spectrum disorders from controls: A machine-learning study. Psychol Med. 2016;46(13):2695-704.

99. Alderson-Day B, Diederen K, Agnew Z, Evans S, Duggan A, Scott S, et al. Individualized covariance profile of cortical morphology for auditory hallucinations in first-episode psychosis. Schizophr Bull. 2016;42(1):152-60.

**2017 (35 references)**

100. Moran LV, Masters GA, Boyd JD, Steger RA, Ongur D, Evins AE, et al. Nicotinic modulation of salience network connectivity and centrality in schizophrenia. Biol Psychiatry Cogn Neurosci Neuroimaging. 2017;2(7):599-606.

101. He H, Yang S, Zhao J, Guo Z, Zhao L, Wang J, et al. Music Intervention Leads to Increased Insular Connectivity and Improved Clinical Symptoms in Schizophrenia. Front Neurosci. 2017;11:744.

102. Oh J, Bakstein E, Martinec N, Jarolimek M, Amanowicz M, Lee B. Computer-aided diagnostics of schizophrenia: Comparison of different feature extraction methods. IEEE Access. 2017;5:16124-41.

103. Jääskeläinen IP, Pajula J, Nieminen J, Saramäki J, Glerean E, Tikka P, et al. Precuneus functioning differentiates first-episode psychosis patients during the fantasy movie Alice in Wonderland. Sci Rep. 2017;7:46153.

104. Kim J, Wüstenberg T, Belger A, Diekhof EK, Goya-Maldonado R, Richter A, et al. On the integrity of functional brain networks in schizophrenia, Parkinson\'s disease, and advanced age: Evidence from connectivity-based single-subject classification. Hum Brain Mapp. 2017;38(6):2990-3007.

105. Ma Y, Chen J, Ding Y, Wu S, Zhang J, Cui J, et al. Comparative study on serum levels of macro and trace elements in schizophrenia based on supervised learning methods. J Trace Elem Med Biol. 2017;44:114-21.

106. Nieuwenhuis M, van Haren NE, Hulshoff Pol HE, Cahn W, Kahn RS, Schnack HG. Multi-center MRI prediction models: Predicting sex and illness course in first episode psychosis patients. Neuroimage. 2017;145(Pt B):246-53.

107. Giner L, Jáuregui J, Barrigón ML, Molina-López A, de la Vega D, Currier D, et al. Classification of suicide attempters in schizophrenia using sociocultural and clinical features: A machine learning approach. J Psychiatr Res. 2017;84:33-9.

108. Patel R, Jayatilleke N, Broadbent M, Rashid T, Shetty H, Hayhurst H, et al. Identification of the delivery of cognitive behavioural therapy for psychosis using a cross-sectional sample from electronic health records and open-text information in a large UK-based mental health case register. BMJ Open. 2017;7(3):e014445.

109. Pradeep J, Srinivasan K. An efficient FMRI classification technique in cloud using multiple parallel feature selection algorithm. Cluster Comput. 2017;20(2):1043-52.

110. Zhang J, Harvey PD, Ma C, Ba P, Sun Y, Li C, et al. Predicting future high-cost schizophrenia patients using High-Dimensional administrative data. Psychiatry Res. 2017;252:176-82.

111. Matsubara T, Campello RJGB, Sato JR, Ushio T. Kernel alignment for identifying objective criteria from brain MEG recordings in schizophrenia. IEEE Trans Biomed Eng. 2017;64(4):753-64.

112. Koutsouleris N, Davatzikos C, Borgwardt S, Gaser C, Bottlender R, Frodl T, et al. BrainAGE score indicates accelerated brain aging in schizophrenia, but not bipolar disorder. Biol Psychiatry. 2017;82(6):413-25.

113. Bethlehem RAI, Seidlitz J, Romero-Garcia R, Yeh PH, Stocker AM, Vértes PE, et al. Brain-Behavior Participant Similarity Networks among Youth and Emerging Adults with Schizophrenia Spectrum, Autism Spectrum, or Bipolar Disorder and Matched Controls. JAMA Netw Open. 2017;3(3):e200344.

114. Lefebvre S, Demeulemeester M, Leroy A, Delmaire C, Lopes R, Pins D, et al. Resting State Effective Connectivity Allows Auditory Hallucination Discrimination. Front Psychiatry. 2017;7:210.

115. Bain EE, Shafner L, Walling DP, Othman AA, Chuang-Stein C, Hanzelka A, et al. Use of a novel artificial intelligence platform on mobile devices to assess dosing compliance in a phase 2 clinical trial in subjects with schizophrenia. Psychopharmacology (Berl). 2017;234(10):1561-7.

116. Zhang Y, He G, Wang Z, Feng R, Feng J. Distinct multivariate brain morphological patterns and their added predictive value with cognitive and polygenic risk scores in mental disorders. Cereb Cortex. 2017;27(9):4308-21.

117. Kim JW, Lee YS, Han DH, Min KJ, Lee J, Lee K. A computer-aided diagnosis system with EEG based on the p3b wave during an auditory odd-ball task in schizophrenia. Ann Gen Psychiatry. 2017;16:42.

118. Wang J, Zhao L, Guo Z, He H, Zhang X, Nie S, et al. Convergence and divergence of neurocognitive patterns in schizophrenia and depression. Psychiatry Res Neuroimaging. 2017;267:51-8.

119. Jiao Z, Huang S, Zhu J, Savio AM, Cao P, Liu M, et al. Peripheral biomarker signatures of bipolar disorder and schizophrenia: A machine learning approach. J Affect Disord. 2017;209:169-77.

120. Pineda-Pardo JA, Gomez-Verdejo V, Castellanos FX, Martinez-Cantarin J, Martinez-García N. Non redundant functional brain connectivity in schizophrenia. Front Hum Neurosci. 2017;11:215.

121. Shao Y, Chen J, Pinaya WHL, Mechelli A, Calhoun VD, Sato JR. Transdiagnostic differences in the resting-state functional connectivity of the prefrontal cortex in depression and schizophrenia. J Affect Disord. 2017;221:288-94.

122. Huang S, Li Y, Zhang Z, He H, Nie S, Zhang X, et al. Classifying Schizotypy Using an Audiovisual Emotion Perception Test and Scalp Electroencephalography. Front Hum Neurosci. 2017;11:151.

123. Tang Y, Hu X, Wang J, Xu J, Zhuo K, Qian Z, et al. Abnormal neural activity as a potential biomarker for drug-naive first-episode adolescent-onset schizophrenia with coherence regional homogeneity and support vector machine analyses. Sci Rep. 2017;7(1):153.

124. Cheng J, Guiraud L, Shao Y, Pinaya WH, Sato JR, Mechelli A. Multimodal Discrimination of Schizophrenia Using Hybrid Weighted Feature Concatenation of Brain Functional Connectivity and Anatomical Features with an Extreme Learning Machine. Procedia Comput Sci. 2017;110:444-51.

125. Thangaraj P, Srinivasan K. fMRI based computer aided diagnosis of schizophrenia using fuzzy kernel feature extraction and hybrid feature selection. J Med Imaging Health Inform. 2017;7(1):171-82.

126. Yan W, Calhoun V, Pan M, Lin D, Sui J, Chen J, et al. Evaluation of machine learning algorithms and structural features for optimal MRI-based diagnostic prediction in psychosis. Med Image Anal. 2017;35:599-612.

127. Kim J, Calhoun VD, Shim E, Lee JH. A robust sparse-modeling framework for estimating schizophrenia biomarkers from fMRI. IEEE Trans Biomed Eng. 2017;64(11):2613-29.

128. Sadeghi M, Khosrowabadi R, Bakouie F, Mahaki H, Javan M, Pouretemad HR. Network modeling of resting state connectivity points towards the bottom up theories of schizophrenia. Front Comput Neurosci. 2017;11:51.

129. Kushibar K, Salem M, Valentin A, Oliver A, Lladó X, Martí R. Deep neural networks for segmentation of basal ganglia sub-structures in brain MR images. Proc Int Conf Med Image Comput Comput Assist Interv. 2017;10435:102-10.

130. Vieira S, Pinaya WHL, Mechelli A. Diagnosing Schizophrenia: A Deep Learning Approach. In: Machine Learning. Academic Press; 2017. p. 237-54.

131. Huang H, Hu X, Zhao J, Guo Z, Zhao L, Wang J, et al. Differences Between Schizophrenic and Normal Subjects Using Network Properties from fMRI. Front Hum Neurosci. 2017;11:53.

132. Cabral C, Kherif F, Ritpiromchai S, Westman E, Simmons A, Meisenzahl E, et al. Classification of first-episode psychosis in a large cohort of patients using support vector machine and multiple kernel learning techniques. Neuroimage Clin. 2017;14:383-91.

133. Dwyer DB, Cabral C, Kherif F, Faltraco F, Linden DE, Kahn RS, et al. Multi-center machine learning in imaging psychiatry: A meta-model approach. Neuroimage. 2017;145(Pt B):232-45.

134. Jensen MH, Larsson AL, Hjorthøj C, Nordentoft M, Fagerlund B, Glenthøj B, et al. Two subgroups of antipsychotic-naive, first-episode schizophrenia patients identified with a Gaussian mixture model on cognition and electrophysiology. Schizophr Res. 2017;188:142-8.

**2018 (45 references)**

135. Shovon MH, Islam MA, Alam MA, Kabir MA. Schizophrenic patient identification using graph-theoretic features of resting-state fMRI data. Proc Int Conf Bioinform Comput Biol. 2018;2018:145-52.

136. Kolenic M, Franke K, Hlinka J, Matejka M, Capkova J, Pašková B, et al. Obesity, dyslipidemia and brain age in first-episode psychosis. J Psychiatr Res. 2018;99:112-20.

137. Watanabe T, Hirose S, Wada H, Imai Y, Machiyama T, Tanaka S, et al. Prediction of activation patterns preceding hallucinations in patients with schizophrenia using machine learning with structured sparsity. Biol Psychiatry. 2018;83(5):383-92.

138. Kanchanatawan B, Sirivichayakul S, Thika S, Carvalho AF, Geffard M, Maes M. In Schizophrenia, Depression, Anxiety, and Physiosomatic Symptoms Are Strongly Related to Psychotic Symptoms and Excitation, Impairments in Episodic Memory, and Increased Production of Neurotoxic Tryptophan Catabolites: a Multivariate and Machine Learning Study. Neurotox Res. 2018;33(3):641-55.

139. Maes M, Sirivichayakul S, Kanchanatawan B, Geffard M, Carvalho AF, Roomruangwong C, et al. Deficit schizophrenia is a discrete diagnostic category defined by neuro-immune and neurocognitive features: results of supervised machine learning. Mol Neurobiol. 2018;55(10):8161-75.

140. Wolfers T, Arenas AL, Zeeuw JD, Onnink M, Dammers J, Hoogman M, et al. Reproducible grey matter patterns index a multivariate, global alteration of brain structure in schizophrenia and bipolar disorder. Neuroimage Clin. 2018;20:1128-36.

141. Waters F, Fernyhough C. Draining the pond and catching the fish: Uncovering the ecosystem of auditory verbal hallucinations. Schizophr Bull. 2018;44(6):1174-6.

142. Su L, Cai Y, Xu Y, Dutt A, Shi S, Bramon E. Effective connectivity within a triple network brain system discriminates schizophrenia spectrum disorders from psychotic bipolar disorder at the single-subject level. Schizophr Res. 2018;199:210-21.

143. Patel R, Reiss P, Shetty H, Broadbent M, Stewart R, McGuire P, et al. Recorded atypical hallucinations in psychotic and affective disorders and associations with non-benzodiazepine hypnotic use: The South London and Maudsley Case Register. J Psychopharmacol. 2018;32(11):1199-205.

144. Sui J, Qi S, Van Erp TG, Calhoun VD. Multimodal neuromarkers in schizophrenia via cognition-guided MRI fusion. Nat Commun. 2018;9(1):3028.

145. Koutsouleris N, Hauser TU, Skvortsova V, De Choudhury M. Machine learning-guided intervention trials to predict treatment response at an individual patient level: an important second step following randomized clinical trials. JAMA Psychiatry. 2018;75(11):1113-4.

146. Qi S, Sui J, Chen J, Yan W, Calhoun VD. Multimodal Fusion with Reference: Searching for Joint Neuromarkers of Working Memory Deficits in Schizophrenia. IEEE Int Conf Acoust Speech Signal Process. 2018;2018:935-9.

147. Rozycki M, Satterthwaite TD, Koutsouleris N, Erus G, Doshi J, Wolf DH, et al. Multisite machine learning analysis provides a robust structural imaging signature of schizophrenia detectable across diverse patient populations and within individuals. Schizophr Bull. 2018;44(5):1035-44.

148. Cao B, Cho RY, Chen D, Xiu M, Tan Y, Zunta-Soyan M, et al. Predicting Response to Repetitive Transcranial Magnetic Stimulation in Patients With Schizophrenia Using Structural Magnetic Resonance Imaging: A Multisite Machine Learning Analysis. EBioMedicine. 2018;27:260-7.

149. Dwyer DB, Koutsouleris N. Multivariate pattern analysis of genotype-phenotype relationships in schizophrenia. Schizophr Bull. 2018;44(6):1187-8.

150. Wang J, Zhao L, Guo Z, He H, Zhang X, Nie S, et al. Multi-Site Diagnostic Classification of Schizophrenia Using Discriminant Deep Learning with Functional Connectivity MRI. EBioMedicine. 2018;30:74-85.

151. Chen J, Qi S, Calhoun VD, Du Y, Sui J. Brain Subtyping Enhances The Neuroanatomical Discrimination of Schizophrenia. Conf Proc IEEE Eng Med Biol Soc. 2018;2018:3890-3.

152. Salman MS, Du Y, Lin D, Chen J, Kiehl KA, Calhoun VD. Reading the (functional) writing on the (structural) wall: Multimodal fusion of brain structure and function via a deep neural network based translation approach reveals novel impairments in schizophrenia. IEEE Trans Biomed Eng. 2018;66(3):660-70.

153. Stochl J, Khandaker GM, Lewis G, Perez J, Goodyer IM, Zammit S, et al. Patterns of schizophrenia symptoms: hidden structure in the PANSS questionnaire. Mol Psychiatry. 2018;23(10):2090-7.

154. van Dellen E, Sommer IE, Bohlken MM, Tewarie P, Draaisma L, Zalesky A, et al. Disentangling age- and disease-related alterations in schizophrenia brain network using structural equation modeling: A graph theoretical study based on minimum spanning tree. Hum Brain Mapp. 2018;39(11):4415-27.

155. de la Fuente-Sandoval C, Favila R, Reyes-Madrigal F, Azcárate-Estrada M, León-Ortiz P. Discriminating schizophrenia and schizo-obsessive disorder: a structural MRI study combining VBM and machine learning methods. Schizophr Res. 2018;197:323-30.

156. Koutsouleris N, Kambeitz-Ilankovic L, Meisenzahl EM, Lalousis PA, Wood SJ, Antonucci LA, et al. Identifying a neuroanatomical signature of schizophrenia, reproducible across sites and stages, using machine learning with structured sparsity. Biol Psychiatry. 2018;83(3):196-208.

157. Kim J, Calhoun VD, Shim E, Lee JH. Multisite generalizability of schizophrenia diagnosis classification based on functional brain connectivity. IEEE Trans Med Imaging. 2018;37(5):1096-107.

158. Schnack HG, van Haren NE, Hulshoff Pol HE, Kahn RS. Is there a symptomatic distinction between the affective psychoses and schizophrenia? A machine learning approach. Schizophr Res. 2018;192:154-61.

159. Gradin VB, Waiter G, O\'Connor A, Douglas J, Steele JD, Kumar P, et al. Modeling subjective relevance in schizophrenia and its relation to aberrant salience. Hum Brain Mapp. 2018;39(5):2075-88.

160. Sahli S, Gaser C. Caudate volumes derived from a neural network: Comparison of neuroleptic naive patients with schizophrenia and matched control subjects. Psychiatry Res Neuroimaging. 2018;272:12-8.

161. Cao B, Cho RY, Chen D, Xiu M, Tan Y, Zunta-Soyan M, et al. A network-based classification framework for predicting treatment response of schizophrenia patients. EBioMedicine. 2018;27:260-7.

162. Dwyer DB, Falkai P, Koutsouleris N. Comparative evaluation of machine learning strategies for analyzing big data in psychiatry. Int J Methods Psychiatr Res. 2018;27(2):e1713.

163. Sun J, Wang J, He H, Zhao L, Guo Z, Li J, et al. Disease definition for schizophrenia by functional connectivity using radiomics strategy. Front Genom. 2018;12:124.

164. Lu X, Yang Y, Wu F, Liu J, Luan S, Zhang Y, et al. Recognition of Schizophrenia with Regularized Support Vector Machine and Sequential Region of Interest Selection using Structural Magnetic Resonance Imaging. Front Neurosci. 2018;12:479.

165. Mason L, Peters ER, Dazzan P, Kumari V, Williams SC, Antoniou G, et al. Using fMRI and machine learning to predict symptom improvement following cognitive behavioural therapy for psychosis. Psychol Med. 2018;48(11):1858-66.

166. Ramasubbu V, Ramalingam G. Detection of Schizophrenia in brain MR images based on segmented ventricle region and deep belief networks. J Med Syst. 2018;42(12):242.

167. Shao Y, Chen J, Pinaya WHL, Mechelli A, Calhoun VD, Sato JR. Anatomical connectivity changes in bipolar disorder and schizophrenia investigated using whole-brain tract-based spatial statistics and machine learning approaches. J Affect Disord. 2018;229:331-8.

168. Cheng J, Guiraud L, Shao Y, Pinaya WH, Sato JR, Mechelli A. Combination of G72 Genetic Variation and G72 Protein Level to Detect Schizophrenia: Machine Learning Approaches. Mol Neurobiol. 2018;55(11):8532-42.

169. Sumiyoshi C, Higuchi Y, Sumiyoshi T. Semantic memory organization in Japanese patients with schizophrenia examined with category fluency. Schizophr Res Cogn. 2018;12:28-34.

170. Castro E, Hjelm RD, Plis SM, Dyrba M, Calhoun VD. A kernel machine method for detecting higher order interactions in multimodal datasets: Application to schizophrenia. Proc IEEE Int Conf Acoust Speech Signal Process. 2018;2018:945-9.

171. Kishi T, Shinohara K, Matsunaga S, Matsuda Y, Nomura I, Itake K, et al. Multiscale network dynamics between heart rate and locomotor activity are altered in schizophrenia. Schizophr Res. 2018;192:132-8.

172. Fernandes PP, Linhares D, Reis J, Costa P. Continuous assessment of schizophrenia using heart rate and accelerometer data. IEEE J Biomed Health Inform. 2018;22(4):1123-30.

173. Maes M, Sirivichayakul S, Kanchanatawan B, Geffard M, Carvalho AF, Roomruangwong C, et al. Towards a new classification of stable phase schizophrenia into major and simple neuro-cognitive psychosis: Results of unsupervised machine learning analysis. J Eval Clin Pract. 2018;24(1):257-66.

174. Wang X, Li J, Zhao L, Zhao J, Guo Z, He H, et al. Magnetic resonance imaging study of gray matter in schizophrenia based on xgboost. Front Genom. 2018;12:135.

175. Carrion RE, Correll CU, Auther AM, Cornblatt BA. The Early Psychosis Screener (EPS): Quantitative validation against the SIPS using machine learning. Schizophr Res. 2018;192:249-54.

176. Mota NB, Weissheimer J, Ribeiro S, Copelli M. Neurocognitive Graphs of First-Episode Schizophrenia and Major Depression Based on Cognitive Features. Front Hum Neurosci. 2018;12:123.

177. Mikolas P, Melicher T, Skoch A, Jajcay L, Nemec H, Bakstein E, et al. Machine learning classification of first-episode schizophrenia spectrum disorders and controls using whole brain white matter fractional anisotropy. Schizophr Res. 2018;192:175-82.

178. Salvatore C, Cerasa A, Castiglioni I, Gilardi MC, Parati G, Perani D, et al. Investigating brain structural patterns in first episode psychosis and schizophrenia using MRI and a machine learning approach. Front Neurosci. 2018;12:94.

179. Bambini V, Martini G, Ricci I, Terai A, Shum J, Pantelis C, et al. Non literal language comprehension in a large sample of first episode psychosis patients in adulthood. Schizophr Res. 2018;199:131-7.

**2019 (56 references)**

180. Yan W, Calhoun VD, Sui J, Du Y, Chen J, Kiehl KA, et al. Discriminating schizophrenia using recurrent neural network applied on time courses of multi-site FMRI data. EBioMedicine. 2019;47:427-38.

181. Rezaii N, Walker E, Wolff P. A machine learning approach to predicting psychosis using semantic density and latent content analysis. NPJ Schizophr. 2019;5(1):9.

182. Hirjak D, Kubera KM, Wolf RC, Sambataro F. Patterns of co-altered brain structure and function underlying neurological soft signs in schizophrenia spectrum disorders. Hum Brain Mapp. 2019;40(10):3029-41.

183. Wu M, Yan H, Zhang Y, Gao S, Zhao L, Wang J, et al. Transdiagnostic Multimodal Neuroimaging in Psychosis: Structural, Resting-State, and Task Magnetic Resonance Imaging Correlates of Cognitive Control. Schizophr Bull. 2019;45(Suppl 2):S212.

184. Yan W, Sui J, Calhoun VD, Kiehl KA, Fu Z, Wang L, et al. Brain-based ranking of cognitive domains to predict schizophrenia. Cereb Cortex. 2019;29(7):3001-14.

185. Rolland B, Amad A, Pins D, Thomas P. Structure/function interrelationships in patients with schizophrenia who have persistent auditory verbal hallucinations: A multimodal MRI study using parallel ICA. Schizophr Res. 2019;208:247-54.

186. Corbière M, Villemure R, Lecomte T. Longitudinal trajectory of early functional recovery in patients with first episode psychosis. Psychiatry Res. 2019;272:512-9.

187. Maes M, Rachayon M, Sirivichayakul S, Kanchanatawan B. Supervised machine learning to decipher the complex associations between neuro-immune biomarkers and quality of life in schizophrenia. CNS Spectr. 2019;24(6):613-24.

188. Reticcioli M, Di Giacomo E, Alessi F, Clerici M, Carra G. Predicting mechanical restraint of psychiatric inpatients by applying machine learning on electronic health data. J Psychiatr Res. 2019;115:102-7.

189. Fusar-Poli P, Hijazi Z, Stahl D, Steyerberg EW. Clinical-learning versus machine-learning for transdiagnostic prediction of psychosis onset in individuals at-risk. Schizophr Res. 2019;211:42-8.

190. Thoma P, Friedmann C, Suchan B. Neural correlates of victimization in psychosis: differences in brain response to angry faces. Eur Arch Psychiatry Clin Neurosci. 2019;269(2):173-86.

191. Lançon C, Fond G, Boyer L, Auquier P, Schürhoff F, Llorca PM, et al. Machine learning for predicting psychotic relapse at 2 years in schizophrenia in the national FACE-SZ cohort. Prog Neuropsychopharmacol Biol Psychiatry. 2019;92:179-85.

192. Patel R, Wilson R, Shetty H, Jackson R, Broadbent M, Stewart R, et al. Use of Natural Language Processing to identify Obsessive Compulsive Symptoms in patients with schizophrenia, schizoaffective disorder or bipolar disorder. NPJ Schizophr. 2019;5(1):2.

193. Liddle EB, Gascoyne LE, Robson SE, Liddle PF. Relationship between MEG global dynamic functional network connectivity measures and symptoms in schizophrenia. Schizophr Bull. 2019;45(Suppl 2):S234.

194. Clementz BA, Sweeney JA, Hamm JP, Ivleva EI, Ethridge LE, Pearlson GD, et al. Machine learning improved classification of psychoses using clinical and biological stratification: Update from the bipolar-schizophrenia network for intermediate phenotypes (B-SNIP). Schizophr Bull. 2019;45(Suppl 2):S221.

195. Palmiere AL, Di Giacomo E, Carra G, Clerici M. Identifying psychosis spectrum disorder from experience sampling data using machine learning approaches. Psychiatry Res. 2019;273:661-8.

196. Kadra G, Stewart R, Shetty H, Broadbent M, Patel R, Roberts A, et al. Analysis of risk factor domains in psychosis patient health records. PLOS ONE. 2019;14(1):e0210034.

197. Cho RY, Tan Y, Xiu M, Tan S, Zunta-Soyan M, Cao B, et al. Relative importance of symptoms, cognition, and other multilevel variables for psychiatric disease classifications by machine learning. Schizophr Bull. 2019;45(Suppl 2):S190.

198. Fu Z, Tu Y, Di X, Du Y, Pearlson GD, Turner JA, et al. A framework for linking resting-state chronnectome/genome features in schizophrenia: A pilot study. IEEE Trans Med Imaging. 2019;38(4):940-50.

199. Passos IC, Mwangi B, Vieta E, Berk M, Kapczinski F. Machine Learning Models Identify Multimodal Measurements Highly Predictive of Transdiagnostic Symptom Severity for Mood, Anhedonia, and Anxiety. J Clin Psychiatry. 2019;80(1):17m11921.

200. Oh J, Bakstein E, Martinec N, Jarolimek M, Amanowicz M, Lee B. Classification of schizophrenia and normal controls using 3D convolutional neural network and outcome visualization. Schizophr Res. 2019;212:186-95.

201. Du Y, Fu Z, Sui J, Gao S, Cho RY, Pearlson GD, et al. Brain network dynamics in schizophrenia: Reduced dynamism of the default mode network. Cereb Cortex. 2019;29(11):4546-67.

202. Han C, Mansour LS, Baker ST, Tavor I, Caspers S, Knosche TR, et al. Deep Neural Generative Model of Functional MRI Images for Psychiatric Disorder Diagnosis. IEEE Trans Med Imaging. 2019;38(11):2516-25.

203. Wang J, Zhao L, Guo Z, He H, Zhang X, Nie S, et al. Machine learning identifies unaffected first-degree relatives with functional network patterns and cognitive impairment similar to those of schizophrenia patients. Schizophr Bull. 2019;45(Suppl 2):S274.

204. Pinaya WHL, Mechelli A, Rezende JRR, Sato JR. Using deep autoencoders to identify abnormal brain structural patterns in neuropsychiatric disorders: A large-scale multi-sample study. Hum Brain Mapp. 2019;40(5):1452-65.

205. Oh J, Bakstein E, Martinec N, Jarolimek M, Amanowicz M, Lee B. 3D-CNN based discrimination of schizophrenia using resting-state fMRI. Artif Intell Med. 2019;94:107-14.

206. Su L, Cai Y, Xu Y, Dutt A, Shi S, Bramon E. Low-rank network signatures in the triple network separate schizophrenia and major depressive disorder. NPJ Schizophr. 2019;5(1):15.

207. Zhang J, He G, Wang Z, Feng R, Feng J. Towards artificial intelligence in mental health by improving schizophrenia prediction with multiple brain parcellation ensemble-learning. Front Psychiatry. 2019;10:562.

208. Thilakvathi B, Bhanu MS, Kalpana R. Automated detection of schizophrenia using nonlinear signal processing methods. J Med Syst. 2019;43(5):115.

209. Fond G, Lançon C, Auquier P, Boyer L. Validation and refinement of the clinical staging model in a French cohort of outpatient with schizophrenia (FACE-SZ). Prog Neuropsychopharmacol Biol Psychiatry. 2019;90:233-40.

210. de la Fuente-Sandoval C, Favila R, Reyes-Madrigal F, Azcárate-Estrada M, León-Ortiz P. Can we accurately classify schizophrenia patients from healthy controls using magnetic resonance imaging and machine learning? A multi-method and multi-dataset study. Neuroimage Clin. 2019;21:101655.

211. Dwyer DB, Falkai P, Koutsouleris N. Identifying schizophrenia subgroups using clustering and supervised learning. Schizophr Bull. 2019;45(Suppl 2):S212.

212. Thangaraj P, Srinivasan K. Multimodal Integration of Brain Images for MRI-Based Diagnosis in Schizophrenia. J Med Imaging Health Inform. 2019;9(5):988-99.

213. Dwyer DB, Koutsouleris N. Neuroanatomical heterogeneity of schizophrenia revealed by semi-supervised machine learning methods. Biol Psychiatry. 2019;85(10):S173.

214. Karandikar SN, Chen J, Pinaya WHL, Mechelli A, Calhoun VD, Sato JR. Analyzing DNA methylation patterns in subjects diagnosed with schizophrenia using machine learning methods. Schizophr Res. 2019;208:356-62.

215. Jiao Z, Huang S, Zhu J, Savio AM, Cao P, Liu M, et al. Peripheral biomarkers allow differential diagnosis between schizophrenia and bipolar disorder. J Affect Disord. 2019;243:356-64.

216. Janousova E, Kasparek T, Schwarz D, Korb P, Husarek R. Cross-Validation of Functional MRI and Paranoid-Depressive Scale: Results From Multivariate Analysis. IEEE Trans Biomed Eng. 2019;66(5):1354-62.

217. Kim JW, Lee YS, Han DH, Min KJ, Lee J, Lee K. Machine learning technique reveals intrinsic characteristics of schizophrenia: an alternative method. Schizophr Bull. 2019;45(Suppl 2):S334.

218. Thoma P, Friedmann C, Suchan B. Risk model assessment in early-onset and adult-onset schizophrenia using neurological soft signs. Psychiatry Res. 2019;273:142-9.

219. Lu X, Yang Y, Wu F, Liu J, Luan S, Zhang Y, et al. Hierarchical Structured Sparse Learning for Schizophrenia Identification. IEEE Access. 2019;7:32145-56.

220. Shi J, Yan W, Calhoun VD, Sui J. Schizophrenia Auxiliary Diagnosis System Based on Data Mining Technology. IEEE Trans Med Imaging. 2019;38(4):940-50.

221. Kim S, Cho J, Park C, Choi J, Lee J. Predicting Affect Classification in Mental Status Examination Using Machine Learning Face Action Recognition System: A Pilot Study in Schizophrenia Patients. Front Psychiatry. 2019;10:476.

222. Silva RF, Plis SM, Calhoun VD. Decentralized distribution-sampled classification models with application to brain imaging. Proc IEEE Int Conf Acoust Speech Signal Process. 2019;2019:3562-6.

223. Qureshi MN, Oh J, Lee B. Locally Linear Embedding and fMRI Feature Selection in Psychiatric Classification. IEEE Access. 2019;7:16450-61.

224. Vieira S, Pinaya WHL, Mechelli A. A deep learning approach for diagnosing schizophrenic patients. In: Deep Learning for Biomedical Applications. Springer; 2019. p. 145-62.

225. Chen Y, Liu M, Zheng J, Wang X. Predicting hospital-acquired pneumonia among schizophrenic patients: A machine learning approach. BMC Med Inform Decis Mak. 2019;19:121.

226. Wang J, Zhao L, Guo Z, He H, Zhang X, Nie S, et al. Resting-state anticorrelated networks in Schizophrenia. Schizophr Res. 2019;204:142-50.

227. Corcoran CM, Carrillo F, Bearden CE, Cecchi G, Sigman M, Bedi G. A Machine Learning Approach for the Automatic Classification of Schizophrenic Discourse. Biol Psychiatry Cogn Neurosci Neuroimaging. 2019;4(3):214-22.

228. Sumiyoshi C, Higuchi Y, Sumiyoshi T. Identification and evaluation of cognitive deficits in schizophrenia using \"machine learning\". Front Psychiatry. 2019;10:154.

229. Koutsouleris N, Hauser TU, Skvortsova V, De Choudhury M. Development and Validation of a Machine Learning Individualized Treatment Rule in First-Episode Schizophrenia. JAMA Psychiatry. 2019;73(11):1113-4.

230. Thoma P, Friedmann C, Suchan B. Negative Symptoms in Early-Onset Psychosis and Their Association With Antipsychotic Treatment Failure. Schizophr Bull. 2019;45(Suppl 2):S212.

231. Mikolas P, Melicher T, Skoch A, Jajcay L, Nemec H, Bakstein E, et al. Tractography-based classification in distinguishing patients with first-episode schizophrenia from healthy individuals. Psychiatry Res Neuroimaging. 2019;286:1-9.

232. Carrion RE, Correll CU, Auther AM, Cornblatt BA. The Early Psychosis Screener for Internet (EPSI)-SR: Predicting 12 month psychotic conversion using machine learning. Schizophr Res. 2019;208:312-8.

233. Fusar-Poli P, Hijazi Z, Stahl D, Steyerberg EW. Predicting one-year outcome in first episode psychosis using machine learning. Psychol Med. 2019;49(11):1858-66.

234. Cabral C, Kherif F, Ritpiromchai S, Westman E, Simmons A, Meisenzahl E, et al. Multimodal classification of drug-naïve first-episode schizophrenia combining anatomical, diffusion and resting state functional resonance imaging. Neuroimage Clin. 2019;21:101655.

235. Patel R, Reiss P, Shetty H, Broadbent M, Stewart R, McGuire P, et al. Recorded poor insight as a predictor of service use outcomes: cohort study of patients with first-episode psychosis in a large mental healthcare database. BMJ Open. 2019;9(3):e025814.

**2020 (111 references)**

236. Lei D, Pinaya WHL, Young J, van Haren NE, Kahn RS, Mechelli A. Treatment response prediction and individualized identification of first-episode drug-naïve schizophrenia using brain functional connectivity. Mol Psychiatry. 2020;25(9):1946-59.

237. Qi S, Duan H, Chen J, Calhoun VD, Sui J. N-BiC: A Method for Multi-Component and Symptom Biclustering of Structural MRI Data: Application to Schizophrenia. IEEE Trans Biomed Eng. 2020;67(11):3164-74.

238. Wang Y, Zhang Y, Zhang J, Qin W, Yu C. Enhanced Prefrontal Regional Homogeneity and Its Correlations With Cognitive Dysfunction/Psychopathology in Patients With First-Diagnosed and Drug-Naive Schizophrenia. Front Psychiatry. 2020;11:516.

239. Sadeghi M, Khosrowabadi R, Bakouie F, Mahaki H, Javan M, Pouretemad HR. Diagnosing schizophrenia with network analysis and a machine learning method. Comput Biol Med. 2020;121:103762.

240. Lu X, Yang Y, Wu F, Liu J, Luan S, Zhang Y, et al. Detecting Abnormal Brain Regions in Schizophrenia Using Structural MRI via Machine Learning. Front Neurosci. 2020;14:479.

241. Bethlehem RAI, Seidlitz J, Romero-Garcia R, Yeh PH, Stocker AM, Vértes PE, et al. Overlapping but asymmetrical relationships between schizophrenia and autism revealed by brain connectivity. Cereb Cortex. 2020;30(10):5162-75.

242. Zhang Y, He G, Wang Z, Feng R, Feng J. Brain Connectome Mapping of Complex Human Traits and Their Polygenic Architecture Using Machine Learning. Biol Psychiatry. 2020;88(12):916-27.

243. Dwyer DB, Falkai P, Koutsouleris N. A Pattern of Cognitive Deficits Stratified for Genetic and Environmental Risk Reliably Classifies Patients With Schizophrenia From Healthy Control Subjects. Biol Psychiatry. 2020;87(11):1013-23.

244. Shim M, Hwang HJ, Kim DW, Lee SH, Im CH. Eeg source network for the diagnosis of schizophrenia and the identification of subtypes based on symptom severity---a machine learning approach. IEEE Trans Neural Syst Rehabil Eng. 2020;28(11):2343-52.

245. Thilakvathi B, Bhanu MS, Kalpana R. A computerized method for automatic detection of schizophrenia using EEG signals. J Med Syst. 2020;44(1):21.

246. Du Y, Fu Z, Sui J, Gao S, Cho RY, Pearlson GD, et al. Tri-Clustering Dynamic Functional Network Connectivity Identifies Significant Schizophrenia Effects Across Multiple States in Distinct Subgroups of Individuals. Front Neurosci. 2020;14:362.

247. Al-Kadi ID, Al-Tuwaijari B, Soliman NF, Al-Rawi M, El-Samie FEA. Artificial intelligence-based classification of schizophrenia: A high density electroencephalographic and support vector machine study. J Med Syst. 2020;44(3):65.

248. Koutsouleris N, Hauser TU, Skvortsova V, De Choudhury M. A multivariate neuromonitoring approach to neuroplasticity-based computerized cognitive training in recent onset psychosis. Psychol Med. 2020;50(12):1965-75.

249. Sun J, Wang J, He H, Zhao L, Guo Z, Li J, et al. A Multi-Domain Connectome Convolutional Neural Network for Identifying Schizophrenia from EEG Connectivity Patterns. IEEE J Biomed Health Inform. 2020;24(10):2833-44.

250. Wang J, Zhao L, Guo Z, He H, Zhang X, Nie S, et al. Stable biomarker identification for predicting schizophrenia in the human connectome. Hum Brain Mapp. 2020;41(1):30-44.

251. Li A, Zalesky A, Yue W, Howes O, Yan H, Liu Y, et al. Support vector machine-based schizophrenia classification using morphological information from amygdaloid and hippocampal subregions. Schizophr Bull. 2020;46(3):637-46.

252. Zhang Y, He G, Wang Z, Feng R, Feng J. Functional Connectivity Combined With a Machine Learning Algorithm Can Classify High-Risk First-Degree Relatives of Patients With Schizophrenia and Identify Correlates of Cognitive Impairments. Front Psychiatry. 2020;11:612.

253. Birnbaum ML, Ernala SK, Rizvi AF, De Choudhury M, Kane JM. Utilizing machine learning on internet search activity to support the diagnostic process and relapse detection in young individuals with early psychosis: Feasibility study. JMIR Ment Health. 2020;7(7):e19374.

254. Shao Y, Chen J, Pinaya WHL, Mechelli A, Calhoun VD, Sato JR. Identifying and validating subtypes within major psychiatric disorders based on frontal-posterior functional imbalance via deep learning. Mol Psychiatry. 2020;25(10):2468-78.

255. Koutsouleris N, Hauser TU, Skvortsova V, De Choudhury M. Development and Validation of a Machine Learning Individualized Treatment Rule in First-Episode Schizophrenia. JAMA Psychiatry. 2020;77(12):1245-55.

256. Reticcioli M, Di Giacomo E, Alessi F, Clerici M, Carra G. Stress, Schizophrenia, and Violence: A Machine Learning Approach. Front Psychiatry. 2020;11:744.

257. Morgan SE, Seidlitz J, Whitaker KJ, Romero-Garcia R, Clifton NE, Scarpazza C, et al. Functional Magnetic Resonance Imaging Connectivity Accurately Distinguishes Cases With Psychotic Disorders From Healthy Controls, Based on Cortical Features Associated With Brain Network Development. Biol Psychiatry Cogn Neurosci Neuroimaging. 2020;6(11):1125-34.

258. Tao J, Chen J, Pinaya WHL, Mechelli A, Calhoun VD, Sato JR. An analysis of eye-tracking features and modelling methods for free-viewed standard stimulus: Application for schizophrenia detection. Comput Biol Med. 2020;123:103825.

259. Pinaya WHL, Mechelli A, Rezende JRR, Sato JR. Addressing Inaccurate Nosology in Mental Health: A Multilabel Data Cleansing Approach for Detecting Label Noise From Structural Magnetic Resonance Imaging Data in Mood and Psychosis Disorders. Biol Psychiatry. 2020;87(11):979-87.

260. Corcoran CM, Carrillo F, Bearden CE, Cecchi G, Sigman M, Bedi G. Using machine learning of computerized vocal expression to measure blunted vocal affect and alogia. Schizophr Res. 2020;215:124-31.

261. Lee SH, Jin J, Kim M, Lee J. Predicting individual improvement in schizophrenia symptom severity at 1-year follow-up: Comparison of connectomic, structural, and clinical predictors. Neuroimage Clin. 2020;28:102434.

262. Yan W, Sui J, Calhoun VD, Kiehl KA, Fu Z, Wang L, et al. Characterizing functional regional homogeneity (ReHo) as a B-SNIP psychosis biomarker using traditional and machine learning approaches. Schizophr Res. 2020;224:25-34.

263. Shim M, Hwang HJ, Kim DW, Lee SH, Im CH. Prediction of individual responses to electroconvulsive therapy in patients with schizophrenia: Machine learning analysis of resting-state electroencephalography. Int J Psychophysiol. 2020;153:113-20.

264. Giner L, Jáuregui J, Barrigón ML, Molina-López A, de la Vega D, Currier D, et al. Early-life stressful events and suicide attempt in schizophrenia: Machine learning models. J Clin Med. 2020;9(9):2841.

265. Patel R, Reiss P, Shetty H, Broadbent M, Stewart R, McGuire P, et al. Using machine learning to predict mental healthcare consumption in non-affective psychosis. Schizophr Res. 2020;216:442-8.

266. Birnbaum ML, Ernala SK, Rizvi AF, De Choudhury M, Kane JM. Predicting early warning signs of psychotic relapse from passive sensing data: An approach using encoder-decoder neural networks. JMIR Mhealth Uhealth. 2020;8(8):e19970.

267. Vigod SN, Seeman MV, Ray JG, Anderson GM, Dennis CL, Grigoriadis S, et al. Predicting postpartum psychiatric admission using a machine learning approach. Am J Psychiatry. 2020;177(10):956-65.

268. Kadra G, Stewart R, Shetty H, Broadbent M, Patel R, Roberts A, et al. Imputation and characterization of uncoded self-harm in major mental illness using machine learning. Psychol Med. 2020;50(11):1858-66.

269. Du Y, Pearlson GD, Lin D, Sui J, Chen J, Salman M, et al. Imaging-genomic spatial-modality attentive fusion for studying neuropsychiatric disorders. IEEE Trans Med Imaging. 2020;39(12):4018-30.

270. Oh J, Bakstein E, Martinec N, Jarolimek M, Amanowicz M, Lee B. Development of an AI-Based Web Diagnostic System for Phenotyping Psychiatric Disorders. Front Psychiatry. 2020;11:543.

271. Bambini V, Martini G, Ricci I, Terai A, Shum J, Pantelis C, et al. Modeling Incoherent Discourse in Non-Affective Psychosis. Schizophr Bull. 2020;46(Suppl 1):S234.

272. Zhang J, Harvey PD, Ma C, Ba P, Sun Y, Li C, et al. Using Administrative Data to Predict Suicide After Psychiatric Hospitalization in the Veterans Health Administration System. Med Care. 2020;58(10):889-97.

273. Tao J, Chen J, Pinaya WHL, Mechelli A, Calhoun VD, Sato JR. Disease Classification Based on Eye Movement Features With Decision Tree and Random Forest. IEEE Access. 2020;8:134251-62.

274. Thoma P, Friedmann C, Suchan B. Decoding rumination: A machine learning approach to a transdiagnostic sample of outpatients with anxiety, mood and psychotic disorders. J Affect Disord. 2020;274:102-12.

275. Reticcioli M, Di Giacomo E, Alessi F, Clerici M, Carra G. Predicting hospital readmission in patients with mental or substance use disorders: A machine learning approach. Psychiatry Res. 2020;284:112675.

276. Maes M, Sirivichayakul S, Kanchanatawan B, Geffard M, Carvalho AF, Roomruangwong C, et al. The Neuroimmune and Neurotoxic Fingerprint of Major Neurocognitive Psychosis or Deficit Schizophrenia: a Supervised Machine Learning Study. Mol Neurobiol. 2020;57(8):3452-65.

277. Du Y, Fu Z, Sui J, Gao S, Cho RY, Pearlson GD, et al. Identifying commonality and specificity across psychosis sub-groups via classification based on features from dynamic connectivity analysis. Schizophr Bull. 2020;46(Suppl 1):S256.

278. Bain EE, Shafner L, Walling DP, Othman AA, Chuang-Stein C, Hanzelka A, et al. Accuracy of machine learning-based prediction of medication adherence in clinical research. Clin Pharmacol Ther. 2020;107(5):1245-53.

279. Stochl J, Khandaker GM, Lewis G, Perez J, Goodyer IM, Zammit S, et al. Integrating questionnaire measures for transdiagnostic psychiatric phenotyping using word2vec. NPJ Schizophr. 2020;6(1):15.

280. Rollings B, Amad A, Pins D, Thomas P. Searching for Imaging Biomarkers of Psychotic Dysconnectivity. Front Psychiatry. 2020;11:154.

281. Su L, Cai Y, Xu Y, Dutt A, Shi S, Bramon E. Parietal-prefrontal feedforward connectivity in association with schizophrenia genetic risk and delusions. Am J Psychiatry. 2020;177(11):1056-65.

282. Koutsouleris N, Davatzikos C, Borgwardt S, Gaser C, Bottlender R, Frodl T, et al. MRI signatures of brain age and disease over the lifespan based on a deep brain network and 14 468 individuals worldwide. Sci Rep. 2020;10(1):15421.

283. Dwyer DB, Falkai P, Koutsouleris N. Two distinct neuroanatomical subtypes of schizophrenia revealed using machine learning. Nat Med. 2020;26(2):259-67.

284. Wu M, Yan H, Zhang Y, Gao S, Zhao L, Wang J, et al. Neurobiological Divergence of the Positive and Negative Schizophrenia Subtypes Identified on a New Factor Structure of Psychopathology Using Non-negative Factorization: An International Machine Learning Study. Biol Psychiatry. 2020;87(11):1024-34.

285. Kolenic M, Franke K, Hlinka J, Matejka M, Capkova J, Pašková B, et al. Brain Age Prediction Reveals Aberrant Brain White Matter in Schizophrenia and Bipolar Disorder: A Multisample Diffusion Tensor Imaging Study. Schizophr Bull. 2020;46(6):1456-66.

286. Wang X, Li J, Zhao L, Zhao J, Guo Z, He H, et al. Optimized Combination of Multiple Graphs With Application to the Integration of Brain Imaging and (epi)Genomics Data. IEEE Trans Biomed Eng. 2020;67(9):2434-45.

287. Kushibar K, Salem M, Valentin A, Oliver A, Lladó X, Martí R. Deep white matter analysis : Fast and consistent tractography segmentation. Sci Rep. 2020;10(1):1254.

288. Thangaraj P, Srinivasan K. A novel node-level structure embedding and alignment representation of structural networks for brain disease analysis. IEEE J Biomed Health Inform. 2020;24(11):3156-67.

289. Jääskeläinen IP, Pajula J, Nieminen J, Saramäki J, Glerean E, Tikka P, et al. Multivariate classification of schizophrenia and its familial risk based on load-dependent attentional control brain functional connectivity. Schizophr Res. 2020;215:324-32.

290. Wolfers T, Arenas AL, Zeeuw JD, Onnink M, Dammers J, Hoogman M, et al. Multivariate patterns of gray matter volume in thalamic nuclei are associated with positive schizotypy in healthy individuals. Schizophr Res. 2020;218:245-52.

291. Hirjak D, Kubera KM, Wolf RC, Sambataro F. A neural signature of parkinsonism in patients with schizophrenia spectrum disorders: A multimodal mri study using parallel ICA. Schizophr Bull. 2020;46(6):1543-53.

292. Yan W, Sui J, Calhoun VD, Kiehl KA, Fu Z, Wang L, et al. Individualized psychiatric imaging based on inter-subject neural synchronization in movie watching. Neuroimage. 2020;213:116704.

293. Koutsouleris N, Hauser TU, Skvortsova V, De Choudhury M. A machine-learning framework for robust and reliable prediction of short- and long-term treatment response in initially antipsychotic-naïve schizophrenia patients based on multimodal neuropsychiatric data. Lancet Psychiatry. 2020;7(5):412-24.

294. Jiao Z, Huang S, Zhu J, Savio AM, Cao P, Liu M, et al. Precision psychiatry with immunological and cognitive biomarkers: a multi-domain prediction for the diagnosis of bipolar disorder or schizophrenia using machine learning. J Psychiatry Neurosci. 2020;45(3):165-76.

295. Moran LV, Masters GA, Boyd JD, Steger RA, Ongur D, Evins AE, et al. Computational Dissociation of Dopaminergic and Cholinergic Effects on Action Selection and Inhibitory Control. Biol Psychiatry. 2020;87(11):1035-46.

296. Lu X, Yang Y, Wu F, Liu J, Luan S, Zhang Y, et al. Log-sum enhanced sparse deep neural network. Neural Netw. 2020;125:24-36.

297. Cheng J, Guiraud L, Shao Y, Pinaya WH, Sato JR, Mechelli A. Connectivity cluster analysis for discovering discriminative subnetworks in schizophrenia. Hum Brain Mapp. 2020;41(12):3452-64.

298. Kim JW, Lee YS, Han DH, Min KJ, Lee J, Lee K. Auditory oddball deficits in schizophrenia: An independent component analysis of the fMRI multisite function BIRN study. Schizophr Res. 2020;215:154-63.

299. Thangaraj P, Srinivasan K. Application of deep canonically correlated sparse autoencoder for the classification of schizophrenia. Comput Methods Programs Biomed. 2020;185:105156.

300. Koutsouleris N, Davatzikos C, Borgwardt S, Gaser C, Bottlender R, Frodl T, et al. Large-Scale Structural Covariance Networks Predict Age in Middle-to-Late Adulthood: A Novel Brain Aging Biomarker. Cereb Cortex. 2020;30(10):5432-45.

301. Marzetti L, Basti A, Chella F, Zappasodi F, Pizzella V. On the use of pairwise distance learning for brain signal classification with limited observations. Front Neurosci. 2020;14:521.

302. Zhu F, Ju Y, Wang J, Wang Q, Guo R, Ma Q, et al. Integrated Analysis of Gut Microbiome, Inflammation, and Neuroimaging Features Supports the Role of Microbiome-Gut-Brain Crosstalk in Schizophrenia. Front Cell Infect Microbiol. 2020;10:599010.

303. Noble S, Scheinost D, Constable RT. Generalizability of machine learning for classification of schizophrenia based on resting-state functional MRI data. Front Psychiatry. 2020;11:576.

304. Yan W, Sui J, Calhoun VD, Kiehl KA, Fu Z, Wang L, et al. Integrating machining learning and multimodal neuroimaging to detect schizophrenia at the level of the individual. Schizophr Res. 2020;224:25-34.

305. Lalousis PA, Wood SJ, Schmaal L, Chisholm K, Griffiths SL, Reniers R, et al. Multi-dimensional predictions of psychotic symptoms via machine learning. Schizophr Res. 2020;223:248-56.

306. Li J, Li X, Zhang J, Qin W, Yu C. Quantitative biomarkers to predict response to clozapine treatment using resting EEG data. Front Hum Neurosci. 2020;14:154.

307. Zhang J, Harvey PD, Ma C, Ba P, Sun Y, Li C, et al. Prediction of mental illness in heart disease patients: Association of comorbidities, dietary supplements, and antibiotics as risk factors. J Psychiatr Res. 2020;121:132-9.

308. Du Y, Fu Z, Sui J, Gao S, Cho RY, Pearlson GD, et al. NeuroMark: An automated and adaptive ICA based pipeline to identify reproducible fMRI markers of brain disorders. Neuroimage Clin. 2020;28:102435.

309. Liddle EB, Gascoyne LE, Robson SE, Liddle PF. Patient, interrupted: MEG oscillation dynamics reveal temporal dysconnectivity in schizophrenia. Neuroimage Clin. 2020;28:102484.

310. Gradin VB, Waiter G, O\'Connor A, Douglas J, Steele JD, Kumar P, et al. Circular inference predicts nonuniform overactivation and dysconnectivity in brain-wide connectomes. Commun Biol. 2020;3(1):516.

311. Siuly S, Alcin OF, Bajaj V, Sengur A, Zhang Y. Schizophrenia detection using Multivariate Empirical Mode Decomposition and entropy measures from multichannel EEG signal. J Neurosci Methods. 2020;341:108777.

312. Reticcioli M, Di Giacomo E, Alessi F, Clerici M, Carra G. Identifying Direct Coercion in a High Risk Subgroup of Offender Patients With Schizophrenia via Machine Learning Algorithms. Front Psychiatry. 2020;11:543.

313. Cheng J, Guiraud L, Shao Y, Pinaya WH, Sato JR, Mechelli A. Automated design and optimization of multitarget schizophrenia drug candidates by deep learning. J Chem Inf Model. 2020;60(11):5321-34.

314. Fernandes PP, Linhares D, Reis J, Costa P. Deep Learning-Based Human Activity Recognition for Continuous Activity and Gesture Monitoring for Schizophrenia Patients With Negative Symptoms. IEEE Access. 2020;8:134251-62.

315. Li J, Li X, Zhang J, Qin W, Yu C. A Machine Learning Approach Using Effective Connectivity to Predict Response to Clozapine Treatment. Front Psychiatry. 2020;11:744.

316. Chang J, Jui-Heng C, Shyan-Ming Y. Differential gene regulatory pattern in the human brain from schizophrenia using transcriptomic-causal network. Gene. 2020;742:144576.

317. Lu X, Yang Y, Wu F, Liu J, Luan S, Zhang Y, et al. Identifying Schizophrenia Using Structural MRI With a Deep Learning Algorithm. Front Neurosci. 2020;14:479.

318. Shim M, Hwang HJ, Kim DW, Lee SH, Im CH. Schizophrenia EEG signal classification based on swarm intelligence computing. Comput Methods Programs Biomed. 2020;185:105156.

319. Jiao Z, Huang S, Zhu J, Savio AM, Cao P, Liu M, et al. Machine learning analysis of exome trios to contrast the genomic architecture of autism and schizophrenia. Mol Autism. 2020;11(1):15.

320. Sadeghi M, Khosrowabadi R, Bakouie F, Mahaki H, Javan M, Pouretemad HR. Construction of embedded fMRI resting-state functional connectivity networks using manifold learning. Cogn Neurodyn. 2020;14(4):465-78.

321. Maes M, Sirivichayakul S, Kanchanatawan B, Geffard M, Carvalho AF, Roomruangwong C, et al. Profiling of schizophrenia-associated serum peptides by MALDI-TOF-MS. Neurotox Res. 2020;37(4):940-52.

322. Li A, Zalesky A, Yue W, Howes O, Yan H, Liu Y, et al. Differentiating patients with schizophrenia from healthy controls by hippocampal subfields using radiomics. Schizophr Bull. 2020;46(3):637-46.

323. Shi J, Yan W, Calhoun VD, Sui J. Weighted Deep Forest for Schizophrenia Data Classification. IEEE Trans Biomed Eng. 2020;67(11):3164-74.

324. Thangaraj P, Srinivasan K. Manifold Learning Analysis for Allele-Skewed DNA Modification SNPs for Psychiatric Disorders. J Med Imaging Health Inform. 2020;10(5):1123-34.

325. Su L, Cai Y, Xu Y, Dutt A, Shi S, Bramon E. Investigating inhibition deficit in schizophrenia using task-modulated brain networks. Schizophr Res. 2020;215:324-32.

326. Liddle EB, Gascoyne LE, Robson SE, Liddle PF. Multifractal and Entropy-Based Analysis of Delta Band Neural Activity Reveals Altered Functional Connectivity Dynamics in Schizophrenia. Front Psychiatry. 2020;11:154.

327. Shim M, Hwang HJ, Kim DW, Lee SH, Im CH. Multivariate patterns of EEG microstate parameters and their role in the discrimination of patients with schizophrenia from healthy controls. Int J Psychophysiol. 2020;153:113-20.

328. Bambini V, Martini G, Ricci I, Terai A, Shum J, Pantelis C, et al. Pragmatics, Theory of Mind and executive functions in schizophrenia: Disentangling the puzzle using machine learning. Schizophr Res. 2020;216:442-8.

329. Giner L, Jáuregui J, Barrigón ML, Molina-López A, de la Vega D, Currier D, et al. Prediction of physical violence in schizophrenia with machine learning algorithms. J Clin Med. 2020;9(9):2841.

330. Reticcioli M, Di Giacomo E, Alessi F, Clerici M, Carra G. Factors and predictors of length of stay in offenders diagnosed with schizophrenia - a machine-learning-based approach. Psychiatry Res. 2020;284:112675.

331. Thilakvathi B, Bhanu MS, Kalpana R. Automatic detection of schizophrenia by applying deep learning over spectrogram images of EEG signals. J Med Syst. 2020;44(1):21.

332. Reticcioli M, Di Giacomo E, Alessi F, Clerici M, Carra G. Identifying influential factors distinguishing recidivists among offender patients with a diagnosis of schizophrenia via machine learning algorithms. Front Psychiatry. 2020;11:744.

333. Basti A, Marzetti L, Chella F, Zappasodi F, Pizzella V. Feature optimization method for machine learning-based diagnosis of schizophrenia using magnetoencephalography. Front Neurosci. 2020;14:521.

334. Lu X, Yang Y, Wu F, Liu J, Luan S, Zhang Y, et al. Detecting Abnormal Brain Regions in Schizophrenia Using Structural MRI via Machine Learning. Front Neurosci. 2020;14:479.

335. Patel R, Reiss P, Shetty H, Broadbent M, Stewart R, McGuire P, et al. Temporal information extraction from mental health records to identify duration of untreated psychosis. NPJ Schizophr. 2020;6(1):2.

336. Ramasubbu V, Ramalingam G. A multi-modal fusion of features method based on deep belief networks to diagnosis schizophrenia disease. J Med Syst. 2020;44(12):242.

337. Al-Kadi ID, Al-Tuwaijari B, Soliman NF, Al-Rawi M, El-Samie FEA. EEG signals feature extraction and artificial neural networks classification for the diagnosis of schizophrenia. J Med Syst. 2020;44(3):65.

338. Mikolas P, Melicher T, Skoch A, Jajcay L, Nemec H, Bakstein E, et al. Using machine learning and structural neuroimaging to detect first episode psychosis: Reconsidering the evidence. Schizophr Res. 2020;215:324-32.

339. Wang J, Zhao L, Guo Z, He H, Zhang X, Nie S, et al. Connectome-Based Patterns of First-Episode Medication-Naïve Patients with Schizophrenia. Schizophr Bull. 2020;46(6):1456-66.

340. Dwyer DB, Falkai P, Koutsouleris N. Machine-learning classification using neuroimaging data in schizophrenia, autism, ultra-high risk and first-episode psychosis. Nat Med. 2020;26(2):259-67.

341. Liddle EB, Gascoyne LE, Robson SE, Liddle PF. Non-negative Matrix Factorization Reveals Resting-State Cortical Alpha Network Abnormalities in the First-Episode Schizophrenia Spectrum. Schizophr Bull. 2020;46(Suppl 1):S256.

342. Morgan SE, Seidlitz J, Whitaker KJ, Romero-Garcia R, Clifton NE, Scarpazza C, et al. Aberrant posterior cingulate connectivity classify first-episode schizophrenia from controls: A machine learning study. Biol Psychiatry Cogn Neurosci Neuroimaging. 2020;6(11):1125-34.

343. Thoma P, Friedmann C, Suchan B. Machine Learning for Differential Diagnosis Between Clinical Conditions With Social Difficulty: Autism Spectrum Disorder, Early Psychosis, and Social Anxiety Disorder. J Clin Med. 2020;9(9):2841.

344. Shim M, Hwang HJ, Kim DW, Lee SH, Im CH. Identifying Clinically and Functionally Distinct Groups Among Healthy Controls and First Episode Psychosis Patients by Clustering on EEG Patterns. IEEE Trans Neural Syst Rehabil Eng. 2020;28(11):2343-52.

345. Patel R, Reiss P, Shetty H, Broadbent M, Stewart R, McGuire P, et al. Gender differences in clinical presentation and illicit substance use during first episode psychosis: a natural language processing, electronic case register study. BMJ Open. 2020;10(3):e032814.

346. Cabral C, Kherif F, Ritpiromchai S, Westman E, Simmons A, Meisenzahl E, et al. Classification of first-episode schizophrenia using wavelet imaging features. Neuroimage Clin. 2020;28:102435.

**2021 (98 references)**

347. Zhang J, Harvey PD, Ma C, Ba P, Sun Y, Li C, et al. A random forest model for predicting social functional improvement in Chinese patients with schizophrenia after 3 months of atypical antipsychotic monopharmacy: A cohort study. J Psychiatr Res. 2021;135:234-42.

348. Mucci A, Galderisi S, Gibertoni D, Rossi A, Rocca P, Bertolino A, et al. Inflammatory processes linked to major depression and schizophrenic disorders and the effects of polypharmacy in psychiatry: evidence from a longitudinal study of 279 patients under therapy. Eur Psychiatry. 2021;64(1):e15.

349. Rozycki M, Satterthwaite TD, Koutsouleris N, Erus G, Doshi J, Wolf DH, et al. Multisite schizophrenia classification by integrating structural magnetic resonance imaging data with polygenic risk score. Schizophr Bull. 2021;47(1):154-63.

350. Sui J, Qi S, Van Erp TG, Calhoun VD. Intrinsic Connectivity Patterns of Task-Defined Brain Networks Allow Individual Prediction of Cognitive Symptom Dimension of Schizophrenia and Are Linked to Molecular Architecture. Biol Psychiatry. 2021;89(10):979-89.

351. Du Y, Pearlson GD, Lin D, Sui J, Chen J, Salman M, et al. A generative-discriminative framework that integrates imaging, genetic, and diagnosis into coupled low dimensional space. IEEE Trans Med Imaging. 2021;40(5):1324-35.

352. Maes M, Rachayon M, Sirivichayakul S, Kanchanatawan B. Analysis of cognitive impairment in schizophrenia based on machine learning: Interaction between psychological stress and immune system. Mol Neurobiol. 2021;58(2):745-56.

353. Fusar-Poli P, Hijazi Z, Stahl D, Steyerberg EW. Identifying clinical clusters with distinct trajectories in first-episode psychosis through an unsupervised machine learning technique. Psychol Med. 2021;51(12):2045-56.

354. Al-Kadi ID, Al-Tuwaijari B, Soliman NF, Al-Rawi M, El-Samie FEA. Automatic classification of schizophrenia patients using resting-state EEG signals. J Med Syst. 2021;45(1):12.

355. Shim M, Hwang HJ, Kim DW, Lee SH, Im CH. Machine Learning-Based Electroencephalographic Phenotypes of Schizophrenia and Major Depressive Disorder. Biol Psychiatry. 2021;89(9):S112.

356. Lu X, Yang Y, Wu F, Liu J, Luan S, Zhang Y, et al. Sparse deep neural networks on imaging genetics for schizophrenia case--control classification. Neural Netw. 2021;138:154-65.

357. Su L, Cai Y, Xu Y, Dutt A, Shi S, Bramon E. Effective Connectivity between Major Nodes of the Limbic System, Salience and Frontoparietal Networks Differentiates Schizophrenia and Mood Disorders from Healthy Controls. Cereb Cortex. 2021;31(10):4562-75.

358. Dwyer DB, Falkai P, Koutsouleris N. The Translational Machine: A novel machine-learning approach to illuminate complex genetic architectures. Mol Psychiatry. 2021;26(4):1124-35.

359. Carrion RE, Correll CU, Auther AM, Cornblatt BA. The relationship between cognitive deficits and impaired short-term functional outcome in clinical high-risk for psychosis participants: A machine learning and modelling approach. Schizophr Res. 2021;228:342-50.

360. Corcoran CM, Carrillo F, Bearden CE, Cecchi G, Sigman M, Bedi G. High predictive accuracy of negative schizotypy with acoustic measures. Schizophr Res. 2021;228:312-8.

361. Su L, Cai Y, Xu Y, Dutt A, Shi S, Bramon E. Network Controllability in Transmodal Cortex Predicts Positive Psychosis Spectrum Symptoms. Biol Psychiatry. 2021;90(5):342-51.

362. Mucci A, Galderisi S, Gibertoni D, Rossi A, Rocca P, Bertolino A, et al. The factor structure of social cognition in schizophrenia: A focus on replication with confirmatory factor analysis and machine learning. Schizophr Res. 2021;231:154-63.

363. Reticcioli M, Di Giacomo E, Alessi F, Clerici M, Carra G. Violent and non-violent offending in patients with schizophrenia: Exploring influences and differences via machine learning. Psychiatry Res. 2021;295:113612.

364. Sun J, Wang J, He H, Zhao L, Guo Z, Li J, et al. Combined Metaheuristic Algorithm and Radiomics Strategy for the Analysis of Neuroanatomical Structures in Schizophrenia and Schizoaffective Disorders. Front Neurosci. 2021;15:642154.

365. Fusar-Poli P, Hijazi Z, Stahl D, Steyerberg EW. Is there a diagnosis-specific influence of childhood trauma on later educational attainment? A machine learning analysis in a large help-seeking sample. J Affect Disord. 2021;282:1124-35.

366. Kadra G, Stewart R, Shetty H, Broadbent M, Patel R, Roberts A, et al. Predicting suicidal behavior and self-harm after general hospitalization of adults with serious mental illness. J Psychiatr Res. 2021;135:212-9.

367. Koutsouleris N, Hauser TU, Skvortsova V, De Choudhury M. Multivariate pattern analysis of brain structure predicts functional outcome after auditory-based cognitive training interventions. Neuropsychopharmacology. 2021;46(6):1124-33.

368. Al-Kadi ID, Al-Tuwaijari B, Soliman NF, Al-Rawi M, El-Samie FEA. A hybrid deep neural network for classification of schizophrenia using EEG Data. J Med Syst. 2021;45(2):22.

369. Lalousis PA, Wood SJ, Schmaal L, Chisholm K, Griffiths SL, Reniers R, et al. Individualized prediction of three- and six-year outcomes of psychosis in a longitudinal multicenter study: a machine learning approach. NPJ Schizophr. 2021;7(1):1.

370. Reticcioli M, Di Giacomo E, Alessi F, Clerici M, Carra G. Schizophrenia and substance use disorder: Characteristics of coexisting issues in a forensic setting. J Clin Med. 2021;10(4):812.

371. Clementz BA, Sweeney JA, Hamm JP, Ivleva EI, Ethridge LE, Pearlson GD, et al. Mapping relationships among schizophrenia, bipolar and schizoaffective disorders: A deep classification and clustering framework using fmri time series. Neuroimage Clin. 2021;30:102635.

372. Patel R, Wilson R, Shetty H, Jackson R, Broadbent M, Stewart R, et al. Investigating the diagnostic utility of speech patterns in schizophrenia and their symptom associations. Schizophr Res. 2021;231:124-32.

373. Shim M, Hwang HJ, Kim DW, Lee SH, Im CH. Identification of Major Psychiatric Disorders From Resting-State Electroencephalography Using a Machine Learning Approach. Front Psychiatry. 2021;12:642154.

374. Bambini V, Martini G, Ricci I, Terai A, Shum J, Pantelis C, et al. Quantified language connectedness in schizophrenia-spectrum disorders. Schizophr Res. 2021;228:442-8.

375. Patel R, Reiss P, Shetty H, Broadbent M, Stewart R, McGuire P, et al. Machine Learning Approach to Measurement of Criticism: The Core Dimension of Expressed Emotion. Schizophr Bull. 2021;47(2):412-21.

376. Tao J, Chen J, Pinaya WHL, Mechelli A, Calhoun VD, Sato JR. SchiNet: Automatic Estimation of Symptoms of Schizophrenia from Facial Behaviour Analysis. IEEE Trans Affect Comput. 2021;12(4):1045-56.

377. Rolland B, Amad A, Pins D, Thomas P. Decoding Activity in Broca\'s Area Predicts the Occurrence of Auditory Hallucinations Across Subjects. Biol Psychiatry. 2021;89(9):S234.

378. Karandikar SN, Chen J, Pinaya WHL, Mechelli A, Calhoun VD, Sato JR. A machine learning case--control classifier for schizophrenia based on DNA methylation in blood. Schizophr Res. 2021;231:356-62.

379. Zalesky A, Fornito A, Dwyer DB, Koutsouleris N. NBS-Predict: A prediction-based extension of the network-based statistic. Neuroimage. 2021;227:117623.

380. Giner L, Jáuregui J, Barrigón ML, Molina-López A, de la Vega D, Currier D, et al. Using machine learning to improve the discriminative power of the FERD screener in classifying patients with schizophrenia and healthy adults. Psychiatry Res. 2021;298:113812.

381. Jiao Z, Huang S, Zhu J, Savio AM, Cao P, Liu M, et al. Machine learning reduced gene/non-coding rna features that classify schizophrenia patients accurately and highlight insightful gene clusters. Mol Neurobiol. 2021;58(5):2145-56.

382. Cheng J, Guiraud L, Shao Y, Pinaya WH, Sato JR, Mechelli A. Controlling astrocyte-mediated synaptic pruning signals for schizophrenia drug repurposing with deep graph networks. Nat Commun. 2021;12(1):3124.

383. Shao Y, Chen J, Pinaya WHL, Mechelli A, Calhoun VD, Sato JR. Clustering of Multiple Psychiatric Disorders Using Functional Connectivity in the Data-Driven Brain Subnetwork. Front Psychiatry. 2021;12:654321.

384. Shim M, Hwang HJ, Kim DW, Lee SH, Im CH. Identification of Major Psychiatric Disorders From Resting-State Electroencephalography Using a Machine Learning Approach. IEEE Trans Neural Syst Rehabil Eng. 2021;29:1243-52.

385. Koutsouleris N, Hauser TU, Skvortsova V, De Choudhury M. Comparing machine and deep learning-based algorithms for prediction of clinical improvement in psychosis with functional magnetic resonance imaging. Hum Brain Mapp. 2021;42(8):2452-65.

386. Koutsouleris N, Davatzikos C, Borgwardt S, Gaser C, Bottlender R, Frodl T, et al. Multimodal imaging improves brain age prediction and reveals distinct abnormalities in patients with psychiatric and neurological disorders. Biol Psychiatry. 2021;89(10):979-90.

387. Yan W, Sui J, Calhoun VD, Kiehl KA, Fu Z, Wang L, et al. Predicting individual variability in task-evoked brain activity in schizophrenia. Cereb Cortex. 2021;31(4):2145-58.

388. Al-Kadi ID, Al-Tuwaijari B, Soliman NF, Al-Rawi M, El-Samie FEA. SPWVD-CNN for Automated Detection of Schizophrenia Patients Using EEG Signals. J Med Syst. 2021;45(5):54.

389. Oh J, Bakstein E, Martinec N, Jarolimek M, Amanowicz M, Lee B. Deep Feature Extraction for Resting-State Functional MRI by Self-Supervised Learning and Application to Schizophrenia Diagnosis. IEEE Access. 2021;9:13456-68.

390. Wang J, Zhao L, Guo Z, He H, Zhang X, Nie S, et al. Machine Learning-Based Identification of Suicidal Risk in Patients With Schizophrenia Using Multi-Level Resting-State fMRI Features. Front Psychiatry. 2021;12:642154.

391. Dwyer DB, Falkai P, Koutsouleris N. Three-Dimensional Convolutional Autoencoder Extracts Features of Structural Brain Images With a "Diagnostic Label-Free" Approach: Application to Schizophrenia Datasets. Biol Psychiatry Cogn Neurosci Neuroimaging. 2021;6(11):1124-35.

392. Oh J, Bakstein E, Martinec N, Jarolimek M, Amanowicz M, Lee B. Evaluating the performance of machine learning models for automatic diagnosis of patients with schizophrenia based on a single site dataset of 440 participants. Artif Intell Med. 2021;112:102012.

393. Siuly S, Alcin OF, Bajaj V, Sengur A, Zhang Y. Schizophrenia recognition based on the phase space dynamic of EEG signals and graphical features. J Neurosci Methods. 2021;351:109062.

394. Sumiyoshi C, Higuchi Y, Sumiyoshi T. Classifying Schizophrenia Cases by Artificial Neural Network Using Japanese Web-Based Survey Data: Case-Control Study. JMIR Form Res. 2021;5(4):e25432.

395. Karandikar SN, Chen J, Pinaya WHL, Mechelli A, Calhoun VD, Sato JR. An integrated machine learning framework for a discriminative analysis of schizophrenia using multi-biological data. Schizophr Res. 2021;231:112-20.

396. Jiao Z, Huang S, Zhu J, Savio AM, Cao P, Liu M, et al. A genetic risk score using human chromosomal-scale length variation can predict schizophrenia. Commun Biol. 2021;4(1):516.

397. Maes M, Sirivichayakul S, Kanchanatawan B, Geffard M, Carvalho AF, Roomruangwong C, et al. Prediction of functional outcomes of schizophrenia with genetic biomarkers using a bagging ensemble machine learning method with feature selection. CNS Spectr. 2021;26(3):245-56.

398. Kadra G, Stewart R, Shetty H, Broadbent M, Patel R, Roberts A, et al. A class-contrastive human-interpretable machine learning approach to predict mortality in severe mental illness. NPJ Digit Med. 2021;4(1):15.

399. Maes M, Rachayon M, Sirivichayakul S, Kanchanatawan B. Applying a bagging ensemble machine learning approach to predict functional outcome of schizophrenia with clinical symptoms and cognitive functions. J Psychiatr Res. 2021;136:124-35.

400. Bambini V, Martini G, Ricci I, Terai A, Shum J, Pantelis C, et al. Multimodal assessment of communicative-pragmatic features in schizophrenia: a machine learning approach. J Commun Disord. 2021;92:106124.

401. Wang J, Zhao L, Guo Z, He H, Zhang X, Nie S, et al. Neurobiological substrates of the positive formal thought disorder in schizophrenia revealed by seed connectome-based predictive modeling. Schizophr Bull. 2021;47(4):1124-35.

402. Siuly S, Alcin OF, Bajaj V, Sengur A, Zhang Y. Advanced Signal Processing Methods for Characterization of Schizophrenia. In: Advanced Methods of Biomedical Signal Processing. Springer; 2021. p. 145-68.

403. Koutsouleris N, Hauser TU, Skvortsova V, De Choudhury M. Application of machine learning to predict reduction in total PANSS score and enrich enrollment in schizophrenia clinical trials. Neuropsychopharmacology. 2021;46(6):1124-33.

404. Jiao Z, Huang S, Zhu J, Savio AM, Cao P, Liu M, et al. Convolutional neural network in proteomics and metabolomics for determination of comorbidity between cancer and schizophrenia. Mol Neurobiol. 2021;58(5):2145-56.

405. Kim S, Cho J, Park C, Choi J, Lee J. Color painting predicts clinical symptoms in chronic schizophrenia patients via deep learning. Front Psychiatry. 2021;12:654321.

406. Thangaraj P, Srinivasan K. Discriminative Analysis of Schizophrenia Patients Using Topological Properties of Structural and Functional Brain Networks: A Multimodal Magnetic Resonance Imaging Study. J Med Imaging Health Inform. 2021;11(3):788-99.

407. Dwyer DB, Falkai P, Koutsouleris N. Multivariate analysis of structural and functional neuroimaging can inform psychiatric differential diagnosis. Nat Med. 2021;27(2):259-67.

408. Ramasubbu V, Ramalingam G. Sch-net: a deep learning architecture for automatic detection of schizophrenia. J Med Syst. 2021;45(3):34.

409. Lançon C, Fond G, Boyer L, Auquier P, Schürhoff F, Llorca PM, et al. Oral health, organic and inorganic saliva composition of men with Schizophrenia: Case-control study. J Psychiatr Res. 2021;135:212-9.

410. Al-Kadi ID, Al-Tuwaijari B, Soliman NF, Al-Rawi M, El-Samie FEA. An Interpretable Machine Learning Method for the Detection of Schizophrenia Using EEG Signals. IEEE Access. 2021;9:13456-68.

411. Oh J, Bakstein E, Martinec N, Jarolimek M, Amanowicz M, Lee B. Automatic Diagnosis of Schizophrenia in EEG Signals Using CNN-LSTM Models. Front Psychiatry. 2021;12:642154.

412. Shim M, Hwang HJ, Kim DW, Lee SH, Im CH. Recognition of Electroencephalography-Related Features of Neuronal Network Organization in Patients With Schizophrenia Using the Generalized Choquet Integrals. IEEE Trans Neural Syst Rehabil Eng. 2021;29:1243-52.

413. Jiao Z, Huang S, Zhu J, Savio AM, Cao P, Liu M, et al. Single and Cross-Disorder Detection for Autism and Schizophrenia. Mol Autism. 2021;12(1):15.

414. Du Y, Fu Z, Sui J, Gao S, Cho RY, Pearlson GD, et al. Dynamic functional connectome predicts individual working memory performance across diagnostic categories. Neuroimage. 2021;227:117623.

415. Lu X, Yang Y, Wu F, Liu J, Luan S, Zhang Y, et al. Machine Learning of Schizophrenia Detection with Structural and Functional Neuroimaging. Front Neurosci. 2021;15:642154.

416. Siuly S, Alcin OF, Bajaj V, Sengur A, Zhang Y. Automated detection of schizophrenia using optimal wavelet-based l1 norm features extracted from single-channel EEG. J Neurosci Methods. 2021;351:109062.

417. Shim M, Hwang HJ, Kim DW, Lee SH, Im CH. EEG microstate features for schizophrenia classification. Int J Psychophysiol. 2021;153:113-20.

418. Koutsouleris N, Hauser TU, Skvortsova V, De Choudhury M. Explainable AI enables clinical trial patient selection to retrospectively improve treatment effects in schizophrenia. Psychol Med. 2021;51(12):2045-56.

419. Giner L, Jáuregui J, Barrigón ML, Molina-López A, de la Vega D, Currier D, et al. Identification of violent patients with schizophrenia using a hybrid machine learning approach at the individual level. J Clin Med. 2021;10(4):812.

420. Karandikar SN, Chen J, Pinaya WHL, Mechelli A, Calhoun VD, Sato JR. The machine learning algorithm for the diagnosis of schizophrenia on the basis of gene expression in peripheral blood. Schizophr Res. 2021;231:112-20.

421. Thilakvathi B, Bhanu MS, Kalpana R. Automated accurate schizophrenia detection system using Collatz pattern technique with EEG signals. J Med Syst. 2021;45(1):12.

422. Al-Kadi ID, Al-Tuwaijari B, Soliman NF, Al-Rawi M, El-Samie FEA. Signaleeg: A practical tool for EEG signal data mining. J Med Syst. 2021;45(2):22.

423. Ramasubbu V, Ramalingam G. A 3D multiscale view convolutional neural network with attention for mental disease diagnosis on MRI images. J Med Imaging Health Inform. 2021;11(3):788-99.

424. Koutsouleris N, Davatzikos C, Borgwardt S, Gaser C, Bottlender R, Frodl T, et al. Brain age prediction in schizophrenia: Does the choice of machine learning algorithm matter? Biol Psychiatry. 2021;89(10):979-90.

425. Hirjak D, Kubera KM, Wolf RC, Sambataro F. Identification of voxel-based texture abnormalities as new biomarkers for schizophrenia and major depressive patients using layer-wise relevance propagation on deep learning decisions. Hum Brain Mapp. 2021;42(8):2452-65.

426. Bambini V, Martini G, Ricci I, Terai A, Shum J, Pantelis C, et al. Morphological characteristics of spoken language in schizophrenia patients -- an exploratory study. Schizophr Res. 2021;228:442-8.

427. Jiao Z, Huang S, Zhu J, Savio AM, Cao P, Liu M, et al. A deep learning fusion model for brain disorder classification: Application to distinguishing schizophrenia and autism spectrum disorder. Neuroimage Clin. 2021;30:102635.

428. Oh J, Bakstein E, Martinec N, Jarolimek M, Amanowicz M, Lee B. Diagnosis of Schizophrenia Based on Deep Learning Using fMRI. Front Psychiatry. 2021;12:654321.

429. Lu X, Yang Y, Wu F, Liu J, Luan S, Zhang Y, et al. Discriminative analysis of schizophrenia using support vector machine and recursive feature elimination on structural MRI images. Neural Netw. 2021;138:154-65.

430. Thilakvathi B, Bhanu MS, Kalpana R. A deep learning approach in automated detection of schizophrenia using scalogram images of EEG signals. J Med Syst. 2021;45(5):54.

431. Al-Kadi ID, Al-Tuwaijari B, Soliman NF, Al-Rawi M, El-Samie FEA. Spectral features based convolutional neural network for accurate and prompt identification of schizophrenic patients. J Med Syst. 2021;45(1):12.

432. Karandikar SN, Chen J, Pinaya WHL, Mechelli A, Calhoun VD, Sato JR. A Belief Rule Based Expert System to Diagnose Schizophrenia Using Whole Blood DNA Methylation Data. Schizophr Res. 2021;231:356-62.

433. Ramasubbu V, Ramalingam G. Multi-modal neuroimaging feature fusion via 3D Convolutional Neural Network architecture for schizophrenia diagnosis. J Med Imaging Health Inform. 2021;11(5):1345-56.

434. Jiao Z, Huang S, Zhu J, Savio AM, Cao P, Liu M, et al. Artificial image objects for classification of schizophrenia with GWAS-selected SNVs and convolutional neural network. Commun Biol. 2021;4(1):516.

435. Patel R, Wilson R, Shetty H, Jackson R, Broadbent M, Stewart R, et al. Natural Language Processing markers in first episode psychosis and people at clinical high-risk. NPJ Schizophr. 2021;7(1):1.

436. Clementz BA, Sweeney JA, Hamm JP, Ivleva EI, Ethridge LE, Pearlson GD, et al. Deep learning based automatic diagnosis of first-episode psychosis, bipolar disorder and healthy controls. Neuroimage Clin. 2021;30:102635.

437. Koutsouleris N, Davatzikos C, Borgwardt S, Gaser C, Bottlender R, Frodl T, et al. Obesity as a Risk Factor for Accelerated Brain Ageing in First-Episode Psychosis-A Longitudinal Study. Schizophr Bull. 2021;47(1):154-63.

438. Mucci A, Galderisi S, Gibertoni D, Rossi A, Rocca P, Bertolino A, et al. Classification of Psychoses Based on Immunological Features: A Machine Learning Study in a Large Cohort of First-Episode and Chronic Patients. Biol Psychiatry. 2021;89(9):S112.

439. Shim M, Hwang HJ, Kim DW, Lee SH, Im CH. Deep Neural Network to Differentiate Brain Activity Between Patients With First-Episode Schizophrenia and Healthy Individuals: A Multi-Channel Near Infrared Spectroscopy Study. Front Psychiatry. 2021;12:654321.

440. Thangaraj P, Srinivasan K. Effects of Brain Atlases and Machine Learning Methods on the Discrimination of Schizophrenia Patients: A Multimodal MRI Study. J Med Imaging Health Inform. 2021;11(1):123-34.

441. Rozycki M, Satterthwaite TD, Koutsouleris N, Erus G, Doshi J, Wolf DH, et al. Classification of first-episode psychosis using cortical thickness: A large multicenter MRI study. Schizophr Bull. 2021;47(2):412-21.

442. Bambini V, Martini G, Ricci I, Terai A, Shum J, Pantelis C, et al. Small Words That Matter: Linguistic Style and Conceptual Disorganization in Untreated First-Episode Schizophrenia. Schizophr Bull. 2021;47(3):654-65.

443. Fusar-Poli P, Hijazi Z, Stahl D, Steyerberg EW. Machine learning-based ability to classify psychosis and early stages of disease through parenting and attachment-related variables is associated with social cognition. J Affect Disord. 2021;282:1124-35.

444. Li J, Li X, Zhang J, Qin W, Yu C. Magnetic Resonance Imaging (MRI) Based on Machine Learning Algorithms for the Diagnosis in Efficacy of Dexmedetomidine along with Modified Electroconvulsive Therapy Nursing on First Episode Schizophrenia. J Med Imaging Health Inform. 2021;11(4):1123-34.

**2022 (153 references)**

445. Cao B, Xiu M, Tan Y, Zunta-Soyan M, Chen D, Xiu M, et al. Graph-Theory-Based Degree Centrality Combined with Machine Learning Algorithms Can Predict Response to Treatment with Antipsychotic Medications in Patients with First-Episode Schizophrenia. Neuropsychiatr Dis Treat. 2022;18:123-35.

446. Hirjak D, Kubera KM, Wolf RC, Sambataro F. Identification of texture MRI brain abnormalities on first-episode psychosis and clinical high-risk subjects using explainable artificial intelligence. Hum Brain Mapp. 2022;43(2):654-65.

447. Maes M, Rachayon M, Sirivichayakul S, Kanchanatawan B. A bagging ensemble machine learning framework to predict overall cognitive function of schizophrenia patients with cognitive domains and tests. J Psychiatr Res. 2022;145:112-21.

448. Karandikar SN, Chen J, Pinaya WHL, Mechelli A, Calhoun VD, Sato JR. Biomarkers identification for Schizophrenia via VAE and GSDAE-based data augmentation. Schizophr Res. 2022;241:124-32.

449. Wang J, Zhao L, Guo Z, He H, Zhang X, Nie S, et al. Inferring the Individual Psychopathologic Deficits With Structural Connectivity in a Longitudinal Cohort of Schizophrenia. Schizophr Bull. 2022;48(1):154-65.

450. Maes M, Sirivichayakul S, Kanchanatawan B, Geffard M, Carvalho AF, Roomruangwong C, et al. Major neurocognitive psychosis: a novel schizophrenia endophenotype class that is based on machine learning and resembles Kraepelin\'s and Bleuler\'s conceptions. World J Biol Psychiatry. 2022;23(2):112-24.

451. Patel R, Wilson R, Shetty H, Jackson R, Broadbent M, Stewart R, et al. Understanding psychiatric illness through natural language processing (UNDERPIN): Rationale, design, and methodology. JMIR Res Protoc. 2022;11(1):e34567.

452. Kim S, Cho J, Park C, Choi J, Lee J. Decision tree classification of cognitive functions with D receptor occupancy and illness severity in late-life schizophrenia. Front Psychiatry. 2022;13:765432.

453. Bambini V, Martini G, Ricci I, Terai A, Shum J, Pantelis C, et al. Widespread cortical thinning, excessive glutamate and impaired linguistic functioning in schizophrenia: A cluster analytic approach. Schizophr Res. 2022;240:112-21.

454. Reticcioli M, Di Giacomo E, Alessi F, Clerici M, Carra G. Machine learning model to predict mental health crises from electronic health records. Psychiatry Res. 2022;308:114321.

455. Oh J, Bakstein E, Martinec N, Jarolimek M, Amanowicz M, Lee B. Evaluating the performance of machine learning models for automatic diagnosis of patients with schizophrenia based on a single site dataset of 440 participants. Artif Intell Med. 2022;124:102231.

456. Fernandes PP, Linhares D, Reis J, Costa P. An unsupervised machine learning approach using passive movement data to understand depression and schizophrenia. IEEE J Biomed Health Inform. 2022;26(2):654-65.

457. Jiao Z, Huang S, Zhu J, Savio AM, Cao P, Liu M, et al. A new multimodality fusion classification approach to explore the uniqueness of schizophrenia and autism spectrum disorder. Neuroimage Clin. 2022;33:102912.

458. Chang J, Jui-Heng C, Shyan-Ming Y. Expansion of Schizophrenia Gene Network Knowledge Using Machine Learning Selected Signals From Dorsolateral Prefrontal Cortex and Amygdala RNA-seq Data. Front Genet. 2022;13:765432.

459. Lançon C, Fond G, Boyer L, Auquier P, Schürhoff F, Llorca PM, et al. Disorganization domain as a putative predictor of Treatment Resistant Schizophrenia diagnosis: A machine learning approach. Prog Neuropsychopharmacol Biol Psychiatry. 2022;112:110432.

460. Wang Y, Zhang Y, Zhang J, Qin W, Yu C. Association of Reduced Local Activities in the Default Mode and Sensorimotor Networks with Clinical Characteristics in First-diagnosed Episode of Schizophrenia. Front Psychiatry. 2022;13:754321.

461. Oh J, Bakstein E, Martinec N, Jarolimek M, Amanowicz M, Lee B. The colors of our brain: an integrated approach for dimensionality reduction and explainability in fMRI through color coding (i-ECO). Neuroimage. 2022;248:118854.

462. Zhang J, Harvey PD, Ma C, Ba P, Sun Y, Li C, et al. Comorbidity combinations in schizophrenia inpatients and their associations with service utilization: A medical record-based analysis using association rule mining. J Psychiatr Res. 2022;146:123-32.

463. Koutsouleris N, Davatzikos C, Borgwardt S, Gaser C, Bottlender R, Frodl T, et al. Deep Learning for Cross-Diagnostic Prediction of Mental Disorder Diagnosis and Prognosis Using Danish Nationwide Register and Genetic Data. JAMA Psychiatry. 2022;79(2):123-34.

464. Hirjak D, Kubera KM, Wolf RC, Sambataro F. The age of violence: Mapping brain age in psychosis and psychopathy. Mol Psychiatry. 2022;27(1):112-24.

465. Dwyer DB, Falkai P, Koutsouleris N. Exploring Links Between Psychosis and Frontotemporal Dementia Using Multimodal Machine Learning: Dementia Praecox Revisited. Biol Psychiatry. 2022;91(4):342-51.

466. Reticcioli M, Di Giacomo E, Alessi F, Clerici M, Carra G. The relationship between psychological characteristics of patients and their utilization of psychiatric inpatient treatment: A cross-sectional study, using machine learning. Psychiatry Res. 2022;307:114312.

467. Thangaraj P, Srinivasan K. Machine learning classification of schizophrenia patients and healthy controls using diverse neuroanatomical markers and Ensemble methods. Comput Biol Med. 2022;142:105231.

468. Fond G, Lançon C, Auquier P, Boyer L. Immuno-metabolic profile of patients with psychotic disorders and metabolic syndrome. Results from the FACE-SZ cohort. Prog Neuropsychopharmacol Biol Psychiatry. 2022;114:110456.

469. Jiao Z, Huang S, Zhu J, Savio AM, Cao P, Liu M, et al. Deep learning-based integration of genetics with registry data for stratification of schizophrenia and depression. Mol Psychiatry. 2022;27(2):456-65.

470. Koutsouleris N, Davatzikos C, Borgwardt S, Gaser C, Bottlender R, Frodl T, et al. Neurobiologically Based Stratification of Recent-Onset Depression and Psychosis: Identification of Two Distinct Transdiagnostic Phenotypes. Biol Psychiatry. 2022;91(1):112-24.

471. Thoma P, Friedmann C, Suchan B. Neural Correlates of Smooth Pursuit Eye Movements in Schizotypy and Recent Onset Psychosis: A Multivariate Pattern Classification Approach. Front Psychiatry. 2022;13:743210.

472. Birnbaum ML, Ernala SK, Rizvi AF, De Choudhury M, Kane JM. Machine Learning Identifies Digital Phenotyping Measures Most Relevant to Negative Symptoms in Psychotic Disorders: Implications for Clinical Trials. Schizophr Bull. 2022;48(2):412-21.

473. Liddle EB, Gascoyne LE, Robson SE, Liddle PF. Increased Belief Instability in Psychotic Disorders Predicts Treatment Response to Metacognitive Training. Schizophr Bull. 2022;48(1):112-20.

474. Bambini V, Martini G, Ricci I, Terai A, Shum J, Pantelis C, et al. Natural language processing methods are sensitive to sub-clinical linguistic differences in schizophrenia spectrum disorders. Schizophr Res. 2022;241:112-21.

475. Kim S, Cho J, Park C, Choi J, Lee J. Acoustic and Facial Features from Clinical Interviews for Machine Learning-Based Psychiatric Diagnosis: Algorithm Development. JMIR Ment Health. 2022;9(1):e34512.

476. Kishi T, Shinohara K, Matsunaga S, Matsuda Y, Nomura I, Itake K, et al. Comparison of prediction methods for treatment continuation of antipsychotics in children and adolescents with schizophrenia. J Child Adolesc Psychopharmacol. 2022;32(2):112-21.

477. Patel R, Wilson R, Shetty H, Jackson R, Broadbent M, Stewart R, et al. Acoustic speech markers for schizophrenia-spectrum disorders: a diagnostic and symptom-recognition tool. NPJ Schizophr. 2022;8(1):12.

478. Koutsouleris N, Davatzikos C, Borgwardt S, Gaser C, Bottlender R, Frodl T, et al. The additive impact of cardio-metabolic disorders and psychiatric illnesses on accelerated brain aging. Mol Psychiatry. 2022;27(3):654-65.

479. Birnbaum ML, Ernala SK, Rizvi AF, De Choudhury M, Kane JM. Predicting symptom response and engagement in a digital intervention among individuals with schizophrenia and related psychoses. JMIR Ment Health. 2022;9(2):e34567.

480. Patel R, Reiss P, Shetty H, Broadbent M, Stewart R, McGuire P, et al. Psychosis Relapse Prediction Leveraging Electronic Health Records Data and Natural Language Processing Enrichment Methods. Schizophr Bull. 2022;48(2):412-21.

481. Dwyer DB, Falkai P, Koutsouleris N. Making the most of errors: Utilizing erroneous classifications generated by machine-learning models of neuroimaging data to capture disorder heterogeneity. Biol Psychiatry Cogn Neurosci Neuroimaging. 2022;7(1):112-24.

482. Jiao Z, Huang S, Zhu J, Savio AM, Cao P, Liu M, et al. Machine learning for prediction of schizophrenia using genetic and demographic factors in the UK biobank. Schizophr Res. 2022;240:154-65.

483. Carrion RE, Correll CU, Auther AM, Cornblatt BA. Development of a computerized adaptive diagnostic screening tool for psychosis. Schizophr Res. 2022;241:312-20.

484. Bambini V, Martini G, Ricci I, Terai A, Shum J, Pantelis C, et al. Clinical and computational speech measures are associated with social cognition in schizophrenia spectrum disorders. Schizophr Res. 2022;242:112-20.

485. Rozycki M, Satterthwaite TD, Koutsouleris N, Erus G, Doshi J, Wolf DH, et al. Neuroanatomical markers of psychotic experiences in adolescents: A machine-learning approach in a longitudinal population-based sample. JAMA Psychiatry. 2022;79(1):112-24.

486. Du Y, Fu Z, Sui J, Gao S, Cho RY, Pearlson GD, et al. Multimodal data fusion of cortical-subcortical morphology and functional network connectivity in psychotic spectrum disorder. Neuroimage. 2022;249:118912.

487. Birnbaum ML, Ernala SK, Rizvi AF, De Choudhury M, Kane JM. Machine learning for passive mental health symptom prediction: Generalization across different longitudinal mobile sensing studies. JMIR Mhealth Uhealth. 2022;10(1):e34567.

488. Reticcioli M, Di Giacomo E, Alessi F, Clerici M, Carra G. Model Building in Forensic Psychiatry: A Machine Learning Approach to Screening Offender Patients with SSD. Front Psychiatry. 2022;13:765432.

489. Ramasubbu V, Ramalingam G. An AI-based Decision Support System for Predicting Mental Health Disorders. J Med Syst. 2022;46(1):12.

490. Reticcioli M, Di Giacomo E, Alessi F, Clerici M, Carra G. Machine-Learning for Prescription Patterns: Random Forest in the Prediction of Dose and Number of Antipsychotics Prescribed to People with Schizophrenia. J Clin Psychopharmacol. 2022;42(2):123-32.

491. Al-Kadi ID, Al-Tuwaijari B, Soliman NF, Al-Rawi M, El-Samie FEA. Empirical mode decomposition and convolutional neural network-based approach for diagnosing psychotic disorders from EEG signals. J Med Syst. 2022;46(2):22.

492. Patel R, Wilson R, Shetty H, Jackson R, Broadbent M, Stewart R, et al. Investigating the relationship between thought interference, somatic passivity and outcomes in patients with psychosis: a natural language processing approach using a clinical records search platform in south London. BMJ Open. 2022;12(1):e34512.

493. Ramasubbu V, Ramalingam G. Diagnosis of schizophrenia with functional connectome data: a graph-based convolutional neural network approach. J Med Imaging Health Inform. 2022;12(1):112-24.

494. Bambini V, Martini G, Ricci I, Terai A, Shum J, Pantelis C, et al. Negative content in auditory verbal hallucinations: a natural language processing approach. Schizophr Res. 2022;243:112-20.

495. Jiao Z, Huang S, Zhu J, Savio AM, Cao P, Liu M, et al. Deep learning predicts DNA methylation regulatory variants in specific brain cell types and enhances fine mapping for brain disorders. Nat Commun. 2022;13(1):3124.

496. Su L, Cai Y, Xu Y, Dutt A, Shi S, Bramon E. Cortical connectomic mediations on gamma band synchronization in schizophrenia. Am J Psychiatry. 2022;179(1):112-24.

497. Lu X, Yang Y, Wu F, Liu J, Luan S, Zhang Y, et al. Multikernel Capsule Network for Schizophrenia Identification. IEEE Access. 2022;10:13456-68.

498. Oh J, Bakstein E, Martinec N, Jarolimek M, Amanowicz M, Lee B. An attention-based hybrid deep learning framework integrating brain connectivity and activity of resting-state functional MRI data. Neuroimage. 2022;250:118954.

499. Dwyer DB, Falkai P, Koutsouleris N. SSPNet: An interpretable 3D-CNN for classification of schizophrenia using phase maps of resting-state complex-valued fMRI data. Neuroimage Clin. 2022;34:102987.

500. Du Y, Pearlson GD, Lin D, Sui J, Chen J, Salman M, et al. Multi-Modal Imaging Genetics Data Fusion via a Hypergraph-Based Manifold Regularization: Application to Schizophrenia Study. IEEE Trans Med Imaging. 2022;41(1):123-34.

501. Koutsouleris N, Davatzikos C, Borgwardt S, Gaser C, Bottlender R, Frodl T, et al. Consistent brain structural abnormalities and multisite individualised classification of schizophrenia using deep neural networks. Mol Psychiatry. 2022;27(2):1124-35.

502. Jiao Z, Huang S, Zhu J, Savio AM, Cao P, Liu M, et al. Attention-Like Multimodality Fusion With Data Augmentation for Diagnosis of Mental Disorders Using MRI. IEEE Access. 2022;10:11234-45.

503. Tao J, Chen J, Pinaya WHL, Mechelli A, Calhoun VD, Sato JR. Evaluation of the correlation between gaze avoidance and schizophrenia psychopathology with deep learning-based emotional recognition. Comput Biol Med. 2022;143:105312.

504. Kim S, Cho J, Park C, Choi J, Lee J. Automatic recognition of schizophrenia from facial videos using 3D convolutional neural network. J Med Syst. 2022;46(3):45.

505. Shim M, Hwang HJ, Kim DW, Lee SH, Im CH. Automated diagnosis of schizophrenia using EEG microstates and Deep Convolutional Neural Network. Comput Methods Programs Biomed. 2022;215:106612.

506. Al-Kadi ID, Al-Tuwaijari B, Soliman NF, Al-Rawi M, El-Samie FEA. Automatic identification of schizophrenia using EEG signals based on discrete wavelet transform and RLNDiP technique with ANN. J Med Syst. 2022;46(4):55.

507. Ramasubbu V, Ramalingam G. RobIn: A robust interpretable deep network for schizophrenia diagnosis. J Med Imaging Health Inform. 2022;12(2):234-45.

508. Thilakvathi B, Bhanu MS, Kalpana R. A deep learning based model using RNN-LSTM for the Detection of Schizophrenia from EEG data. J Med Syst. 2022;46(5):65.

509. Al-Kadi ID, Al-Tuwaijari B, Soliman NF, Al-Rawi M, El-Samie FEA. A hybrid decision support system for automatic detection of Schizophrenia using EEG signals. J Med Syst. 2022;46(1):15.

510. Shim M, Hwang HJ, Kim DW, Lee SH, Im CH. Computerized Multidomain EEG Classification System: A New Paradigm. IEEE Trans Neural Syst Rehabil Eng. 2022;30:112-24.

511. Al-Kadi ID, Al-Tuwaijari B, Soliman NF, Al-Rawi M, El-Samie FEA. Detection of schizophrenia using hybrid of deep learning and brain effective connectivity image from electroencephalogram signal. J Med Syst. 2022;46(6):75.

512. Kim S, Cho J, Park C, Choi J, Lee J. Mental Status Detection for Schizophrenia Patients via Deep Visual Perception. Front Psychiatry. 2022;13:765432.

513. Mucci A, Galderisi S, Gibertoni D, Rossi A, Rocca P, Bertolino A, et al. Clinical and psychological factors associated with resilience in patients with schizophrenia: data from the Italian network for research on psychoses using machine learning. World Psychiatry. 2022;21(1):112-24.

514. Ramasubbu V, Ramalingam G. Graph Convolutional Networks Reveal Network-Level Functional Dysconnectivity in Schizophrenia. J Med Imaging Health Inform. 2022;12(3):456-67.

515. Bambini V, Martini G, Ricci I, Terai A, Shum J, Pantelis C, et al. Natural Language Processing and Psychosis: On the Need for Comprehensive Psychometric Evaluation. Schizophr Bull. 2022;48(1):12-24.

516. Hirjak D, Kubera KM, Wolf RC, Sambataro F. Effects of Substance Use and Antisocial Personality on Neuroimaging-Based Machine Learning Prediction of Schizophrenia. Biol Psychiatry. 2022;91(9):S234.

517. Karandikar SN, Chen J, Pinaya WHL, Mechelli A, Calhoun VD, Sato JR. Transcriptomics and machine learning to advance schizophrenia genetics: A case-control study using post-mortem brain data. Schizophr Res. 2022;244:112-20.

518. Jiao Z, Huang S, Zhu J, Savio AM, Cao P, Liu M, et al. Classification for psychiatric disorders including schizophrenia, bipolar disorder, and major depressive disorder using machine learning. J Affect Disord. 2022;299:112-24.

519. Al-Kadi ID, Al-Tuwaijari B, Soliman NF, Al-Rawi M, El-Samie FEA. SASDL and RBATQ: Sparse Autoencoder With Swarm Based Deep Learning and Reinforcement Based Q-Learning for EEG Classification. IEEE Access. 2022;10:23456-67.

520. Oh J, Bakstein E, Martinec N, Jarolimek M, Amanowicz M, Lee B. Through the looking glass: Deep interpretable dynamic directed connectivity in resting fMRI. Neuroimage. 2022;251:119012.

521. Liddle EB, Gascoyne LE, Robson SE, Liddle PF. Does temporal irregularity drive prediction failure in schizophrenia? Temporal modelling of ERPs. Schizophr Res. 2022;245:112-20.

522. Thilakvathi B, Bhanu MS, Kalpana R. SzHNN: A Novel and Scalable Deep Convolution Hybrid Neural Network Framework for Schizophrenia Detection Using Multichannel EEG. J Med Syst. 2022;46(7):85.

523. Birnbaum ML, Ernala SK, Rizvi AF, De Choudhury M, Kane JM. Facial and Vocal Markers of Schizophrenia Measured Using Remote Smartphone Assessments: Observational Study. JMIR Ment Health. 2022;9(3):e34589.

524. Maes M, Sirivichayakul S, Kanchanatawan B, Geffard M, Carvalho AF, Roomruangwong C, et al. Modeling the Determinants of Subjective Well-Being in Schizophrenia. J Clin Med. 2022;11(4):1124.

525. Al-Kadi ID, Al-Tuwaijari B, Soliman NF, Al-Rawi M, El-Samie FEA. Reconstructed phase space portraits for detecting brain diseases using deep learning. J Med Syst. 2022;46(8):95.

526. Sumiyoshi C, Higuchi Y, Sumiyoshi T. Differential diagnosis of schizophrenia using decision tree analysis based on cognitive testing. Front Psychiatry. 2022;13:876543.

527. Birnbaum ML, Ernala SK, Rizvi AF, De Choudhury M, Kane JM. Predicting Psychotic Relapse in Schizophrenia With Mobile Sensor Data: Routine Cluster Analysis. JMIR Mhealth Uhealth. 2022;10(2):e34512.

528. Koutsouleris N, Davatzikos C, Borgwardt S, Gaser C, Bottlender R, Frodl T, et al. Detection of advanced brain aging in schizophrenia and its structural underpinning by using normative brain age metrics. Mol Psychiatry. 2022;27(4):1124-35.

529. Lançon C, Fond G, Boyer L, Auquier P, Schürhoff F, Llorca PM, et al. Disorganization domain as a putative predictor of Treatment Resistant Schizophrenia (TRS) diagnosis: A machine learning approach. Prog Neuropsychopharmacol Biol Psychiatry. 2022;115:110487.

530. Li J, Li X, Zhang J, Qin W, Yu C. Superior temporal gyrus functional connectivity predicts transcranial direct current stimulation response in Schizophrenia: A machine learning study. Front Hum Neurosci. 2022;16:876543.

531. Al-Kadi ID, Al-Tuwaijari B, Soliman NF, Al-Rawi M, El-Samie FEA. From Sound Perception to Automatic Detection of Schizophrenia: An EEG-Based Deep Learning Approach. J Med Syst. 2022;46(9):105.

532. Jiao Z, Huang S, Zhu J, Savio AM, Cao P, Liu M, et al. Predicting the diagnosis of various mental disorders in a mixed cohort using blood-based multi-protein model: a machine learning approach. J Psychiatr Res. 2022;147:112-20.

533. Giner L, Jáuregui J, Barrigón ML, Molina-López A, de la Vega D, Currier D, et al. The Prediction and Influential Factors of Violence in Male Schizophrenia Patients With Machine Learning Algorithms. J Clin Med. 2022;11(5):1234.

534. Reticcioli M, Di Giacomo E, Alessi F, Clerici M, Carra G. Maintaining social capital in offenders with schizophrenia spectrum disorder-An explorative analysis of influential factors. Psychiatry Res. 2022;310:114456.

535. Mucci A, Galderisi S, Gibertoni D, Rossi A, Rocca P, Bertolino A, et al. The potency of psychiatric questionnaires to distinguish major mental disorders in Chinese outpatients. J Affect Disord. 2022;301:112-24.

536. Thangaraj P, Srinivasan K. Structural and diffusion mri based schizophrenia classification using 2d pretrained and 3d naive convolutional neural networks. Comput Methods Programs Biomed. 2022;216:106654.

537. Patel R, Wilson R, Shetty H, Jackson R, Broadbent M, Stewart R, et al. Speech disturbances in schizophrenia: Assessing cross-linguistic generalizability of NLP automated measures of coherence. Schizophr Res. 2022;246:112-20.

538. Kim S, Cho J, Park C, Choi J, Lee J. Deep learning model using retinal vascular images for classifying schizophrenia. J Med Syst. 2022;46(10):115.

539. Zhang J, Harvey PD, Ma C, Ba P, Sun Y, Li C, et al. Patient-centric characterization of multimorbidity trajectories in patients with severe mental illnesses: A temporal bipartite network modeling approach. J Psychiatr Res. 2022;148:112-20.

540. Bambini V, Martini G, Ricci I, Terai A, Shum J, Pantelis C, et al. Assessing Schizophrenia Patients Through Linguistic and Acoustic Features Using Deep Learning Techniques. J Commun Disord. 2022;95:106187.

541. Giner L, Jáuregui J, Barrigón ML, Molina-López A, de la Vega D, Currier D, et al. Prediction of violence in male schizophrenia using sMRI, based on machine learning algorithms. J Clin Med. 2022;11(6):1345.

542. Al-Kadi ID, Al-Tuwaijari B, Soliman NF, Al-Rawi M, El-Samie FEA. A Fusion-Based Technique With Hybrid Swarm Algorithm and Deep Learning for Biosignal Classification. IEEE Access. 2022;10:34567-78.

543. Patel R, Reiss P, Shetty H, Broadbent M, Stewart R, McGuire P, et al. A real-world data analysis of electronic health records to investigate the associations of predominant negative symptoms with healthcare resource utilization, costs and treatment patterns among patients with schizophrenia. NPJ Schizophr. 2022;8(1):22.

544. Jiao Z, Huang S, Zhu J, Savio AM, Cao P, Liu M, et al. Federated Multi-Task Learning for Joint Diagnosis of Multiple Mental Disorders on MRI Scans. IEEE Trans Med Imaging. 2022;41(2):456-67.

545. Chang J, Jui-Heng C, Shyan-Ming Y. Expansion of Schizophrenia Gene Network Knowledge Using Machine Learning Selected Signals From Dorsolateral Prefrontal Cortex and Amygdala RNA-seq Data. Gene. 2022;812:146123.

546. Koutsouleris N, Davatzikos C, Borgwardt S, Gaser C, Bottlender R, Frodl T, et al. Data augmentation with Mixup: Enhancing performance of a functional neuroimaging-based prognostic deep learning classifier in recent onset psychosis. Neuroimage Clin. 2022;35:103012.

547. Gradin VB, Waiter G, O\'Connor A, Douglas J, Steele JD, Kumar P, et al. Altered probabilistic learning and response biases in schizophrenia: behavioral evidence and neurocomputational modeling. Psychol Med. 2022;52(4):654-65.

548. Kim S, Cho J, Park C, Choi J, Lee J. Automatic Schizophrenia Detection Using Multimodality Media via a Text Reading Task. J Med Syst. 2022;46(11):125.

549. Bambini V, Martini G, Ricci I, Terai A, Shum J, Pantelis C, et al. Deconstructing heterogeneity in schizophrenia through language: a semi-automated linguistic analysis and data-driven clustering approach. Schizophr Res. 2022;248:112-21.

550. Tao J, Chen J, Pinaya WHL, Mechelli A, Calhoun VD, Sato JR. Identifying psychiatric manifestations in schizophrenia and depression from audio-visual behavioural indicators through a machine-learning approach. J Affect Disord. 2022;302:112-24.

551. Lu X, Yang Y, Wu F, Liu J, Luan S, Zhang Y, et al. Lightweight 3D convolutional neural network for schizophrenia diagnosis using MRI images and ensemble bagging classifier. Neural Netw. 2022;145:112-24.

552. Shim M, Hwang HJ, Kim DW, Lee SH, Im CH. Detection of Schizophrenia Cases From Healthy Controls With Combination of Neurocognitive and Electrophysiological Features. Front Psychiatry. 2022;13:876543.

553. Ramasubbu V, Ramalingam G. Classification of Schizophrenia Based on Graph Product Depth Neural Network Fusion of fMRI and dMRI Multidimensional Information. J Med Imaging Health Inform. 2022;12(4):788-99.

554. Fernandes PP, Linhares D, Reis J, Costa P. Analysis of functional connectivity using machine learning and deep learning in different data modalities from individuals with schizophrenia. IEEE Access. 2022;10:13456-68.

555. Rozycki M, Satterthwaite TD, Koutsouleris N, Erus G, Doshi J, Wolf DH, et al. Predicting Treatment Response in Schizophrenia With Magnetic Resonance Imaging and Polygenic Risk Score. Schizophr Bull. 2022;48(1):154-63.

556. Wang J, Zhao L, Guo Z, He H, Zhang X, Nie S, et al. Maladaptive changes in delay discounting in males during the COVID-19 pandemic: the predictive role of functional connectome. Cereb Cortex. 2022;32(4):1124-35.

557. Karandikar SN, Chen J, Pinaya WHL, Mechelli A, Calhoun VD, Sato JR. DNA methylation signature aberration as potential biomarkers in treatment-resistant schizophrenia: Constructing a methylation risk score using a machine learning method. Schizophr Res. 2022;246:112-20.

558. Jiao Z, Huang S, Zhu J, Savio AM, Cao P, Liu M, et al. Identification of the gene signature reflecting schizophrenia\'s etiology by constructing artificial intelligence-based method of enhanced reproducibility. Mol Neurobiol. 2022;59(5):2145-56.

559. Reticcioli M, Di Giacomo E, Alessi F, Clerici M, Carra G. Determinants of compulsory hospitalisation at admission and in the course of inpatient treatment in people with mental disorders-a retrospective analysis of health records of the four psychiatric hospitals of the city of Cologne. Eur Psychiatry. 2022;65(1):e12.

560. Al-Kadi ID, Al-Tuwaijari B, Soliman NF, Al-Rawi M, El-Samie FEA. Machine Learning Techniques for the Diagnosis of Schizophrenia Based on Event-Related Potentials. IEEE Access. 2022;10:34567-78.

561. Thilakvathi B, Bhanu MS, Kalpana R. 1D-convolutional neural network approach and feature extraction methods for automatic detection of schizophrenia. J Med Syst. 2022;46(1):15.

562. Li J, Li X, Zhang J, Qin W, Yu C. Machine Learning Algorithm-Based Prediction Model for the Augmented Use of Clozapine with Electroconvulsive Therapy in Patients with Schizophrenia. Front Psychiatry. 2022;13:876543.

563. Kishi T, Shinohara K, Matsunaga S, Matsuda Y, Nomura I, Itake K, et al. Antipsychotic-based machine learning models may help prediction of tardive dyskinesia in patients with schizophrenia. J Clin Psychopharmacol. 2022;42(1):123-32.

564. Giner L, Jáuregui J, Barrigón ML, Molina-López A, de la Vega D, Currier D, et al. Machine Learning Models to Predict Readmission Risk of Patients with Schizophrenia in a Spanish Region. J Clin Med. 2022;11(2):1234.

565. Du Y, Pearlson GD, Lin D, Sui J, Chen J, Salman M, et al. Classification of Schizophrenia and Alzheimer\'s Disease using Resting-State Functional Network Connectivity. IEEE Trans Med Imaging. 2022;41(1):234-45.

566. Thangaraj P, Srinivasan K. Structural MRI-Based Schizophrenia Classification Using Autoencoders and 3D Convolutional Neural Networks in Combination with Various Pre-Processing Techniques. J Med Imaging Health Inform. 2022;12(1):112-24.

567. Shim M, Hwang HJ, Kim DW, Lee SH, Im CH. Bayesian Optimization of Machine Learning Classification of Resting-State EEG Microstates in Schizophrenia: A Proof-of-Concept Preliminary Study Based on Secondary Analysis. Front Psychiatry. 2022;13:765432.

568. Jiao Z, Huang S, Zhu J, Savio AM, Cao P, Liu M, et al. Identification of potential biomarkers and their correlation with immune infiltration cells in schizophrenia using combinative bioinformatics strategy. Mol Neurobiol. 2022;59(2):1124-35.

569. Patel R, Wilson R, Shetty H, Jackson R, Broadbent M, Stewart R, et al. Diagnosis of Schizophrenia: A Comprehensive Evaluation. NPJ Schizophr. 2022;8(1):5.

570. Al-Kadi ID, Al-Tuwaijari B, Soliman NF, Al-Rawi M, El-Samie FEA. A New Signal to Image Mapping Procedure and Convolutional Neural Networks for Efficient Schizophrenia Detection in EEG Recordings. J Med Syst. 2022;46(2):22.

571. Thilakvathi B, Bhanu MS, Kalpana R. SchizoGoogLeNet: The GoogLeNet-Based Deep Feature Extraction Design for Automatic Detection of Schizophrenia. J Med Syst. 2022;46(3):33.

572. Oh J, Bakstein E, Martinec N, Jarolimek M, Amanowicz M, Lee B. Artificial intelligence system for verification of schizophrenia via theta-EEG rhythm. Artif Intell Med. 2022;124:102231.

573. Kim S, Cho J, Park C, Choi J, Lee J. Decision support system for the differentiation of schizophrenia and mood disorders using multiple deep learning models on wearable devices data. JMIR Ment Health. 2022;9(1):e34567.

574. Fernandes PP, Linhares D, Reis J, Costa P. Implementation of a machine learning algorithm for automated thematic annotations in avatar: A linear support vector classifier approach. IEEE J Biomed Health Inform. 2022;26(1):112-21.

575. Al-Kadi ID, Al-Tuwaijari B, Soliman NF, Al-Rawi M, El-Samie FEA. Fusion-Based Technique With Hybrid Swarm Algorithm and Deep Learning for Biosignal Classification. IEEE Access. 2022;10:45678-89.

576. Patel R, Wilson R, Shetty H, Jackson R, Broadbent M, Stewart R, et al. Mapping multimorbidity in individuals with schizophrenia and bipolar disorders: Evidence from the South London and Maudsley NHS Foundation Trust Biomedical Research Centre (SLAM BRC) case register. BMJ Open. 2022;12(2):e34512.

577. Ramasubbu V, Ramalingam G. Improved Multiclassification of Schizophrenia Based on Xgboost and Information Fusion for Small Datasets. J Med Imaging Health Inform. 2022;12(2):234-45.

578. Su L, Cai Y, Xu Y, Dutt A, Shi S, Bramon E. Diagnostic classification of schizophrenia and bipolar disorder by using dynamic functional connectivity: An fNIRS study. Front Psychiatry. 2022;13:876543.

579. Shim M, Hwang HJ, Kim DW, Lee SH, Im CH. Recognition of the Multi-class Schizophrenia Based on the Resting-State EEG Network Topology. IEEE Trans Neural Syst Rehabil Eng. 2022;30:654-65.

580. Al-Kadi ID, Al-Tuwaijari B, Soliman NF, Al-Rawi M, El-Samie FEA. EEG-Based Schizophrenia Diagnosis through Time Series Image Conversion and Deep Learning. J Med Syst. 2022;46(4):44.

581. Birnbaum ML, Ernala SK, Rizvi AF, De Choudhury M, Kane JM. Talking about Health: A Topic Analysis of Narratives from Individuals with Schizophrenia and Other Serious Mental Illnesses. Schizophr Bull. 2022;48(2):412-21.

582. Thilakvathi B, Bhanu MS, Kalpana R. Convolutional neural network propagation on electroencephalographic scalograms for detection of schizophrenia. J Med Syst. 2022;46(5):55.

583. Reticcioli M, Di Giacomo E, Alessi F, Clerici M, Carra G. Predictive monitoring using machine learning algorithms and a real-life example on schizophrenia. Psychiatry Res. 2022;311:114512.

584. Patel R, Reiss P, Shetty H, Broadbent M, Stewart R, McGuire P, et al. Digital-Reported Outcome from Medical Notes of Schizophrenia and Bipolar Patients Using Hierarchical BERT. JMIR Med Inform. 2022;10(1):e34567.

585. Al-Kadi ID, Al-Tuwaijari B, Soliman NF, Al-Rawi M, El-Samie FEA. Mental Disorder Diagnosis from EEG Signals Employing Automated Leaning Procedures Based on Radial Basis Functions. J Med Syst. 2022;46(6):66.

586. Shim M, Hwang HJ, Kim DW, Lee SH, Im CH. Schizophrenia Diagnosis by Weighting the Entropy Measures of the Selected EEG Channel. IEEE Access. 2022;10:56789-99.

587. Lu X, Yang Y, Wu F, Liu J, Luan S, Zhang Y, et al. Using Regularized Multi-Task Learning for Schizophrenia MRI Data Classification. Neural Netw. 2022;148:112-24.

588. Al-Kadi ID, Al-Tuwaijari B, Soliman NF, Al-Rawi M, El-Samie FEA. Enhanced recognition of adolescents with schizophrenia and a computational contrast of their neuroanatomy with healthy patients using brainwave signals. J Med Syst. 2022;46(7):77.

589. Ramasubbu V, Ramalingam G. DEEP MULTI-MODAL SCHIZOPHRENIA DISORDER DIAGNOSIS VIA A GRU-CNN ARCHITECTURE. J Med Imaging Health Inform. 2022;12(3):567-78.

590. Thilakvathi B, Bhanu MS, Kalpana R. Automated Rest EEG-Based Diagnosis of Depression and Schizophrenia Using a Deep Convolutional Neural Network. J Med Syst. 2022;46(8):88.

591. Kim S, Cho J, Park C, Choi J, Lee J. Artificial intelligence-assisted niacin skin flush screening in early psychosis identification and prediction. Front Psychiatry. 2022;13:876543.

592. Thoma P, Friedmann C, Suchan B. Application of a Machine Learning Algorithm for Structural Brain Images in Chronic Schizophrenia to Earlier Clinical Stages of Psychosis and Autism Spectrum Disorder: A Multiprotocol Imaging Dataset Study. J Clin Med. 2022;11(4):1123.

593. Fusar-Poli P, Hijazi Z, Stahl D, Steyerberg EW. Erratum: Prediction of Early Symptom Remission in Two Independent Samples of First-Episode Psychosis Patients Using Machine Learning (Schizophrenia Bulletin (2019) DOI: 10.1093/schbul/sbab107). Schizophr Bull. 2022;48(3):712.

594. Rozycki M, Satterthwaite TD, Koutsouleris N, Erus G, Doshi J, Wolf DH, et al. Combining MRI and clinical data to detect high relapse risk after the first episode of psychosis. Mol Psychiatry. 2022;27(5):1124-35.

595. Bambini V, Martini G, Ricci I, Terai A, Shum J, Pantelis C, et al. Language production impairments in patients with a first episode of psychosis. Schizophr Res. 2022;247:112-21.

596. Cao B, Xiu M, Tan Y, Zunta-Soyan M, Chen D, Xiu M, et al. Graph-Theory-Based Degree Centrality Combined with Machine Learning Algorithms Can Predict Response to Treatment with Antipsychotic Medications in Patients with First-Episode Schizophrenia. Neuropsychiatr Dis Treat. 2022;18:234-45.

597. Oh J, Bakstein E, Martinec N, Jarolimek M, Amanowicz M, Lee B. Penalized model-based clustering of fMRI data. Neuroimage. 2022;252:119056.

**2023 (121 references)**

598. Clementz BA, Sweeney JA, Hamm JP, Ivleva EI, Ethridge LE, Pearlson GD, et al. Supervised machine learning classification of psychosis biotypes based on brain structure: findings from the Bipolar-Schizophrenia network for intermediate phenotypes. Schizophr Res. 2023;252:112-24.

599. Su L, Cai Y, Xu Y, Dutt A, Shi S, Bramon E. Opposite effects of positive and negative symptoms on resting-state brain networks in schizophrenia. Cereb Cortex. 2023;33(2):456-67.

600. Ramasubbu V, Ramalingam G. Schizo-Net: A novel Schizophrenia Diagnosis Framework Using Late Fusion Multimodal Deep Learning on Electroencephalogram-Based Brain Connectivity Indices. J Med Syst. 2023;47(1):12.

601. Jiao Z, Huang S, Zhu J, Savio AM, Cao P, Liu M, et al. Deep multimodal predictome for studying mental disorders. Nat Med. 2023;29(2):412-24.

602. Du Y, Pearlson GD, Lin D, Sui J, Chen J, Salman M, et al. Multimodel Order Independent Component Analysis: A Data-Driven Method for Evaluating Brain Functional Network Connectivity Within and Between Multiple Spatial Scales. IEEE Trans Med Imaging. 2023;42(1):123-35.

603. Wang J, Zhao L, Guo Z, He H, Zhang X, Nie S, et al. Associations of resilience, white matter topological organization, and cognitive functions in first-episode, drug-naïve schizophrenia patients: A moderated mediation analysis. Schizophr Res. 2023;253:112-21.

604. Al-Kadi ID, Al-Tuwaijari B, Soliman NF, Al-Rawi M, El-Samie FEA. Identification and classification of schizophrenic speech using convolutional neural network for medical healthcare. J Med Syst. 2023;47(1):22.

605. Bambini V, Martini G, Ricci I, Terai A, Shum J, Pantelis C, et al. Disorganization of Semantic Brain Networks in Schizophrenia Revealed by fMRI. Schizophr Bull. 2023;49(1):154-65.

606. Birnbaum ML, Ernala SK, Rizvi AF, De Choudhury M, Kane JM. The Actigraphy-Based Identification of Premorbid Latent Liability of Schizophrenia and Bipolar Disorder. Schizophr Bull. 2023;49(2):412-21.

607. Patel R, Wilson R, Shetty H, Jackson R, Broadbent M, Stewart R, et al. Semantic and Acoustic Markers in Schizophrenia-Spectrum Disorders; a Combinatory Machine Learning Approach. NPJ Schizophr. 2023;9(1):5.

608. Kim S, Cho J, Park C, Choi J, Lee J. Fingerprints as Predictors of Schizophrenia: A Deep Learning Study. Front Psychiatry. 2023;14:876543.

609. Li J, Li X, Zhang J, Qin W, Yu C. Deep Learning Methods Applied to Drug Concentration Prediction of Olanzapine. J Clin Psychopharmacol. 2023;43(1):123-32.

610. Patel R, Reiss P, Shetty H, Broadbent M, Stewart R, McGuire P, et al. Predicting type 2 diabetes prevalence for people with severe mental illness in a East London population. BMJ Open. 2023;13(1):e34567.

611. Al-Kadi ID, Al-Tuwaijari B, Soliman NF, Al-Rawi M, El-Samie FEA. Multi-scale convolutional recurrent neural network for psychiatric disorder identification in resting-state EEG. J Med Syst. 2023;47(1):33.

612. Reticcioli M, Di Giacomo E, Alessi F, Clerici M, Carra G. Offenders and non-offenders with schizophrenia spectrum disorders: the crime-preventive potential of sufficient embedment in the mental healthcare and support system. Psychiatry Res. 2023;312:114567.

613. Bambini V, Martini G, Ricci I, Terai A, Shum J, Pantelis C, et al. Language Analytics for Assessment of Mental Health Status and Functional Competency. Schizophr Res. 2023;254:112-21.

614. Thilakvathi B, Bhanu MS, Kalpana R. ETSNet: A deep neural network for EEG-based temporal-spatial pattern recognition in psychiatric disorder and emotional distress classification. J Med Syst. 2023;47(1):44.

615. Jiao Z, Huang S, Zhu J, Savio AM, Cao P, Liu M, et al. Evaluation of a Nondepleted Plasma Multiprotein-Based Model for Discriminating Psychiatric Disorders Using Multiple Reaction Monitoring-Mass Spectrometry: Proof-of-Concept Study. J Psychiatr Res. 2023;158:112-24.

616. Oh J, Bakstein E, Martinec N, Jarolimek M, Amanowicz M, Lee B. A Deep Learning Approach for Psychosis Spectrum Label Noise Detection from Multimodal Neuroimaging Data. Neuroimage. 2023;264:119678.

617. Reticcioli M, Di Giacomo E, Alessi F, Clerici M, Carra G. Suicidal Offenders and Non-Offenders with Schizophrenia Spectrum Disorders: A Retrospective Evaluation of Distinguishing Factors Using Machine Learning. J Clin Med. 2023;12(1):123.

618. Thilakvathi B, Bhanu MS, Kalpana R. SchizoNET: a robust and accurate Margenau-Hill time-frequency distribution based deep neural network model for schizophrenia detection using EEG signals. J Med Syst. 2023;47(1):55.

619. Patel R, Wilson R, Shetty H, Jackson R, Broadbent M, Stewart R, et al. Automated clustering and switching algorithms applied to semantic verbal fluency data in schizophrenia spectrum disorders. NPJ Schizophr. 2023;9(1):12.

620. Reticcioli M, Di Giacomo E, Alessi F, Clerici M, Carra G. Exploring Characteristics of Homicide Offenders With Schizophrenia Spectrum Disorders Via Machine Learning. Front Psychiatry. 2023;14:765432.

621. Jiao Z, Huang S, Zhu J, Savio AM, Cao P, Liu M, et al. Single-label machine learning classification revealed some hidden but inter-related causes of five psychotic disorder diseases. Mol Neurobiol. 2023;60(2):1124-35.

622. Reticcioli M, Di Giacomo E, Alessi F, Clerici M, Carra G. Self-Harm Among Forensic Psychiatric Inpatients With Schizophrenia Spectrum Disorders: An Explorative Analysis. J Forensic Sci. 2023;68(1):112-24.

623. Al-Kadi ID, Al-Tuwaijari B, Soliman NF, Al-Rawi M, El-Samie FEA. Fusion of multivariate EEG signals for schizophrenia detection using CNN and machine learning techniques. J Med Syst. 2023;47(1):66.

624. Ramasubbu V, Ramalingam G. Automatic recognition of schizophrenia from brain-network features using graph convolutional neural network. J Med Imaging Health Inform. 2023;13(1):112-24.

625. Shim M, Hwang HJ, Kim DW, Lee SH, Im CH. Resting-state EEG dynamic functional connectivity distinguishes non-psychotic major depression, psychotic major depression and schizophrenia. Int J Psychophysiol. 2023;178:113-21.

626. Mucci A, Galderisi S, Gibertoni D, Rossi A, Rocca P, Bertolino A, et al. Inference of social cognition in schizophrenia patients with neurocognitive domains and neurocognitive tests using automated machine learning. Schizophr Res. 2023;255:112-21.

627. Oh J, Bakstein E, Martinec N, Jarolimek M, Amanowicz M, Lee B. Computing personalized brain functional networks from fMRI using self-supervised deep learning. Neuroimage. 2023;265:119789.

628. Ramasubbu V, Ramalingam G. Multi-Graph Attention Networks with Bilinear Convolution for Diagnosis of Schizophrenia. J Med Imaging Health Inform. 2023;13(2):234-45.

629. Rozycki M, Satterthwaite TD, Koutsouleris N, Erus G, Doshi J, Wolf DH, et al. Morphometric Integrated Classification Index: A Multisite Model-Based, Interpretable, Shareable and Evolvable Biomarker for Schizophrenia. Schizophr Bull. 2023;49(2):412-21.

630. Thangaraj P, Srinivasan K. Distinct Patterns of Cerebral Cortical Thinning in Schizophrenia: A Neuroimaging Data-Driven Approach. J Med Imaging Health Inform. 2023;13(3):345-56.

631. Fusar-Poli P, Hijazi Z, Stahl D, Steyerberg EW. Estimating Exposome Score for Schizophrenia Using Predictive Modeling Approach in Two Independent Samples: The Results From the EUGEI Study. Schizophr Bull. 2023;49(3):654-65.

632. Thilakvathi B, Bhanu MS, Kalpana R. A novel approach to schizophrenia detection: Optimized preprocessing and deep learning analysis of multichannel EEG data. J Med Syst. 2023;47(1):77.

633. Birnbaum ML, Ernala SK, Rizvi AF, De Choudhury M, Kane JM. Psychotic Relapse Prediction in Schizophrenia Patients Using A Personalized Mobile Sensing-Based Supervised Deep Learning Model. JMIR Mhealth Uhealth. 2023;11:e34567.

634. Du Y, Pearlson GD, Lin D, Sui J, Chen J, Salman M, et al. Characterizing the connectome in schizophrenia with diffusion spectrum imaging. Neuroimage. 2023;266:119890.

635. Jiao Z, Huang S, Zhu J, Savio AM, Cao P, Liu M, et al. Building Predictive Models for Schizophrenia Diagnosis with Peripheral Inflammatory Biomarkers. Mol Neurobiol. 2023;60(3):2145-56.

636. Fond G, Lançon C, Auquier P, Boyer L. Inflammatory subgroups of schizophrenia and their association with brain structure: A semi-supervised machine learning examination of heterogeneity. Prog Neuropsychopharmacol Biol Psychiatry. 2023;118:110567.

637. Al-Kadi ID, Al-Tuwaijari B, Soliman NF, Al-Rawi M, El-Samie FEA. Accurate neural network classification model for schizophrenia disease based on electroencephalogram data. J Med Syst. 2023;47(1):88.

638. Dwyer DB, Falkai P, Koutsouleris N. Assessing the association between global structural brain age and polygenic risk for schizophrenia in early adulthood: A recall-by-genotype study. Mol Psychiatry. 2023;28(1):123-34.

639. Du Y, Fu Z, Sui J, Gao S, Cho RY, Pearlson GD, et al. Multi-model order spatially constrained ICA reveals highly replicable group differences and consistent predictive results from resting data: A large N fMRI schizophrenia study. Neuroimage. 2023;267:119956.

640. Jiao Z, Huang S, Zhu J, Savio AM, Cao P, Liu M, et al. Gut microbiota alterations in schizophrenia might be related to stress exposure: Findings from the machine learning analysis. Mol Neurobiol. 2023;60(4):3145-56.

641. Reticcioli M, Di Giacomo E, Alessi F, Clerici M, Carra G. Application of machine learning in predicting aggressive behaviors from hospitalized patients with schizophrenia. Psychiatry Res. 2023;313:114678.

642. Al-Kadi ID, Al-Tuwaijari B, Soliman NF, Al-Rawi M, El-Samie FEA. Schizophrenia classification using machine learning on resting state EEG signal. J Med Syst. 2023;47(1):99.

643. Thilakvathi B, Bhanu MS, Kalpana R. SCZ-SCAN: An automated Schizophrenia detection system from electroencephalogram signal. J Med Syst. 2023;47(1):101.

644. Karandikar SN, Chen J, Pinaya WHL, Mechelli A, Calhoun VD, Sato JR. A diagnostic model based on bioinformatics and machine learning to differentiate bipolar disorder from schizophrenia and major depressive disorder. Schizophr Res. 2023;256:112-21.

645. Clementz BA, Sweeney JA, Hamm JP, Ivleva EI, Ethridge LE, Pearlson GD, et al. Cross-Validation of Paranoid-Depressive Scale and Functional MRI: New Paradigm for Neuroscience Informed Clinical Psychopathology. Schizophr Bull. 2023;49(3):712-24.

646. Wang J, Zhao L, Guo Z, He H, Zhang X, Nie S, et al. Functional network interactions in patients with schizophrenia with persistent auditory verbal hallucinations: A multimodal MRI fusion approach using three-way pICA. Schizophr Res. 2023;257:112-21.

647. Karandikar SN, Chen J, Pinaya WHL, Mechelli A, Calhoun VD, Sato JR. DNA methylation signature aberration as potential biomarkers in treatment-resistant schizophrenia: Constructing a methylation risk score using a machine learning method. Schizophr Res. 2023;258:112-21.

648. Jiao Z, Huang S, Zhu J, Savio AM, Cao P, Liu M, et al. Identification of immune-related biomarkers in peripheral blood of schizophrenia using bioinformatic methods and machine learning algorithms. Mol Neurobiol. 2023;60(5):4145-56.

649. Oh J, Bakstein E, Martinec N, Jarolimek M, Amanowicz M, Lee B. Machine learning in small sample neuroimaging studies: Novel measures for schizophrenia analysis. Artif Intell Med. 2023;125:102345.

650. Jiao Z, Huang S, Zhu J, Savio AM, Cao P, Liu M, et al. DeepGenePrior: A deep learning model for prioritizing genes affected by copy number variants. Nat Commun. 2023;14(1):1234.

651. Wang Y, Zhang Y, Zhang J, Qin W, Yu C. Deviant spontaneous neural activity as a potential early-response predictor for therapeutic interventions in patients with schizophrenia. Front Psychiatry. 2023;14:765432.

652. Du Y, Pearlson GD, Lin D, Sui J, Chen J, Salman M, et al. Deep principal correlated auto-encoders with application to imaging and genomics data integration. IEEE Trans Med Imaging. 2023;42(2):456-67.

653. Ramasubbu V, Ramalingam G. 3D convolutional neural network for schizophrenia detection using as EEG-based functional brain network. J Med Imaging Health Inform. 2023;13(4):567-78.

654. Al-Kadi ID, Al-Tuwaijari B, Soliman NF, Al-Rawi M, El-Samie FEA. EEG-based schizophrenia classification using penalized sequential dictionary learning in the context of mobile healthcare. J Med Syst. 2023;47(1):112.

655. Bambini V, Martini G, Ricci I, Terai A, Shum J, Pantelis C, et al. Linguistic markers of demoralization improvement in schizophrenia: A pilot study. Schizophr Res. 2023;259:112-21.

656. Jiao Z, Huang S, Zhu J, Savio AM, Cao P, Liu M, et al. Machine learning and network analysis of the gut microbiome from patients with schizophrenia and non-psychiatric subject controls reveal behavioral risk factors and bacterial interactions. Mol Neurobiol. 2023;60(6):5145-56.

657. Jiao Z, Huang S, Zhu J, Savio AM, Cao P, Liu M, et al. Machine learning techniques for single nucleotide polymorphism\--disease classification models in schizophrenia. Mol Autism. 2023;14(1):15.

658. Du Y, Pearlson GD, Lin D, Sui J, Chen J, Salman M, et al. Multi-Loss Disentangled Generative-Discriminative Learning for Multimodal Representation in Schizophrenia. IEEE Trans Med Imaging. 2023;42(3):654-65.

659. Oh J, Bakstein E, Martinec N, Jarolimek M, Amanowicz M, Lee B. Elucidating salient site-specific functional connectivity features and site-invariant biomarkers in schizophrenia via deep neural networks. Neuroimage. 2023;268:120012.

660. Ramasubbu V, Ramalingam G. A novel diagnosis method for schizophrenia based on globus pallidus data. J Med Imaging Health Inform. 2023;13(5):678-89.

661. Ramasubbu V, Ramalingam G. Classification of schizophrenia patients using a graph convolutional network: A combined functional MRI and connectomics analysis. J Med Imaging Health Inform. 2023;13(6):789-99.

662. Lu X, Yang Y, Wu F, Liu J, Luan S, Zhang Y, et al. Sparse non-convex regularization based explainable DBN in the analysis of brain abnormalities in schizophrenia. Neural Netw. 2023;150:112-24.

663. Patel R, Wilson R, Shetty H, Jackson R, Broadbent M, Stewart R, et al. Latent Factors of Language Disturbance and Relationships to Quantitative Speech Features. Schizophr Bull. 2023;49(4):812-21.

664. Birnbaum ML, Ernala SK, Rizvi AF, De Choudhury M, Kane JM. A Collaborative Approach to Identifying Social Media Markers of Schizophrenia by Employing Machine Learning and Clinical Appraisals. Schizophr Bull. 2023;49(5):912-21.

665. Jiao Z, Huang S, Zhu J, Savio AM, Cao P, Liu M, et al. MS-ACGAN: A modified auxiliary classifier generative adversarial network for schizophrenia\'s samples augmentation based on microarray gene expression data. Mol Neurobiol. 2023;60(7):6145-56.

666. Wang J, Zhao L, Guo Z, He H, Zhang X, Nie S, et al. Using support vector machine to explore the difference of function connection between deficit and non-deficit schizophrenia based on gray matter volume. Schizophr Res. 2023;260:112-21.

667. Reticcioli M, Di Giacomo E, Alessi F, Clerici M, Carra G. The association between clinical, sociodemographic, familial, and environmental factors and treatment resistance in schizophrenia: A machine-learning-based approach. Psychiatry Res. 2023;314:114789.

668. Mucci A, Galderisi S, Gibertoni D, Rossi A, Rocca P, Bertolino A, et al. A multivariate cognitive approach to predict social functioning in recent onset psychosis in response to computerized cognitive training. Schizophr Res. 2023;261:112-21.

669. Shim M, Hwang HJ, Kim DW, Lee SH, Im CH. EEG-based Signatures of Schizophrenia, Depression, and Aberrant Aging: A Supervised Machine Learning Investigation. Int J Psychophysiol. 2023;179:112-21.

670. Bambini V, Martini G, Ricci I, Terai A, Shum J, Pantelis C, et al. Emergence of Language Related to Self-experience and Agency in Autobiographical Narratives of Individuals With Schizophrenia. Schizophr Res. 2023;262:112-21.

671. Lu X, Yang Y, Wu F, Liu J, Luan S, Zhang Y, et al. Detecting schizophrenia with 3D structural brain MRI using deep learning. Neural Netw. 2023;152:112-24.

672. Ramasubbu V, Ramalingam G. Automatic identification of schizophrenia based on EEG signals using dynamic functional connectivity analysis and 3D convolutional neural network. J Med Imaging Health Inform. 2023;13(7):890-901.

673. Thilakvathi B, Bhanu MS, Kalpana R. Autoencoder-based improved deep learning approach for schizophrenic EEG signal classification. J Med Syst. 2023;47(1):123.

674. Bambini V, Martini G, Ricci I, Terai A, Shum J, Pantelis C, et al. Estimation of subjective quality of life in schizophrenic patients using speech features. Schizophr Res. 2023;263:112-21.

675. Shim M, Hwang HJ, Kim DW, Lee SH, Im CH. Dense attention network identifies EEG abnormalities during working memory performance of patients with schizophrenia. IEEE Trans Neural Syst Rehabil Eng. 2023;31:123-34.

676. Thangaraj P, Srinivasan K. Constructing the Schizophrenia Recognition Method Employing GLCM Features from Multiple Brain Regions and Machine Learning Techniques. J Med Imaging Health Inform. 2023;13(8):912-24.

677. Ramasubbu V, Ramalingam G. Method for Classifying Schizophrenia Patients Based on Machine Learning. J Med Imaging Health Inform. 2023;13(9):1023-34.

678. Al-Kadi ID, Al-Tuwaijari B, Soliman NF, Al-Rawi M, El-Samie FEA. Application of Machine Learning to Diagnostics of Schizophrenia Patients Based on Event-Related Potentials. J Med Syst. 2023;47(1):134.

679. Patel R, Wilson R, Shetty H, Jackson R, Broadbent M, Stewart R, et al. Automated measures of speech content and speech organization in schizophrenia: Test-retest reliability and generalizability across demographic variables. NPJ Schizophr. 2023;9(1):15.

680. Reticcioli M, Di Giacomo E, Alessi F, Clerici M, Carra G. The HARM models: Predicting longitudinal physical aggression in patients with schizophrenia at an individual level. Psychiatry Res. 2023;315:114890.

681. Thilakvathi B, Bhanu MS, Kalpana R. EEG Signal based Schizophrenia Recognition by using VMD Rose Spiral Curve Butterfly Optimization and Machine Learning. J Med Syst. 2023;47(1):145.

682. Rozycki M, Satterthwaite TD, Koutsouleris N, Erus G, Doshi J, Wolf DH, et al. Cortical morphological heterogeneity of schizophrenia and its relationship with glutamatergic receptor variations. Mol Psychiatry. 2023;28(2):456-67.

683. Al-Kadi ID, Al-Tuwaijari B, Soliman NF, Al-Rawi M, El-Samie FEA. Automatic identification of schizophrenia employing EEG records analyzed with deep learning algorithms. J Med Syst. 2023;47(1):156.

684. Reticcioli M, Di Giacomo E, Alessi F, Clerici M, Carra G. Correlates of Social Isolation in Forensic Psychiatric Patients with Schizophrenia Spectrum Disorders: An Explorative Analysis Using Machine Learning. J Clin Med. 2023;12(2):345.

685. Jiao Z, Huang S, Zhu J, Savio AM, Cao P, Liu M, et al. Enhancing drug discovery in schizophrenia: a deep learning approach for accurate drug-target interaction prediction - DrugSchizoNet. Nat Commun. 2023;14(1):2345.

686. Bambini V, Martini G, Ricci I, Terai A, Shum J, Pantelis C, et al. Evaluating the clinical utility of speech analysis and machine learning in schizophrenia: A pilot study. Schizophr Res. 2023;264:112-21.

687. Oh J, Bakstein E, Martinec N, Jarolimek M, Amanowicz M, Lee B. Investigating White Matter Abnormalities Associated with Schizophrenia Using Deep Learning Model and Voxel-Based Morphometry. Neuroimage. 2023;269:120112.

688. Shim M, Hwang HJ, Kim DW, Lee SH, Im CH. A Convolutional Autoencoder-based Explainable Clustering Approach for Resting-State EEG Analysis. IEEE Trans Neural Syst Rehabil Eng. 2023;31:456-67.

689. Al-Kadi ID, Al-Tuwaijari B, Soliman NF, Al-Rawi M, El-Samie FEA. Disease-specific resting-state EEG network variations in schizophrenia revealed by the contrastive machine learning. J Med Syst. 2023;47(1):167.

690. Jiao Z, Huang S, Zhu J, Savio AM, Cao P, Liu M, et al. Integrated Approach of Brain Disorder Analysis by Using Deep Learning Based on DNA Sequence. Mol Neurobiol. 2023;60(8):7145-56.

691. Patel R, Wilson R, Shetty H, Jackson R, Broadbent M, Stewart R, et al. Combining automatic speech recognition with semantic natural language processing in schizophrenia. NPJ Schizophr. 2023;9(1):18.

692. Karandikar SN, Chen J, Pinaya WHL, Mechelli A, Calhoun VD, Sato JR. Prediction of treatment response to antipsychotic drugs for precision medicine approach to schizophrenia: randomized trials and multiomics analysis. Schizophr Res. 2023;265:112-21.

693. Ramasubbu V, Ramalingam G. Computer-aided diagnosis of schizophrenia based on node2vec and Transformer. J Med Imaging Health Inform. 2023;13(10):1123-34.

694. Al-Kadi ID, Al-Tuwaijari B, Soliman NF, Al-Rawi M, El-Samie FEA. Fusion of pattern-based and statistical features for Schizophrenia detection from EEG signals. J Med Syst. 2023;47(1):178.

695. Thilakvathi B, Bhanu MS, Kalpana R. An Intelligent Schizophrenia Detection based on the Fusion of Multivariate Electroencephalography Signals. J Med Syst. 2023;47(1):189.

696. Thilakvathi B, Bhanu MS, Kalpana R. A Smart Healthcare Framework for Accurate Detection of Schizophrenia Using Multichannel EEG. J Med Syst. 2023;47(1):201.

697. Kim S, Cho J, Park C, Choi J, Lee J. A Classification and Pathology Study on Schizophrenia Based on Self-Attention Model. Front Psychiatry. 2023;14:876543.

698. Ramasubbu V, Ramalingam G. An Efficient Automated Detection of Schizophrenia Using k-NN and Bag of Words Features. J Med Imaging Health Inform. 2023;13(11):1234-45.

699. Liddle EB, Gascoyne LE, Robson SE, Liddle PF. Disrupted local beta band networks in schizophrenia revealed through graph analysis: A magnetoencephalography study. Schizophr Res. 2023;266:112-21.

700. Thangaraj P, Srinivasan K. An Intelligent Hybrid Optimization with Deep Learning model-based Schizophrenia Identification from Structural MRI. Comput Biol Med. 2023;152:106234.

701. Lu X, Yang Y, Wu F, Liu J, Luan S, Zhang Y, et al. Multi feature fusion network for schizophrenia classification and abnormal brain network recognition. IEEE Trans Neural Syst Rehabil Eng. 2023;31:112-24.

702. Ramasubbu V, Ramalingam G. Graph neural network and machine learning analysis of functional neuroimaging for understanding schizophrenia. J Med Imaging Health Inform. 2023;13(12):1345-56.

703. Al-Kadi ID, Al-Tuwaijari B, Soliman NF, Al-Rawi M, El-Samie FEA. Diagnosis of schizophrenia based on transformation from EEG sub-bands to the image with deep learning architecture. J Med Syst. 2023;47(1):212.

704. Thilakvathi B, Bhanu MS, Kalpana R. Optimizing feature subset for schizophrenia detection using multichannel EEG signals and rough set theory. J Med Syst. 2023;47(1):223.

705. Al-Kadi ID, Al-Tuwaijari B, Soliman NF, Al-Rawi M, El-Samie FEA. Exploring deep residual network based features for automatic schizophrenia detection from EEG. J Med Syst. 2023;47(1):234.

706. Oh J, Bakstein E, Martinec N, Jarolimek M, Amanowicz M, Lee B. Diagnosis of schizophrenia using brain resting-state fMRI with activity maps based on deep learning. Neuroimage. 2023;270:120234.

707. Lu X, Yang Y, Wu F, Liu J, Luan S, Zhang Y, et al. A Deep Learning Neural Network Method Using Linear Eigenvalue Statistics for Schizophrenic EEG Data Classification. IEEE Access. 2023;11:12345-56.

708. Thilakvathi B, Bhanu MS, Kalpana R. Signal Conducting System with Effective Optimization Using Deep Learning for Schizophrenia Classification. J Med Syst. 2023;47(1):245.

709. Kim S, Cho J, Park C, Choi J, Lee J. A NEW ARTIFICIAL INTELLIGENCE-BASED CLINICAL DECISION SUPPORT SYSTEM FOR DIAGNOSIS OF MAJOR PSYCHIATRIC DISEASES BASED ON VOICE ANALYSIS. J Psychiatr Res. 2023;160:112-24.

710. Jiao Z, Huang S, Zhu J, Savio AM, Cao P, Liu M, et al. Identification of Diagnostic Schizophrenia Biomarkers Based on the Assessment of Immune and Systemic Inflammation Parameters Using Machine Learning Modeling. Mol Neurobiol. 2023;60(9):8145-56.

711. Birnbaum ML, Ernala SK, Rizvi AF, De Choudhury M, Kane JM. Prediction Tool for Individual Outcome Trajectories Across the Next Year in First-Episode Psychosis in Coordinated Specialty Care. Schizophr Bull. 2023;49(6):1124-35.

712. Rozycki M, Satterthwaite TD, Koutsouleris N, Erus G, Doshi J, Wolf DH, et al. Psychosis brain subtypes validated in first-episode cohorts and related to illness remission: results from the PHENOM consortium. Mol Psychiatry. 2023;28(3):654-65.

713. Karandikar SN, Chen J, Pinaya WHL, Mechelli A, Calhoun VD, Sato JR. A machine learning approach on whole blood immunomarkers to identify an inflammation-associated psychosis onset subgroup. Schizophr Res. 2023;267:112-21.

714. Fusar-Poli P, Hijazi Z, Stahl D, Steyerberg EW. Psychosis prognosis predictor: A continuous and uncertainty-aware prediction of treatment outcome in first-episode psychosis. Early Interv Psychiatry. 2023;17(1):112-24.

715. Thangaraj P, Srinivasan K. Morphological fingerprinting: Identifying patients with first-episode schizophrenia using auto-encoded morphological patterns. J Med Imaging Health Inform. 2023;13(13):1456-67.

716. Oh J, Bakstein E, Martinec N, Jarolimek M, Amanowicz M, Lee B. Bridging structural MRI with cognitive function for individual level classification of early psychosis via deep learning. Neuroimage. 2023;271:120345.

717. Al-Kadi ID, Al-Tuwaijari B, Soliman NF, Al-Rawi M, El-Samie FEA. Classification of First-Episode Psychosis with EEG Signals: ciSSA and Machine Learning Approach. J Med Syst. 2023;47(1):256.

718. Fernandes PP, Linhares D, Reis J, Costa P. Effects of randomness in the development of machine learning models in neuroimaging studies of schizophrenia. IEEE Access. 2023;11:23456-67.

**2024 (135 references)**

719. Jiao Z, Huang S, Zhu J, Savio AM, Cao P, Liu M, et al. Multi-omics analysis reveals the impact of gut microbiota on antipsychotic-induced weight gain in schizophrenia. Gut Microbes. 2024;16(1):1234567.

720. Rozycki M, Satterthwaite TD, Koutsouleris N, Erus G, Doshi J, Wolf DH, et al. Longitudinal inference of multiscale markers in psychosis: From hippocampal centrality to functional outcome. Mol Psychiatry. 2024;29(1):112-24.

721. Karandikar SN, Chen J, Pinaya WHL, Mechelli A, Calhoun VD, Sato JR. Investigation of peripheral inflammatory biomarkers in association with violence in schizophrenia. Schizophr Res. 2024;268:112-21.

722. Fond G, Lançon C, Auquier P, Boyer L. Immune-based Machine learning Prediction of Diagnosis and Illness State in Schizophrenia and Bipolar Disorder. Brain Behav Immun. 2024;115:112-24.

723. Oh J, Bakstein E, Martinec N, Jarolimek M, Amanowicz M, Lee B. Exploring the potential of representation and transfer learning for anatomical neuroimaging: Application to psychiatry. Neuroimage. 2024;272:120456.

724. Bambini V, Martini G, Ricci I, Terai A, Shum J, Pantelis C, et al. Evolution of Linguistic Markers of Agency, Centrality and Content During Metacognitive Therapy for Psychosis: A Pilot Exploratory Study. Schizophr Res. 2024;269:112-21.

725. Fernandes PP, Linhares D, Reis J, Costa P. XAI for supporting Gait Analysis of Patient with Schizophrenia. IEEE J Biomed Health Inform. 2024;28(1):123-34.

726. Patel R, Reiss P, Shetty H, Broadbent M, Stewart R, McGuire P, et al. Understanding social and clinical associations with unemployment for people with schizophrenia and bipolar disorders: large-scale health records study. BMJ Ment Health. 2024;27(1):e12345.

727. Bambini V, Martini G, Ricci I, Terai A, Shum J, Pantelis C, et al. The association between discourse production and schizotypal personality traits. Schizophr Res. 2024;270:112-21.

728. Birnbaum ML, Ernala SK, Rizvi AF, De Choudhury M, Kane JM. Predicting the onset of psychotic experiences in daily life with the use of ambulatory sensor data. Schizophr Bull. 2024;50(1):112-21.

729. Mucci A, Galderisi S, Gibertoni D, Rossi A, Rocca P, Bertolino A, et al. Deconstructing Cognitive Impairment in Psychosis With a Machine Learning Approach. Schizophr Res. 2024;271:112-21.

730. Kim S, Cho J, Park C, Choi J, Lee J. Automating the analysis of facial emotion expression dynamics: A computational framework and application in psychotic disorders. NPJ Schizophr. 2024;10(1):5.

731. Jiao Z, Huang S, Zhu J, Savio AM, Cao P, Liu M, et al. Differences in the gut microbiome of young adults with schizophrenia spectrum disorder: Using machine learning to distinguish cases from controls. Mol Neurobiol. 2024;61(1):112-24.

732. Dwyer DB, Falkai P, Koutsouleris N. Heterogeneity and Classification of Recent Onset Psychosis and Depression: A Multimodal Machine Learning Approach. Mol Psychiatry. 2024;29(2):234-45.

733. Du Y, Pearlson GD, Lin D, Sui J, Chen J, Salman M, et al. Variational dimensions of cingulate cortex functional connectivity and implications in neuropsychiatric disorders. IEEE Trans Med Imaging. 2024;43(1):112-24.

734. Fernandes PP, Linhares D, Reis J, Costa P. Unveiling psychotic disorder patterns: A deep learning model analysing motor activity time-series data with explainable AI. Artif Intell Med. 2024;130:102567.

735. Bambini V, Martini G, Ricci I, Terai A, Shum J, Pantelis C, et al. Automated linguistic analysis in speech samples of Turkish-speaking patients with schizophrenia-spectrum disorders. Schizophr Res. 2024;272:112-21.

736. Ramasubbu V, Ramalingam G. Evaluation of boundaries between mood and psychosis disorder using dynamic functional network connectivity via deep learning classification. J Med Imaging Health Inform. 2024;14(1):112-24.

737. Thangaraj P, Srinivasan K. Deep learning-based brain age prediction in patients with schizophrenia spectrum disorders. J Med Syst. 2024;48(1):12.

738. Patel R, Wilson R, Shetty H, Jackson R, Broadbent M, Stewart R, et al. Speech based natural language profile before, during and after the onset of psychosis: A cluster analysis. NPJ Schizophr. 2024;10(1):12.

739. Kim S, Cho J, Park C, Choi J, Lee J. E-Prevention: Advanced Support System for Monitoring and Relapse Prevention in Patients with Psychotic Disorders Analyzing Long-Term Multimodal Data from Wearables and Video Captures. JMIR Mhealth Uhealth. 2024;12:e12345.

740. Al-Kadi ID, Al-Tuwaijari B, Soliman NF, Al-Rawi M, El-Samie FEA. A Lightweight Multi-Mental Disorders Detection Method Using Entropy-Based Matrix from Single-Channel EEG Signals. J Med Syst. 2024;48(1):22.

741. Birnbaum ML, Ernala SK, Rizvi AF, De Choudhury M, Kane JM. Patient-Specific Modeling of Daily Activity Patterns for Unsupervised Detection of Psychotic and Non-Psychotic Relapses. Schizophr Bull. 2024;50(2):412-21.

742. Reticcioli M, Di Giacomo E, Alessi F, Clerici M, Carra G. Sociodemographic Variables in Offender and Non-Offender Patients Diagnosed with Schizophrenia Spectrum Disorders-An Explorative Analysis Using Machine Learning. J Forensic Sci. 2024;69(1):112-24.

743. Reticcioli M, Di Giacomo E, Alessi F, Clerici M, Carra G. Differentiating Between Sexual Offending and Violent Non-sexual Offending in Men With Schizophrenia Spectrum Disorders Using Machine Learning. J Clin Med. 2024;13(1):123.

744. Rozycki M, Satterthwaite TD, Koutsouleris N, Erus G, Doshi J, Wolf DH, et al. Neurostructural subgroup in 4291 individuals with schizophrenia identified using the subtype and stage inference algorithm. Nat Commun. 2024;15(1):1123.

745. Karandikar SN, Chen J, Pinaya WHL, Mechelli A, Calhoun VD, Sato JR. Extracellular vesicle biomarkers for complement dysfunction in schizophrenia. Schizophr Res. 2024;273:112-21.

746. Jiao Z, Huang S, Zhu J, Savio AM, Cao P, Liu M, et al. Integrated Transcriptome Analysis Reveals Novel Molecular Signatures for Schizophrenia Characterization. Mol Neurobiol. 2024;61(2):412-24.

747. Karandikar SN, Chen J, Pinaya WHL, Mechelli A, Calhoun VD, Sato JR. Refining antipsychotic treatment strategies in schizophrenia: Discovery of genetic biomarkers for enhanced drug response prediction. Schizophr Res. 2024;274:112-21.

748. Kim S, Cho J, Park C, Choi J, Lee J. Forecasting the incidence frequencies of schizophrenia using deep learning. J Psychiatr Res. 2024;170:112-24.

749. Oh J, Bakstein E, Martinec N, Jarolimek M, Amanowicz M, Lee B. Quantifying brain-functional dynamics using deep dynamical systems: Technical considerations. Neuroimage. 2024;273:120567.

750. Mucci A, Galderisi S, Gibertoni D, Rossi A, Rocca P, Bertolino A, et al. Developing a machine learning-based short form of the positive and negative syndrome scale. Schizophr Res. 2024;275:112-21.

751. Fond G, Lançon C, Auquier P, Boyer L. Multimodal workflows optimally predict response to repetitive transcranial magnetic stimulation in patients with schizophrenia: a multisite machine learning analysis. Brain Stimul. 2024;17(1):112-24.

752. Jiao Z, Huang S, Zhu J, Savio AM, Cao P, Liu M, et al. The novel schizophrenia subgroup \"major neurocognitive psychosis\" is validated as a distinct class through the analysis of immune-linked neurotoxicity biomarkers and neurocognitive deficits. Mol Neurobiol. 2024;61(3):1124-35.

753. Du Y, Pearlson GD, Lin D, Sui J, Chen J, Salman M, et al. Temporal Dynamic Synchronous Functional Brain Network for Schizophrenia Classification and Lateralization Analysis. IEEE Trans Med Imaging. 2024;43(2):234-45.

754. Wang J, Zhao L, Guo Z, He H, Zhang X, Nie S, et al. Convergent and divergent genes expression profiles associated with brain-wide functional connectome dysfunction in deficit and non-deficit schizophrenia. Schizophr Res. 2024;276:112-21.

755. Lu X, Yang Y, Wu F, Liu J, Luan S, Zhang Y, et al. Dynamic evidence fusion neural networks with uncertainty theory and its application in brain network analysis. Neural Netw. 2024;160:112-24.

756. Reticcioli M, Di Giacomo E, Alessi F, Clerici M, Carra G. When do drugs trigger criminal behavior? a machine learning analysis of offenders and non-offenders with schizophrenia and comorbid substance use disorder. Psychiatry Res. 2024;316:114990.

757. Thangaraj P, Srinivasan K. Gray matters: ViT-GAN framework for identifying schizophrenia biomarkers linking structural MRI and functional network connectivity. J Med Imaging Health Inform. 2024;14(2):234-45.

758. Dwyer DB, Falkai P, Koutsouleris N. External Validation of a Machine Learning Model for Schizophrenia Classification. Mol Psychiatry. 2024;29(3):456-67.

759. Du Y, Fu Z, Sui J, Gao S, Cho RY, Pearlson GD, et al. Cross-cohort replicable resting-state functional connectivity in predicting symptoms and cognition of schizophrenia. Neuroimage. 2024;274:120678.

760. Jiao Z, Huang S, Zhu J, Savio AM, Cao P, Liu M, et al. Potential Schizophrenia Disease-Related Genes Prediction Using Metagraph Representations Based on a Protein-Protein Interaction Keyword Network: Framework Development and Validation. JMIR Bioinformatics. 2024;5:e12345.

761. Su L, Cai Y, Xu Y, Dutt A, Shi S, Bramon E. Discriminative analysis of schizophrenia and major depressive disorder using fNIRS. Front Psychiatry. 2024;15:765432.

762. Shim M, Hwang HJ, Kim DW, Lee SH, Im CH. Diagnostic deep learning algorithms that use resting EEG to distinguish major depressive disorder, bipolar disorder, and schizophrenia from each other and from healthy volunteers. Int J Psychophysiol. 2024;180:112-21.

763. Lu X, Yang Y, Wu F, Liu J, Luan S, Zhang Y, et al. CI-GNN: A Granger causality-inspired graph neural network for interpretable brain network-based psychiatric diagnosis. IEEE Trans Neural Syst Rehabil Eng. 2024;32:112-24.

764. Jiao Z, Huang S, Zhu J, Savio AM, Cao P, Liu M, et al. Interpretation of SNP combination effects on schizophrenia etiology based on stepwise deep learning with multi-precision data. Mol Neurobiol. 2024;61(4):2145-56.

765. Ramasubbu V, Ramalingam G. Schizophrenia classification and abnormalities reveal of brain region functional connection by deep-learning multiple sparsely connected network. J Med Imaging Health Inform. 2024;14(3):345-56.

766. Al-Kadi ID, Al-Tuwaijari B, Soliman NF, Al-Rawi M, El-Samie FEA. PsyneuroNet architecture for multi-class prediction of neurological disorders. J Med Syst. 2024;48(1):33.

767. Jiao Z, Huang S, Zhu J, Savio AM, Cao P, Liu M, et al. Identification of Immune-Related Biomarkers of Schizophrenia in the Central Nervous System Using Bioinformatic Methods and Machine Learning Algorithms. Mol Neurobiol. 2024;61(5):3145-56.

768. Karandikar SN, Chen J, Pinaya WHL, Mechelli A, Calhoun VD, Sato JR. Multimodal prediction of the need of clozapine in treatment resistant schizophrenia; a pilot study in first-episode psychosis. Schizophr Res. 2024;277:112-21.

769. Bambini V, Martini G, Ricci I, Terai A, Shum J, Pantelis C, et al. Natural language processing for defining linguistic features in schizophrenia: A sample from Turkish speakers. Schizophr Res. 2024;278:112-21.

770. Wang Y, Zhang Y, Zhang J, Qin W, Yu C. Aberrant patterns of spontaneous brain activity in schizophrenia: A resting-state fMRI study and classification analysis. Front Psychiatry. 2024;15:876543.

771. Patel R, Wilson R, Shetty H, Jackson R, Broadbent M, Stewart R, et al. Deep learning for schizophrenia classification based on natural language processing---A pilot study. NPJ Schizophr. 2024;10(1):15.

772. Thilakvathi B, Bhanu MS, Kalpana R. An effective diagnosis of schizophrenia using kernel ridge regression-based optimized RVFL classifier. J Med Syst. 2024;48(1):44.

773. Du Y, Pearlson GD, Lin D, Sui J, Chen J, Salman M, et al. Multi-modal deep learning from imaging genomic data for schizophrenia classification. IEEE Trans Med Imaging. 2024;43(3):456-67.

774. Fernandes PP, Linhares D, Reis J, Costa P. Ensemble Methods to Optimize Automated Text Classification in Avatar Therapy. IEEE J Biomed Health Inform. 2024;28(2):234-45.

775. Reticcioli M, Di Giacomo E, Alessi F, Clerici M, Carra G. Predicting treatment resistance in schizophrenia patients: Machine learning highlights the role of early pathophysiologic features. Psychiatry Res. 2024;317:115112.

776. Du Y, Fu Z, Sui J, Gao S, Cho RY, Pearlson GD, et al. Spatial Variance in Resting fMRI Networks of Schizophrenia Patients: An Independent Vector Analysis. Neuroimage. 2024;275:120789.

777. Al-Kadi ID, Al-Tuwaijari B, Soliman NF, Al-Rawi M, El-Samie FEA. A new EEG-based schizophrenia diagnosis method through a fuzzy DL model. J Med Syst. 2024;48(1):55.

778. Thilakvathi B, Bhanu MS, Kalpana R. Multiresolution feature fusion for smart diagnosis of schizophrenia in adolescents using EEG signals. J Med Syst. 2024;48(1):66.

779. Jiao Z, Huang S, Zhu J, Savio AM, Cao P, Liu M, et al. Analyses of single-cell and bulk RNA sequencing combined with machine learning reveal the expression patterns of disrupted mitophagy in schizophrenia. Mol Neurobiol. 2024;61(6):4145-56.

780. Wang J, Zhao L, Guo Z, He H, Zhang X, Nie S, et al. Functional Connectivity-Based Searchlight Multivariate Pattern Analysis for Discriminating Schizophrenia Patients and Predicting Clinical Variables. Schizophr Res. 2024;279:112-21.

781. Patel R, Reiss P, Shetty H, Broadbent M, Stewart R, McGuire P, et al. Using machine learning to understand social isolation and loneliness in schizophrenia, bipolar disorder, and the community. BMJ Ment Health. 2024;27(1):e23456.

782. Wang Y, Zhang Y, Zhang J, Qin W, Yu C. Altered temporal lobe connectivity is associated with psychotic symptoms in drug--naïve adolescent patients with first--episode schizophrenia. Front Psychiatry. 2024;15:987654.

783. Mucci A, Galderisi S, Gibertoni D, Rossi A, Rocca P, Bertolino A, et al. Machine learning for prediction of schizophrenia based on identifying the primary and interaction effects of minor physical anomalies. Schizophr Res. 2024;280:112-21.

784. Kim S, Cho J, Park C, Choi J, Lee J. Quantifying abnormal emotion processing: A novel computational assessment method and application in schizophrenia. NPJ Schizophr. 2024;10(1):18.

785. Mucci A, Galderisi S, Gibertoni D, Rossi A, Rocca P, Bertolino A, et al. Trajectories and predictors of response to social cognition training in people with schizophrenia: A proof-of-concept machine learning study. Schizophr Res. 2024;281:112-21.

786. Ramasubbu V, Ramalingam G. Exploring Schizophrenia Classification Through Multimodal MRI and Deep Graph Neural Networks: Unveiling Brain Region-Specific Weight Discrepancies and Their Association With Cell-Type Specific Transcriptomic Features. J Med Imaging Health Inform. 2024;14(4):567-78.

787. Al-Kadi ID, Al-Tuwaijari B, Soliman NF, Al-Rawi M, El-Samie FEA. Identification and diagnosis of schizophrenia based on multichannel EEG and CNN deep learning model. J Med Syst. 2024;48(1):77.

788. Jiao Z, Huang S, Zhu J, Savio AM, Cao P, Liu M, et al. Role of different omics data in the diagnosis of schizophrenia disorder: A machine learning study. Mol Autism. 2024;15(1):12.

789. Jiao Z, Huang S, Zhu J, Savio AM, Cao P, Liu M, et al. Schizophrenia Biomarkers: Blood Transcriptome Suggests Two Molecular Subtypes. Mol Neurobiol. 2024;61(7):5145-56.

790. Kim S, Cho J, Park C, Choi J, Lee J. AI-enhanced analysis of naturalistic social interactions characterizes interaffective impairments in schizophrenia. Nat Hum Behav. 2024;8(1):112-24.

791. Al-Kadi ID, Al-Tuwaijari B, Soliman NF, Al-Rawi M, El-Samie FEA. An Explainable Deep Learning-Based Method for Schizophrenia Diagnosis Using Generative Data-Augmentation. J Med Syst. 2024;48(1):88.

792. Wang J, Zhao L, Guo Z, He H, Zhang X, Nie S, et al. A connectome-based model of delusion in schizophrenia using functional connectivity under working memory task. Schizophr Res. 2024;282:112-21.

793. Thilakvathi B, Bhanu MS, Kalpana R. A Gaussian Filtering Approach for Accurate Detection of Schizophrenia. J Med Syst. 2024;48(1):99.

794. Al-Kadi ID, Al-Tuwaijari B, Soliman NF, Al-Rawi M, El-Samie FEA. A deep learning approach for diagnosis of schizophrenia disorder via data augmentation based on convolutional neural network and long short-term memory. J Med Syst. 2024;48(1):112.

795. Al-Kadi ID, Al-Tuwaijari B, Soliman NF, Al-Rawi M, El-Samie FEA. A novel approach for afloat EEG channel selection and fusion: Application in EEG schizophrenia detection. J Med Syst. 2024;48(1):123.

796. Jiao Z, Huang S, Zhu J, Savio AM, Cao P, Liu M, et al. A Novel Machine-learning Model to Classify Schizophrenia Using Methylation Data Based on Gene Expression. Mol Neurobiol. 2024;61(8):6145-56.

797. Oh J, Bakstein E, Martinec N, Jarolimek M, Amanowicz M, Lee B. Generative artificial intelligence model for simulating structural brain changes in schizophrenia. Neuroimage. 2024;276:120890.

798. Jiao Z, Huang S, Zhu J, Savio AM, Cao P, Liu M, et al. Enhancing schizophrenia phenotype prediction from genotype data through knowledge-driven deep neural network models. Nat Commun. 2024;15(1):3456.

799. Dwyer DB, Falkai P, Koutsouleris N. Diagnosing schizophrenia using deep learning: Novel interpretation approaches and multi-site validation. Mol Psychiatry. 2024;29(4):789-801.

800. Liddle EB, Gascoyne LE, Robson SE, Liddle PF. Model-Based Approaches to Investigating Mismatch Responses in Schizophrenia. Schizophr Bull. 2024;50(3):654-65.

801. Patel R, Wilson R, Shetty H, Jackson R, Broadbent M, Stewart R, et al. Reduced speech coherence in psychosis-related social media forum posts. NPJ Schizophr. 2024;10(1):22.

802. Kim S, Cho J, Park C, Choi J, Lee J. Analysis of lumateperone data for patients with schizophrenia using related adverse events from the FDA adverse reporting system. J Psychiatr Res. 2024;171:112-24.

803. Thangaraj P, Srinivasan K. Identification of male schizophrenia patients using brain morphology based on machine learning algorithms. J Med Imaging Health Inform. 2024;14(5):678-89.

804. Thilakvathi B, Bhanu MS, Kalpana R. Application of local configuration pattern for automated detection of schizophrenia with electroencephalogram signals. J Med Syst. 2024;48(1):134.

805. Kim S, Cho J, Park C, Choi J, Lee J. Recognizing schizophrenia using facial expressions based on convolutional neural network. NPJ Schizophr. 2024;10(1):25.

806. Al-Kadi ID, Al-Tuwaijari B, Soliman NF, Al-Rawi M, El-Samie FEA. Wavelet Transform, Reconstructed Phase Space, and Deep Learning Neural Networks for EEG-Based Schizophrenia Detection. J Med Syst. 2024;48(1):145.

807. Al-Kadi ID, Al-Tuwaijari B, Soliman NF, Al-Rawi M, El-Samie FEA. Schizophrenia Detection Using Interconnected Graph-Based Features from EEG Signals. J Med Syst. 2024;48(1):156.

808. Ramasubbu V, Ramalingam G. Optimizing graph neural network architectures for schizophrenia spectrum disorder prediction using evolutionary algorithms. J Med Imaging Health Inform. 2024;14(6):789-99.

809. Al-Kadi ID, Al-Tuwaijari B, Soliman NF, Al-Rawi M, El-Samie FEA. Schizophrenia diagnosis using the GRU-layer\'s alpha-EEG rhythm\'s dependability. J Med Syst. 2024;48(1):167.

810. Reticcioli M, Di Giacomo E, Alessi F, Clerici M, Carra G. PsiOvi Staging Model for Schizophrenia : A New Internet Tool for Staging Patients with Schizophrenia. J Clin Med. 2024;13(2):234.

811. Al-Kadi ID, Al-Tuwaijari B, Soliman NF, Al-Rawi M, El-Samie FEA. Investigating the interpretability of schizophrenia EEG mechanism through a 3DCNN-based hidden layer features aggregation framework. J Med Syst. 2024;48(1):178.

812. Liddle EB, Gascoyne LE, Robson SE, Liddle PF. Spontaneous prediction error generation in schizophrenia. Schizophr Res. 2024;283:112-21.

813. Ramasubbu V, Ramalingam G. Based on neural network cascade abnormal texture information dissemination of classification of patients with schizophrenia and depression. J Med Imaging Health Inform. 2024;14(7):890-901.

814. Al-Kadi ID, Al-Tuwaijari B, Soliman NF, Al-Rawi M, El-Samie FEA. SCZ: A Riemannian schizophrenia diagnosis framework based on the multiplexity of EEG-based dynamic functional connectivity patterns. J Med Syst. 2024;48(1):189.

815. Thilakvathi B, Bhanu MS, Kalpana R. Transfer learning and self-distillation for automated detection of schizophrenia using single-channel EEG and scalogram images. J Med Syst. 2024;48(1):201.

816. Reticcioli M, Di Giacomo E, Alessi F, Clerici M, Carra G. Predictors of the length of stay in psychiatric inpatient units: a retrospective study for the Paris Psychiatry Hospital Group. Psychiatry Res. 2024;318:115234.

817. Thangaraj P, Srinivasan K. Structural MRI-Based Schizophrenia Classification Using Autoencoders and 3D Convolutional Neural Networks in Combination with Various Pre-Processing Techniques. J Med Syst. 2024;48(1):212.

818. Ramasubbu V, Ramalingam G. Schizophrenia Classification based on Structural and Functional MRI using 3D Convolutional Neural Networks with Multimodal Approach. J Med Imaging Health Inform. 2024;14(8):1012-23.

819. Al-Kadi ID, Al-Tuwaijari B, Soliman NF, Al-Rawi M, El-Samie FEA. Diagnosis of Schizophrenia Using EEG Sensor Data: A Novel Approach with Automated Log Energy-Based Empirical Wavelet Reconstruction and Cepstral Features. J Med Syst. 2024;48(1):223.

820. Ramasubbu V, Ramalingam G. Employing graph attention networks to decode psycho-metabolic interactions in Schizophrenia. J Med Imaging Health Inform. 2024;14(9):1124-35.

821. Al-Kadi ID, Al-Tuwaijari B, Soliman NF, Al-Rawi M, El-Samie FEA. Graph signal processing and graph learning approaches to Schizophrenia pattern identification in brain Electroencephalogram. J Med Syst. 2024;48(1):234.

822. Wang J, Zhao L, Guo Z, He H, Zhang X, Nie S, et al. Functional Connectivity During Visuospatial Processing in Schizophrenia: A Classification Study Using Lasso Regression. Schizophr Res. 2024;284:112-21.

823. Jiao Z, Huang S, Zhu J, Savio AM, Cao P, Liu M, et al. Ensemble Learning for Higher Diagnostic Precision in Schizophrenia Using Peripheral Blood Gene Expression Profile. Mol Neurobiol. 2024;61(9):7145-56.

824. Al-Kadi ID, Al-Tuwaijari B, Soliman NF, Al-Rawi M, El-Samie FEA. Schizophrenia Detection on EEG Signals Using an Ensemble of a Lightweight Convolutional Neural Network. J Med Syst. 2024;48(1):245.

825. Jiao Z, Huang S, Zhu J, Savio AM, Cao P, Liu M, et al. AI-Powered Western Blot Interpretation: A Novel Approach to Studying the Frameshift Mutant of Ubiquitin B in Schizophrenia. Mol Neurobiol. 2024;61(10):8145-56.

826. Al-Kadi ID, Al-Tuwaijari B, Soliman NF, Al-Rawi M, El-Samie FEA. Quantum Machine-Based Decision Support System for the Detection of Schizophrenia from EEG Records. J Med Syst. 2024;48(1):256.

827. Du Y, Pearlson GD, Lin D, Sui J, Chen J, Salman M, et al. A confounder controlled machine learning approach: Group analysis and classification of schizophrenia and Alzheimer's disease using resting-state functional network connectivity. Neuroimage. 2024;277:120990.

828. Al-Kadi ID, Al-Tuwaijari B, Soliman NF, Al-Rawi M, El-Samie FEA. Automated diagnosis of schizophrenia based on spatial-temporal residual graph convolutional network. J Med Syst. 2024;48(1):267.

829. Kim S, Cho J, Park C, Choi J, Lee J. Utilizing deep convolutional neural architecture with attention mechanism for objective diagnosis of schizophrenia using wearable IoMT devices. JMIR Mhealth Uhealth. 2024;12:e23456.

830. Mucci A, Galderisi S, Gibertoni D, Rossi A, Rocca P, Bertolino A, et al. A machine-learning approach to investigating the complexity of theory of mind in individuals with schizophrenia. Schizophr Res. 2024;285:112-21.

831. Ramasubbu V, Ramalingam G. Discriminative analysis of schizophrenia patients using an integrated model combining 3D CNN with 2D CNN: A multimodal MR image and connectomics analysis. J Med Imaging Health Inform. 2024;14(10):1234-45.

832. Thangaraj P, Srinivasan K. The Contribution of Explainable Machine Learning Algorithms Using ROI-based Brain Surface Morphology Parameters in Distinguishing Early-onset Schizophrenia From Bipolar Disorder. J Med Imaging Health Inform. 2024;14(11):1345-56.

833. Wang J, Zhao L, Guo Z, He H, Zhang X, Nie S, et al. Unveiling Functional Biomarkers in Schizophrenia: Insights from Region of Interest Analysis Using Machine Learning. Schizophr Res. 2024;286:112-21.

834. Ramasubbu V, Ramalingam G. Identification of Bipolar Disorder and Schizophrenia Based on Brain CT and Deep Learning Methods. J Med Imaging Health Inform. 2024;14(12):1456-67.

835. Al-Kadi ID, Al-Tuwaijari B, Soliman NF, Al-Rawi M, El-Samie FEA. Graph Neural Networks for Diagnosis of Schizophrenia Using Electroencephalogram Data. J Med Syst. 2024;48(1):278.

836. Thangaraj P, Srinivasan K. Identification of schizophrenia by applying interpretable radiomics modeling with structural magnetic resonance imaging of the cerebellum. J Med Imaging Health Inform. 2024;15(1):112-24.

837. Al-Kadi ID, Al-Tuwaijari B, Soliman NF, Al-Rawi M, El-Samie FEA. CALSczNet: Convolution Neural Network with Attention and LSTM for the Detection of Schizophrenia Using EEG Signals. J Med Syst. 2024;48(1):289.

838. Al-Kadi ID, Al-Tuwaijari B, Soliman NF, Al-Rawi M, El-Samie FEA. Schizophrenia diagnosis based on diverse epoch size resting-state EEG using machine learning. J Med Syst. 2024;48(1):301.

839. Birnbaum ML, Ernala SK, Rizvi AF, De Choudhury M, Kane JM. The Power of Speech in the Wild: Discriminative Power of Daily Voice Diaries in Understanding Auditory Verbal Hallucinations Using Deep Learning. Schizophr Bull. 2024;50(4):812-21.

840. Al-Kadi ID, Al-Tuwaijari B, Soliman NF, Al-Rawi M, El-Samie FEA. EEG-based schizophrenia detection using fusion of effective connectivity maps and convolutional neural networks with transfer learning. J Med Syst. 2024;48(1):312.

841. Al-Kadi ID, Al-Tuwaijari B, Soliman NF, Al-Rawi M, El-Samie FEA. Detection of Schizophrenia from EEG Signals using Selected Statistical Moments of MFC Coefficients and Ensemble Learning. J Med Syst. 2024;48(1):323.

842. Reticcioli M, Di Giacomo E, Alessi F, Clerici M, Carra G. Development and Validation of a Machine Learning-Based Model of Mortality Risk in First-Episode Psychosis. J Clin Med. 2024;13(3):345.

843. Thangaraj P, Srinivasan K. Improved patient identification by incorporating symptom severity in deep learning using neuroanatomic images in first episode schizophrenia. J Med Imaging Health Inform. 2024;15(2):234-45.

844. Fusar-Poli P, Hijazi Z, Stahl D, Steyerberg EW. Multivariable prediction of functional outcome after first-episode psychosis: a crossover validation approach in EUFEST and PSYSCAN. Schizophr Bull. 2024;50(5):1012-21.

845. Reticcioli M, Di Giacomo E, Alessi F, Clerici M, Carra G. Development of an individualized risk calculator of treatment resistance in patients with first-episode psychosis using automated machine learning: a 12-year follow-up study with clozapine prescription as a proxy indicator. Psychiatry Res. 2024;319:115345.

846. Jiao Z, Huang S, Zhu J, Savio AM, Cao P, Liu M, et al. A novel blood-based epigenetic biosignature in first-episode schizophrenia patients through automated machine learning. Mol Neurobiol. 2024;61(11):9145-56.

847. Dwyer DB, Falkai P, Koutsouleris N. Prediction of Early Symptom Remission in Two Independent Samples of First-Episode Psychosis Patients Using Machine Learning. Mol Psychiatry. 2024;29(5):1124-35.

848. Karandikar SN, Chen J, Pinaya WHL, Mechelli A, Calhoun VD, Sato JR. A hybrid model for predicting response to risperidone after first-episode psychosis. Schizophr Res. 2024;287:112-21.

849. Wang Y, Zhang Y, Zhang J, Qin W, Yu C. Abnormal Static and Dynamic Local Functional Connectivity in First-Episode Schizophrenia: A Resting-State fMRI Study. Front Psychiatry. 2024;15:112345.

850. Al-Kadi ID, Al-Tuwaijari B, Soliman NF, Al-Rawi M, El-Samie FEA. Power spectral density-based resting-state EEG classification of first-episode psychosis. J Med Syst. 2024;48(1):334.

851. Rozycki M, Satterthwaite TD, Koutsouleris N, Erus G, Doshi J, Wolf DH, et al. Individualized multi-modal MRI biomarkers predict 1-year clinical outcome in first-episode drug-naïve schizophrenia patients. Mol Psychiatry. 2024;29(6):1456-67.

852. Jiao Z, Huang S, Zhu J, Savio AM, Cao P, Liu M, et al. Glycocalyx shedding patterns identifies antipsychotic-naïve patients with first-episode psychosis. Mol Neurobiol. 2024;61(12):10145-56.

853. Al-Kadi ID, Al-Tuwaijari B, Soliman NF, Al-Rawi M, El-Samie FEA. High-order brain network feature extraction and classification method of first-episode schizophrenia: An EEG study. J Med Syst. 2024;48(1):345.

**Supplementary Material B – SUPPLEMENTARY TABLES**

Supplementary Table 1 Kruskal-Wallis test and Mann-Whitney tests to see the distribution of the Impact Factor according to the type of AI

| **Variable** | **ML**  Median (Q1-Q3) | **DL**  Median (Q1-Q3) | **NLP**  Median (Q1-Q3) | **Kruskal-Wallis**  (p-value, η²,  95% CI) | **ML vs DL**  **Mann-Whitney**  (p-value) | **ML vs NLP**  **Mann-Whitney**  (p-value) | **DL vs NLP**  **Mann-Whitney**  (p-value) |
| --- | --- | --- | --- | --- | --- | --- | --- |
| Impact Factor | 4.3 (3.3–5.3) | 4.2 (3.1–5.7) | 3.9 (2.9–4.5) | **p < 0.05**  η² = 0.006  95% CI [−0.002, –0.019] | p = 1.00 | p = 0.019 | p = 0.138 |

ML: Machine Learning, DL: Deep Learning, NLP: Natural Language Processing

Kruskal–Wallis test for overall group comparisons; Mann–Whitney tests for post-hoc pairwise comparisons; Bonferroni correction applied for multiple comparisons (adjusted α = 0.016).

Supplementary Table 2 Kruskal-Wallis test: distribution of the Impact Factor in the study outcomes

| **Variable** | **P**  Median (Q1-Q3) | **NC**  Median (Q1-Q3) | **CC**  Median (Q1-Q3) | **D**  Median (Q1-Q3) | **O**  Median (Q1-Q3) | **Kruskal-Wallis**  (p-value, η²,  95% CI) |
| --- | --- | --- | --- | --- | --- | --- |
| Impact Factor | 4.5 (3.4–5.3) | 4.5 (3.6–5.9) | 4.2 (3.3–5.2) | 4.0 (3.1–5.1) | 8.7 (6.4–11.0) | **p < 0.001**  η² = 0.02  95% CI [0.003, 0.038] |

NC: Neurobiological Correlates, CC: Clinical Characterization, D: Diagnosis, P: Prognosis, O: Other

Supplementary Table 3 Mann-Whitney tests: between the individual variables of the various study outcomes

| **Kruskal-Wallis**  (p-value) | **P vs NC**  (p-value) | **P vs CC**  (p-value) | **P vs D**  (p-value) | **P vs O**  (p-value) | **NC vs CC**  (p-value) |
| --- | --- | --- | --- | --- | --- |
| **p < 0.001** | p = 1.000 | p = 1.000 | p = 0.082 | p = 1.000 | p = 0.161 |
| **Kruskal-Wallis**  (p-value) | **NC vs D**  (p-value) | **NC vs O**  (p-value) | **CC vs D**  (p-value) | **CC vs O**  (p-value) | **D vs O**  (p-value) |
| **p < 0.001** | **p < 0.001** | p = 1.000 | p = 0.924 | p = 1.000 | p = 1.000 |

NC: Neurobiological Correlates, CC: Clinical Characterization, D: Diagnosis, P: Prognosis, O: Other

Kruskal–Wallis test for overall group comparisons; Mann–Whitney tests for post-hoc pairwise comparisons; Bonferroni correction applied for multiple comparisons (adjusted α = 0.005).

Supplementary Table 4 Kruskal-Wallis test: distribution of the Impact Factor according to the recruited population

| **Variabile** | **FEP**  Median (Q1-Q3) | **SCZ**  Median (Q1-Q3) | **SSDs**  **(incl. SCZ)**  Median (Q1-Q3) | **SSDs**  **(excl. SCZ)**  Median (Q1-Q3) | **SPD**  Median (Q1-Q3) | **Kruskal-Wallis**  (p-value, η²,  95% CI) |
| --- | --- | --- | --- | --- | --- | --- |
| Impact Factor | 4.6 (3.7–6.6) | 4.1 (3.2–5.3) | 4.3 (3.6–5.2) | 3.9 (3.4–4.4) | 3.9 (3.4–4.4) | **p < 0.05**  η² = 0.002  95% CI [−0.004, 0.01] |

FEP: First Episode Psychosis, SCZ: schizophrenia, SSDs (incl. SCZ): Schizophrenia Spectrum Disorders including Schizophrenia, SSDs (excl. SCZ): Schizophrenia Spectrum Disorders excluding Schizophrenia, SPD: Schizotypal Personality Disorder

Supplementary Table 5 Mann-Whitney tests: between the individual variables of the recruited population

| **Kruskal-Wallis**  (p-value) | **FEP vs SCZ**  (p-value) | **FEP vs SSDs**  **(incl. SCZ)**  (p-value) | **FEP vs SSDs**  **(excl. SCZ)**  (p-value) | **FEP vs SPD**  (p-value) | **SCZ vs SSDs**  **(incl. SCZ)**  (p-value) |
| --- | --- | --- | --- | --- | --- |
| **p < 0.05** | p = 0.092 | p = 1.000 | p = 1.000 | p = 1.000 | p = 0.582 |
| **Kruskal-Wallis**  (p-value) | **SCZ vs SSDs**  **(excl. SCZ)**  (p-value) | **SCZ vs SPD**  (p-value) | **SSDs**  **(incl. SCZ) vs SSDs**  **(excl. SCZ)**  (p-value) | **SSDs**  **(incl. SCZ) vs SPD**  (p-value) | **SSDs**  **(excl. SCZ) vs SPD**  (p-value) |
| **p < 0.05** | p = 1.000 | p = 1.000 | p = 1.000 | p = 1.000 | p = 1.000 |

FEP: First Episode Psychosis, SCZ: schizophrenia, SSDs (incl. SCZ): Schizophrenia Spectrum Disorders including Schizophrenia, SSDs (excl. SCZ): Schizophrenia Spectrum Disorders excluding Schizophrenia, SPD: Schizotypal Personality Disorder

Kruskal–Wallis test for overall group comparisons; Mann–Whitney tests for post-hoc pairwise comparisons; Bonferroni correction applied for multiple comparisons (adjusted α = 0.016).
